# Comparative effectiveness of exercise modalities and doses on carotid-femoral pulse wave velocity in adults: a systematic review and network meta-analysis of randomized controlled trials

**DOI:** 10.3389/fcvm.2026.1860243

**Published:** 2026-07-28

**Authors:** Yanli Tan, Shiqi Liu, Liuhong Zang

**Affiliations:** School of Physical Education, Xinjiang Normal University, Urumqi, Xinjiang, China

**Keywords:** arterial stiffness, carotid-femoral pulse wave velocity, exercise dose, network meta-analysis, randomized controlled trials

## Abstract

**Purpose:**

To compare the effects of exercise modalities and exercise dose on carotid-femoral pulse wave velocity (cfPWV) in adults using pairwise meta-analysis, network meta-analysis, and dose-response analysis.

**Methods:**

PubMed/MEDLINE, Cochrane Library, Web of Science, EBSCO, Embase, and Scopus were searched from inception to 28 January 2026 for parallel-group randomized controlled trials in adults reporting cfPWV. Trials evaluating seven exercise modalities were included. Pairwise meta-analysis, frequentist network meta-analysis, and Bayesian dose-response analysis were performed. Risk of bias was assessed using the Cochrane Risk of Bias 2 (RoB 2) tool, and certainty of evidence was assessed using the Grading of Recommendations, Assessment, Development, and Evaluation (GRADE) approach.

**Results:**

Sixty-nine studies involving 3,422 participants were included. Overall, exercise reduced cfPWV vs. control (WMD = −0.74 m/s, 95% CI −1.02 to −0.46), although heterogeneity was considerable. In the network meta-analysis, aerobic training, combined training, and interval training reduced cfPWV vs. non-exercise control, but no active modality was statistically superior to another. Certainty of evidence was moderate for combined training, low for aerobic training, and very low for interval training vs. control. Bayesian dose-response modelling suggested a nonlinear, model-derived association, with greater uncertainty at the extremes of the dose distribution.

**Conclusion:**

Exercise training was associated with lower cfPWV in adults, with the most consistent certainty supporting combined training. Comparative rankings and dose-response estimates should be interpreted as exploratory because of heterogeneity, initial network inconsistency, prediction intervals crossing the null, and model-dependent dose estimation.

**Systematic Review Registration:**

https://www.crd.york.ac.uk/prospero/display_record.php?ID=CRD420261355633, PROSPERO identifier CRD420261355633.

## 1 Introduction

Arterial stiffness (AS) is an important marker of vascular aging and an independent predictor of future cardiovascular risk (1, 2). Among the available indices, carotid-femoral pulse wave velocity (cfPWV) is widely recognized as the gold-standard noninvasive measure of central arterial stiffness because it directly reflects aortic stiffness and has clear prognostic value (1). A previous meta-analysis further showed that each 1 m/s increase in aortic PWV was associated with a 14% increase in cardiovascular events, a 15% increase in cardiovascular mortality, and a 15% increase in all-cause mortality (2). Therefore, identifying effective non-pharmacological strategies to reduce cfPWV is of clear clinical importance.

Exercise is a core strategy for improving cardiovascular health and is strongly recommended in current physical activity guidelines. The World Health Organization recommends 150–300 min/week of moderate-intensity aerobic physical activity, 75–150 min/week of vigorous-intensity activity, or an equivalent combination, together with muscle-strengthening activities (3). However, vascular responses may differ across modalities. Aerobic training may improve endothelial function through repeated shear-stress stimuli, whereas interval training produces intermittent higher haemodynamic stimuli; resistance training responses may depend on loading characteristics and participant factors (4–6). Stretching was included because repeated musculo-tendinous stretching may improve endothelial function and arterial stiffness (7). Age, sex, adiposity, baseline cfPWV, and cardiometabolic status may influence both baseline stiffness and responsiveness to exercise and were therefore considered potential effect modifiers. The optimal weekly dose for improving cfPWV remains unclear (8).

Several systematic reviews have examined exercise and arterial stiffness, but direct head-to-head comparisons are limited, and conventional pairwise meta-analysis cannot jointly synthesize direct and indirect evidence across multiple modalities. Network meta-analysis can compare multiple modalities within one framework, whereas dose-response analysis can evaluate nonlinear associations across continuous exposure levels. Previous reviews also often pooled different pulse wave velocity indices or broader vascular outcomes rather than cfPWV and rarely quantified exercise dose as a continuous exposure. We expressed weekly exposure as MET·min/week to harmonize intensity, session duration, and frequency across trials, although this approximation is less precise for resistance-based and combined programmes.

Accordingly, we integrated pairwise meta-analysis to estimate overall and direct effects, frequentist network meta-analysis to compare exercise modalities, and Bayesian model-based dose-response analysis to assess dose-response. We hypothesized that exercise would reduce cfPWV vs. non-exercise control and that aerobic, combined, and interval training would show favourable effects vs. control; relative differences between active modalities and modality-specific dose-response patterns were considered exploratory.

## 2 Methods

### 2.1 Registration

This systematic review and network meta-analysis was prospectively registered in PROSPERO (registration number: CRD420261355633) and was conducted and reported in accordance with the Preferred Reporting Items for Systematic Reviews and Meta-Analyses extension statement for reporting systematic reviews incorporating network meta-analyses (9) and the Cochrane Handbook for Systematic Reviews of Interventions (10).

### 2.2 Search strategy

We systematically searched the following electronic databases: PubMed/MEDLINE, Cochrane Library, Web of Science, EBSCO, Embase, and Scopus, from database inception to 28 January 2026, to identify eligible studies. The search was restricted to English-language publications because of feasibility constraints. This restriction may have introduced language bias and was considered when interpreting the findings. The search strategy was developed by combining controlled vocabulary terms (e.g., MeSH terms) and free-text terms with Boolean operators and was structured around the following key concepts: adult populations, arterial stiffness-related outcomes, exercise interventions, and randomized controlled trials. The terms used included, but were not limited to, “arterial stiffness”, “vascular stiffness”, “pulse wave velocity”, “cfPWV”, “exercise”, “physical activity”, “aerobic”, “resistance”, “interval training”, and “randomized controlled trial”. The full search strategies for all databases are provided in Supplementary Appendix 1.

### 2.3 Eligibility criteria

Eligible studies were English-language full-text parallel-group randomised controlled trials that evaluated exercise training effects on cfPWV in adults aged ≥18 years. Eligible participants included general adult populations; adults with cardiometabolic risk factors, including overweight/obesity, hypertension, type 2 diabetes, metabolic syndrome, or dyslipidaemia; and adults with clinical conditions associated with elevated cardiovascular risk, including coronary artery disease, heart failure, chronic kidney disease, haemodialysis, kidney transplantation, or cerebrovascular disease. Eligible interventions were aerobic training (AT; continuous rhythmic exercise engaging large muscle groups), resistance training (RT), interval training (IT; planned alternating work and recovery periods), combined training (CT; planned programmes incorporating both aerobic and resistance components), stretching training (ST), isometric handgrip training (IHT), and whole-body vibration training (WBVT). Resistance training comprised planned exercises using external resistance to improve muscular strength or endurance; stretching training comprised structured flexibility protocols centred on sustained muscle–tendon stretching; isometric handgrip training comprised repeated static handgrip contractions; and whole-body vibration training comprised exercise or standing performed on a mechanically vibrating platform. Comparators could be non-exercise controls or another eligible exercise modality. To minimise confounding, studies including participants with regular exercise within the previous 3 months were excluded; regular exercise was defined as ≥3 sessions/week, ≥30 min/session, at moderate intensity.

Excluded were non-randomised, crossover, cluster-randomised, quasi-experimental, and single-arm studies; animal studies; studies of children or adolescents, athletes, endurance-trained individuals, or acute single-session exercise; studies in which the independent effect of exercise could not be isolated; studies not reporting eligible cfPWV data; and duplicate publications, conference abstracts, reviews, letters, meta-analyses, or reports with insufficient extractable data.

### 2.4 Study selection and data extraction

All records were imported into Zotero 8 for reference management and duplicate removal. Study selection was performed independently by two reviewers (Y.T. and S.L.), who screened titles and abstracts and then assessed potentially eligible full texts. Disagreements were resolved by discussion, with consultation of a third reviewer (L.Z.) when necessary. The same two reviewers independently extracted study reference, country, design, participant characteristics, intervention and comparator characteristics, and cfPWV outcome data using a standardised form. When numerical outcome data were unavailable in the text or tables, values were digitised from figures using WebPlotDigitizer (version 4.8; Automeris, Pacifica, CA, USA) independently by both reviewers and resolved by consensus. Included-study characteristics are reported in Supplementary Appendix 2.

Pairwise meta-analyses of exercise-vs.-non-exercise control comparisons were performed as the primary direct analyses. For multi-arm trials sharing a non-exercise control, the control-group sample size was divided equally, or as nearly equally as possible, across eligible comparisons while the control mean and standard deviation were retained. Active-vs.-active comparisons were retained for the network meta-analysis and, when directly available within a trial, were additionally synthesized in comparison-specific direct pairwise meta-analyses for presentation in Table 1. For each active-vs.-active comparison, only the relevant randomized arms were included. All eligible arms of multi-arm trials were entered jointly in the network model, retaining the correlation induced by shared comparators.

**Table 1:** Direct pairwise and network meta-analysis estimates for carotid-femoral pulse wave velocity (cfPWV).

AT	RT	CT	IT	WBVT	IHT	ST	CON
**AT**	0.27 (−0.20 to 0.74)	−0.07 (−0.44 to 0.30)	0.02 (−0.15 to 0.19)	NA	NA	0.15 (−0.48 to 0.78)	**−0.85 (−1.19 to −0.52)**
−0.49 (−1.04 to 0.06)	**RT**	1.17 (−1.66 to 3.99)	NA	NA	NA	−0.60 (−1.86 to 0.66)	−0.36 (−0.73 to 0.01)
0.12 (−0.42 to 0.67)	0.62 (−0.00 to 1.23)	**CT**	NA	NA	NA	−0.54 (−1.76 to 0.68)	**−0.49 (−0.78 to −0.20)**
0.20 (−0.32 to 0.72)	0.69 (−0.01 to 1.39)	0.08 (−0.62 to 0.77)	**IT**	NA	NA	NA	−1.19 (−2.55 to 0.18)
−0.08 (−1.21 to 1.05)	0.41 (−0.77 to 1.60)	−0.20 (−1.39 to 0.99)	−0.28 (−1.48 to 0.92)	**WBVT**	NA	NA	**−0.40 (−0.64 to −0.16)**
−0.38 (−1.84 to 1.08)	0.11 (−1.39 to 1.61)	−0.50 (−2.00 to 1.00)	−0.58 (−2.09 to 0.93)	−0.30 (−2.08 to 1.48)	**IHT**	NA	−0.36 (−1.61 to 0.88)
−0.04 (−0.89 to 0.80)	0.45 (−0.52 to 1.42)	−0.17 (−1.13 to 0.79)	−0.24 (−1.23 to 0.74)	0.03 (−1.37 to 1.44)	0.34 (−1.34 to 2.01)	**ST**	NA
**−0.74 (−1.07 to −0.40)**	−0.24 (−0.74 to 0.25)	**−0.86 (−1.35 to −0.37)**	**−0.94 (−1.46 to −0.41)**	−0.66 (−1.74 to 0.42)	−0.36 (−1.78 to 1.06)	−0.69 (−1.59 to 0.20)	**CON**

The data shown are weighted mean differences (WMDs) and 95% confidence intervals (CIs); lower cfPWV indicates a more favourable effect. Network meta-analysis results are in blue and represent column minus row; negative values favour the column intervention. Pairwise meta-analysis results are in yellow and represent row minus column; negative values favour the row intervention. Bold indicates significant comparisons.

AT, aerobic training; RT, resistance training; CT, combined training; IT, interval training; WBVT, whole-body vibration training; IHT, isometric handgrip training; ST, stretching training; CON, non-exercise control; NA, not available.

### 2.5 Categorization of the interventions' available evidence

Exercise intensity was classified as light, moderate, or vigorous using American College of Sports Medicine thresholds (11). For aerobic and interval protocols, the cut-offs were <40%, 40%–59%, and ≥60% heart-rate reserve (HRR) or oxygen-uptake reserve; <64%, 64%–76%, and ≥77% maximum heart rate (HRmax); <46%, 46%–63%, and ≥64% maximal oxygen uptake (VO₂max); or Borg 6–20 rating of perceived exertion (RPE) <12, 12–13, and ≥14, respectively. For resistance training, loads of <50%, 50%–69%, and ≥70% one-repetition maximum (%1RM) were classified as light, moderate, and vigorous, respectively. For interval training, classification was based on the intensity of the work intervals rather than the recovery periods. For combined training, classification followed the reported overall programme intensity; when this was unavailable and component intensities differed, it was determined from the time-weighted average intensity of the aerobic and resistance components. When no quantitative intensity indicator or author-reported intensity descriptor was available, intensity was recorded as not reported.

### 2.6 Outcome definition

The primary outcome was carotid-femoral pulse wave velocity (cfPWV). Studies reporting “aortic PWV” were eligible only when the original article explicitly stated that PWV was measured between the carotid and femoral arterial sites or explicitly described the outcome as cfPWV. Studies reporting unspecified aortic PWV, brachial–ankle PWV, femoral–ankle PWV, or other regional indices were excluded. cfPWV was selected because it is widely considered the gold-standard measure of central arterial stiffness (1, 12).

### 2.7 Data synthesis

Effect sizes were synthesized using pre-to-post changes in the experimental and control groups to minimize the influence of baseline differences. When change scores were not directly reported, mean changes were calculated from baseline and post-intervention values. The corresponding standard deviations of change were derived according to the Cochrane Handbook using the formula (10), where SD denotes standard deviation:

$$\mathrm{SDchange}=\sqrt{\left(\mathrm{SDbaselin}e^{2}+\mathrm{SDpos}t^{2}-2\;\times \;r\;\times \;\mathrm{SDbaseline}\;\times \;\mathrm{SDpost}\right)}.$$

When the within-group correlation coefficient (*r*) was not reported, *r* = 0.5 was used as a pragmatic mid-range assumption for the primary analysis. Pairwise and network meta-analyses were repeated using *r* = 0.3 and *r* = 0.7 to assess the robustness of this assumption. Outcomes reported as medians, ranges, or interquartile ranges were converted to means and standard deviations using established methods (13).

### 2.8 Statistical analyses

#### 2.8.1 Pairwise meta-analysis

Pairwise meta-analyses used a DerSimonian-Laird random-effects model as the primary analysis. Restricted maximum likelihood and Hartung-Knapp-Sidik-Jonkman adjustments were applied as sensitivity analyses. Direct active-vs.-active comparisons were synthesized separately when available; single-study comparisons were reported as study-specific WMDs with 95% CIs.

Because cfPWV was consistently reported in metres per second (m/s), pooled effects were expressed as weighted mean differences (WMDs) with 95% confidence intervals (CIs). Statistical heterogeneity was assessed using I², with values of 0%–30%, 30%–50%, 50%–75%, and 75%–100% interpreted as not important, moderate, substantial, and considerable, respectively; corresponding *p* values were also considered (14). Sensitivity analyses were conducted by sequentially excluding influential studies identified using the Baujat plot. Subgroup analyses and meta-regression of age, sex, body mass index, baseline cfPWV, population type, intervention intensity, and intervention duration were prespecified in PROSPERO to explore heterogeneity. Publication bias was assessed using Egger's test and funnel-plot symmetry, with *p* < 0.10 indicating potential small-study effects (15, 16).

#### 2.8.2 Network meta-analysis

A random-effects network meta-analysis under a frequentist framework synthesized direct and indirect evidence to estimate the relative effects of discrete exercise modalities (17). The primary network meta-analysis was based on the original network including all eligible studies. The treatment network was illustrated with node size proportional to sample size and edge thickness proportional to the number of direct comparisons. Consistency was assessed using the global inconsistency model, loop-specific inconsistency factors with 95% confidence intervals, and node-splitting analyses (18). Where inconsistency was detected, its potential sources were explored through complementary diagnostic procedures and sensitivity analyses. A sensitivity network meta-analysis excluding Liu and Zhu (19) was conducted to assess the influence of this study on network consistency, relative treatment effects, and ranking estimates; this analysis did not replace the primary full-network analysis. Interventions were ranked using the surface under the cumulative ranking curve (SUCRA) (17, 20), and comparison-adjusted funnel plots assessed potential small-study effects. Transitivity was assessed descriptively by comparing potential effect modifiers across intervention groups (21). An interval plot based on the primary consistency model presented pooled estimates with 95% CIs and 95% prediction intervals for all pairwise comparisons.

#### 2.8.3 Dose-response analysis

The frequentist network meta-analysis was used to compare the relative effects of discrete exercise modalities, whereas the Bayesian model-based network meta-analysis was used to model continuous and potentially nonlinear dose-response relationships across intervention arms. These approaches therefore addressed complementary questions rather than serving as alternative estimators of the same treatment effect. Exercise dose was expressed as MET·min/week. Reported %HRR, %HRmax, %VO₂max, RPE, and %1RM values were used to classify intervention intensity rather than being mathematically converted to MET values. For each active arm, a MET value was assigned by matching the reported activity mode and protocol description to the closest applicable entry in the 2024 Adult Compendium of Physical Activities (22). For CT and IT, session MET values were calculated as time-weighted averages of component-specific values or work and recovery phases, respectively. Weekly dose was calculated as MET × session duration × weekly frequency. Study-arm-level MET assignments and dose calculations are provided in Supplementary Appendix 3. The underlying assignment rules were based on the 2024 Adult Compendium of Physical Activities. Arms with insufficient information on session duration, weekly frequency, or intervention characteristics to derive a weekly dose were retained in pairwise and network meta-analyses where eligible but excluded from dose-response analysis. For resistance-based and combined interventions, estimated dose may partly reflect total session duration rather than pure active contraction time. Dose-response was examined using a random-effects Bayesian model-based network meta-analysis (MBNMA) (23). Before model fitting, network connectivity, consistency, and transitivity were assessed (24–26). Emax, restricted cubic spline, and quadratic models were compared using the Deviance Information Criterion (DIC) and fitted plots. The quadratic random-effects model was selected because it had a slightly lower DIC than the restricted cubic spline model and provided a more parsimonious, interpretable description of the nonlinear pattern (27). Mean difference (MD) was the effect measure, and 95% credible intervals (CrIs) quantified uncertainty; estimates were considered statistically significant when 95% CrIs excluded 0 (28). Pairwise and network meta-analyses were conducted in Stata 17.0; dose-response analyses were conducted in R using MBNMAdose, and figures were generated using ggplot2.

### 2.9 Risk of bias and certainty assessment

Two reviewers (Y.T. and S.L.) independently assessed risk of bias using the Cochrane RoB 2 tool (29), and disagreements were resolved by discussion, with consultation of a third reviewer (L.Z.) when necessary. The five domains were bias arising from the randomization process, deviations from intended interventions, missing outcome data, measurement of the outcome, and selection of the reported result. Each domain and the overall judgement were rated as low risk, some concerns, or high risk. Detailed assessments are provided in Supplementary Appendix 4.

The Grading of Recommendations, Assessment, Development, and Evaluation (GRADE) approach was used to assess the certainty of the evidence contributing to the network estimates. Each comparison was rated as high, moderate, low, or very low according to study limitations, inconsistency, indirectness, imprecision, and publication bias (30, 31). Detailed comparison-level GRADE assessments are provided in Supplementary Appendix 17.

## 3 Results

### 3.1 Study selection

**Figure 1:**
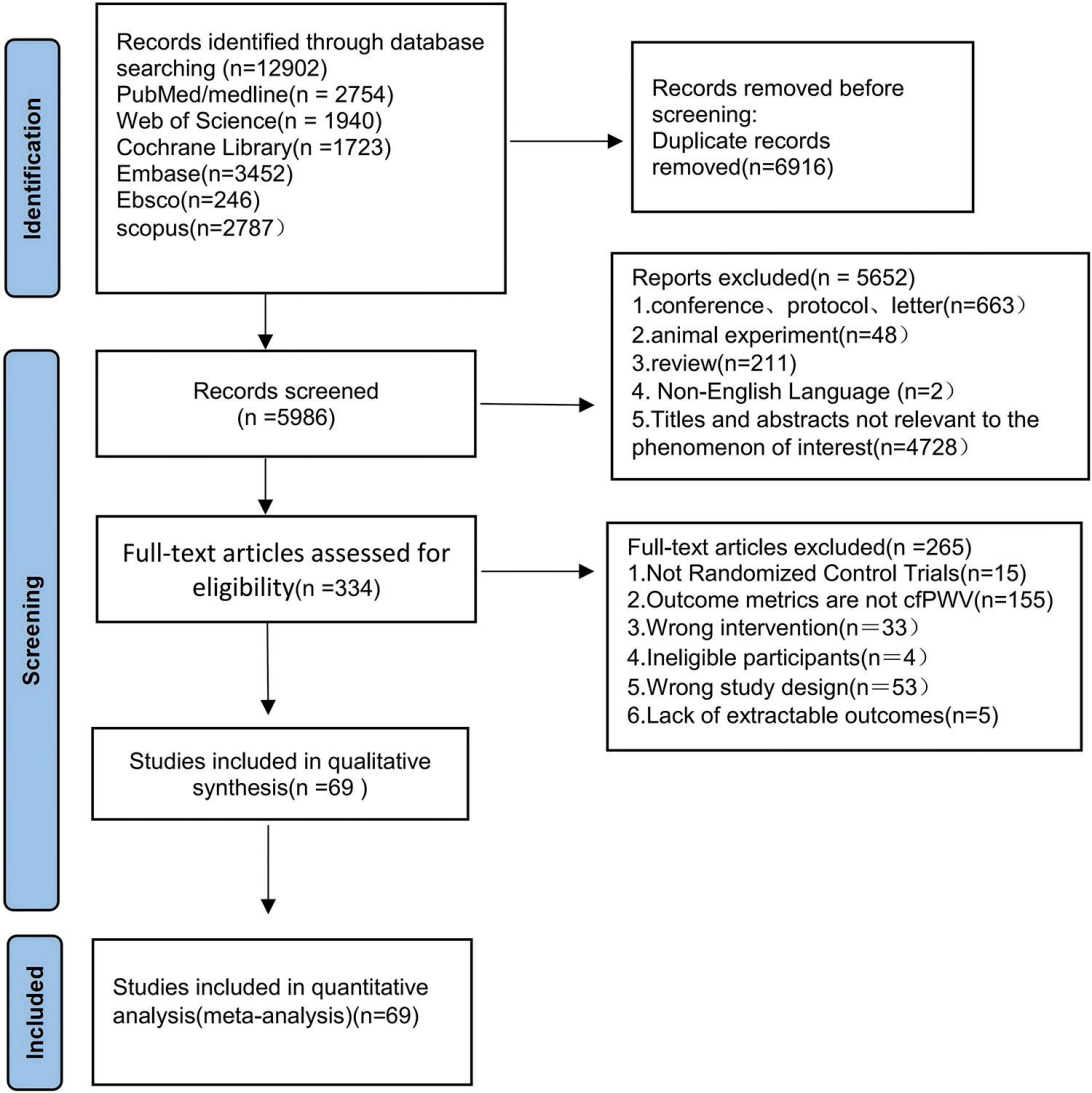
Preferred reporting items for systematic reviews and meta-analyses (PRISMA) flow diagram of each stage of the study selection.

As shown in Figure 1, a total of 12,902 records were identified through database searching. After removal of 6,916 duplicates, 5,986 records remained for screening. Following title/abstract screening and full-text assessment, 69 studies were finally included in both the qualitative and quantitative syntheses (19, 32–99). The main reasons for full-text exclusion were non-cfPWV outcomes, wrong study design, wrong intervention, ineligible participants, and insufficient extractable data.

### 3.2 Characteristics of the included studies

The characteristics of the included studies are summarized in Supplementary Appendix 2. A total of 69 studies involving 3,422 participants were included, contributing 92 study-level direct treatment comparisons to the quantitative synthesis. Overall, aerobic training was the most frequently investigated intervention, followed by combined training, resistance training, and interval training, whereas WBVT and IHT were less commonly reported. The intervention period ranged from 3 to 52 weeks. Among the intervention groups with available duration data, 66 study populations (90.4%) lasted at least 8 weeks. Exercise frequency ranged from 2 to 7 sessions per week; among the 67 study populations that reported weekly exercise frequency, 62 (92.5%) involved 3 or more sessions per week. Session duration ranged from 10 to 60 min per session; among the intervention groups with available session-duration data, 48 (84.2%) lasted at least 30 min per session. Baseline cfPWV values ranged from 5.0 to 16.3 m/s, indicating that the included populations covered a broad spectrum of arterial stiffness severity.

### 3.3 Results of RoB 2 assessment

The RoB 2 assessment is summarized in Supplementary Appendices 4, 5. Overall, 44 studies were judged to have low risk of bias, 12 raised some concerns, and 13 were judged to have high risk of bias.

### 3.4 Direct pairwise meta-analyses

#### 3.4.1 Primary outcome

Direct pairwise meta-analysis results are presented in the upper-right triangle of Table 1. The forest plot for exercise-vs.-control comparisons is provided in Supplementary Appendix 7, and forest plots for all eligible direct comparisons are provided in Supplementary Appendix 11. Overall, exercise interventions significantly reduced cfPWV compared with the control group (WMD = −0.74, 95% CI: −1.02 to −0.46), although substantial heterogeneity was observed (I² = 89.4%). By exercise modality, significant reductions in cfPWV were found for aerobic training (WMD = −0.85, 95% CI: −1.19 to −0.52; I² = 80.9%), combined training (WMD = −0.49, 95% CI: −0.78 to −0.20; I² = 21.2%), and whole-body vibration training (WMD = −0.40, 95% CI: −0.64 to −0.16; I² = 0.0%). In contrast, resistance training (WMD = −0.36, 95% CI: −0.73 to 0.01; I² = 44.4%), interval training (WMD = −1.19, 95% CI: −2.55 to 0.18; I² = 97.4%), and isometric handgrip training (WMD = −0.36, 95% CI: −1.61 to 0.88; I² = 52.7%) did not show statistically significant effects. No direct ST-vs.-CON comparison was available; ST entered the network through active-comparator trials and was therefore evaluated primarily through indirect network evidence. Direct head-to-head comparisons between active exercise modalities were available for seven contrasts, and none showed a statistically significant difference. Several individual study estimates were large in magnitude, including those from comparison b of O'Gorman et al. (41), Lai et al. (34), Ghardashi-Afousi et al. (32), comparison a of Greenwood et al. (81), and comparison b of Morgan et al. (43). The extracted values were rechecked against the source reports and retained because they met the prespecified eligibility criteria; however, their magnitude contributed to the substantial heterogeneity and warrants cautious interpretation of the pooled estimate. Egger's regression test showed no significant publication bias (*P* = 0.258), and the corresponding funnel plot is presented in Supplementary Appendix 6.

#### 3.4.2 Heterogeneity test

Sequential exclusion of influential studies did not reverse the direction of the pooled exercise-vs.-control effect. After exclusion of Ghardashi-Afousi et al. (32), the pooled estimate remained significant (WMD = −0.64, 95% CI: −0.82 to −0.46), although substantial residual heterogeneity persisted (I² = 70.2%). Further exclusion of Kim et al. (56) and Gómez-Sánchez et al. (52) produced similar pooled estimates with only limited additional reductions in heterogeneity. Sensitivity analyses using alternative within-group correlation coefficients (*r* = 0.3 and *r* = 0.7), as well as alternative random-effects estimators, retained the direction of the overall effect. These findings indicate that the pooled association was not attributable solely to a single influential study, but that the magnitude of effect varied materially across studies. Detailed sensitivity analyses are provided in Supplementary Appendix 8.

Exploratory subgroup and meta-regression analyses are summarized in Tables 2–4. Sex showed a statistically significant between-subgroup difference, and study-level baseline cfPWV and BMI were associated with the magnitude of cfPWV change in multivariable meta-regression. However, substantial residual heterogeneity remained after adjustment. These analyses were based on aggregate study-level characteristics and should therefore be interpreted as hypothesis-generating signals about possible sources of heterogeneity rather than explanatory evidence for individual-level treatment-effect modification.

**Table 2:** Subgroup analyses of potential moderators of the effect of exercise on cfPWV.

Group	Studies	cfPWV(m/s)			
	Number	WMD (95% CI)	I² (%)	*P* for heterogeneity	*P* for subgroup difference
Intensity					
Light	3	−0.36 (−0.67,−0.06)	5.6	0.347	0.341
Moderate	19	−0.81 (−1.26,−0.36)	84.1	<0.001	
Moderate to vigorous	15	−0.45 (−0.71,−0.18)	14.5	0.291	
Vigorous	22	−0.56 (−0.82,−0.30)	48.1	0.007	
Near-maximal	8	−1.28 (−2.56, 0.01)	97	<0.001	
Supramaximal	1	−1.86 (−4.08, 0.36)	0	<0.001	
Session duration (min/session)					
≤30	19	−0.97 (−1.35, −0.59)	78.5	<0.001	0.291
31–45	12	−0.51 (−0.87, −0.14)	44.2	0.044	
>45	24	−0.72 (−1.36, −0.07)	95.2	<0.001	
NA	13	−0.49 (−0.95, −0.04)	59.7	0.003	
Population type					
Healthy population	19	−0.65 (−0.91,−0.39)	50.3	0.007	0.717
Population with cardiometabolic risk	49	−0.73 (−1.10,−0.37)	91.6	<0.001	
Sex					
Man(only)	10	−0.78 (−1.04,−0.51)	29.8	0.171	0
Woman(only)	12	−0.83 (−1.23,−0.44)	84.6	<0.001	
Both sexes	42	−0.70 (−1.20,−0.19)	91.7	<0.001	
NA	4	0.00 (−0.28, 0.29)	0	0.653	
Age(years)					
<40	16	−0.54 (−0.77, −0.31)	54.2	0.005	0.273
40–59	39	−0.77 (−1.28, −0.26)	92.2	<0.001	
≥60	13	−0.91 (−1.34, −0.49)	70.6	<0.001	
Duration (week)					
≤8	25	−0.59 (−0.92, −0.27)	80.3	<0.001	0.644
8–12	27	−0.86 (−1.44, −0.28)	94.1	<0.001	
>12	16	−0.79 (−1.22, −0.36)	61.1	<0.001	
Measurement principle					
Applanation tonometry	36	−0.76 (−1.21, −0.31)	93.8	<0.001	0.205
Mechanotransducer	12	−0.55 (−0.78, −0.33)	0	0.819	
Oscillometric/Plethysmography	12	−0.77 (−1.07, −0.47)	47.9	0.032	
Doppler ultrasound	5	0.05 (−0.64, 0.75)	0	0.81	
Pressure transducer	3	−0.87 (−1.45, −0.29)	0	0.956	

cfPWV, carotid-femoral pulse wave velocity; WMD, weighted mean difference; I², inconsistency statistic; *P* for subgroup difference, *p* value for the test of between-subgroup differences.

**Table 3:** Univariable meta-regression analyses of continuous moderators of the effect of exercise on cfPWV.

Moderator	*n*	Coefficient	95% CI	*P*	I²res (%)	R² (%)
Mean age (years)	68	−0.0058415	(−0.02, 0.01)	0.414	80.55	1.06
Baseline cfPWV (m/s)	65	−0.181542	(−0.28, −0.08)	0.001	73.26	33.71
BMI (kg/m²)	67	0.0450215	(−0.01, 0.10)	0.081	80.36	5.9
Session duration (min/session)	55	0.0075121	(−0.01, 0.02)	0.376	82.39	0
Intervention duration (weeks)	68	−0.0309387	(−0.06, 0.00)	0.025	79.6	10.56

cfPWV, carotid-femoral pulse wave velocity; BMI, body mass index; I²res, residual heterogeneity after meta-regression; R², proportion of between-study heterogeneity explained by the moderator.

**Table 4:** Multivariable meta-regression analyses of baseline cfPWV, BMI, and intervention duration across different estimation methods.

Model	I²res (%)	R² (%)	Baseline cfPWV beta (95% CI)	Baseline cfPWV P	BMI beta (95% CI)	BMI P	Intervention duration beta (95% CI)	Intervention duration P
DerSimonian–Laird	51.21	86.71	−0.242 (−0.32, −0.17)	<0.001	0.083 (0.05, 0.12)	<0.001	−0.020 (−0.04, 0.00)	0.064
DerSimonian–Laird + Knapp–Hartung	51.21	86.71	−0.242 (−0.32, −0.16)	<0.001	0.083 (0.04, 0.12)	<0.001	−0.020 (−0.04, 0.00)	0.091
REML	58.17	65.46	−0.231 (−0.31, −0.15)	<0.001	0.082 (0.04, 0.12)	<0.001	−0.020 (−0.04, 0.00)	0.075

cfPWV, carotid-femoral pulse wave velocity; BMI, body mass index; REML, restricted maximum likelihood; I²res, residual heterogeneity after meta-regression; R², proportion of between-study heterogeneity explained by the moderator.

### 3.5 Results of network meta-analysis

**Figure 2:**
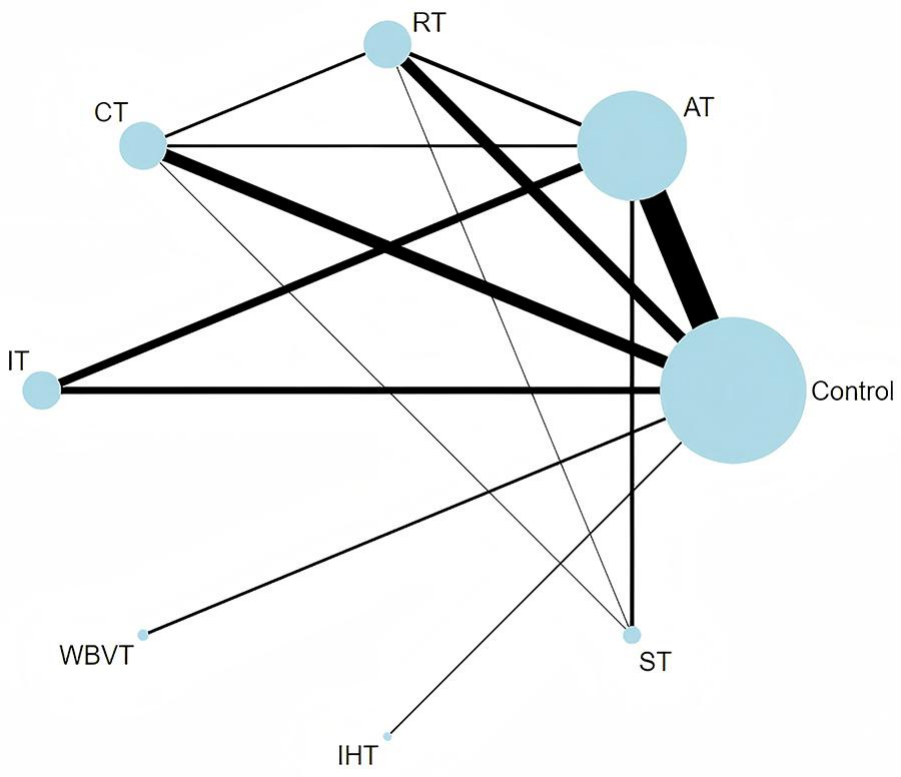
Network plot of eligible exercise modalities for carotid-femoral pulse wave velocity (cfPWV). Node size represents sample size, and edge thickness represents the number of direct comparisons.

The network of eligible exercise comparisons is shown in Figure 2. Global inconsistency was detected in the original network. Node-splitting analyses did not identify statistically significant direct-indirect disagreement for individual comparisons, whereas loop-specific analysis indicated inconsistency in the RT-CT-ST loop. In the sensitivity analysis excluding Liu and Zhu (19), inconsistency diagnostics were no longer statistically significant; this analysis was treated as diagnostic rather than replacing the primary full-network analysis.

**Figure 3:**
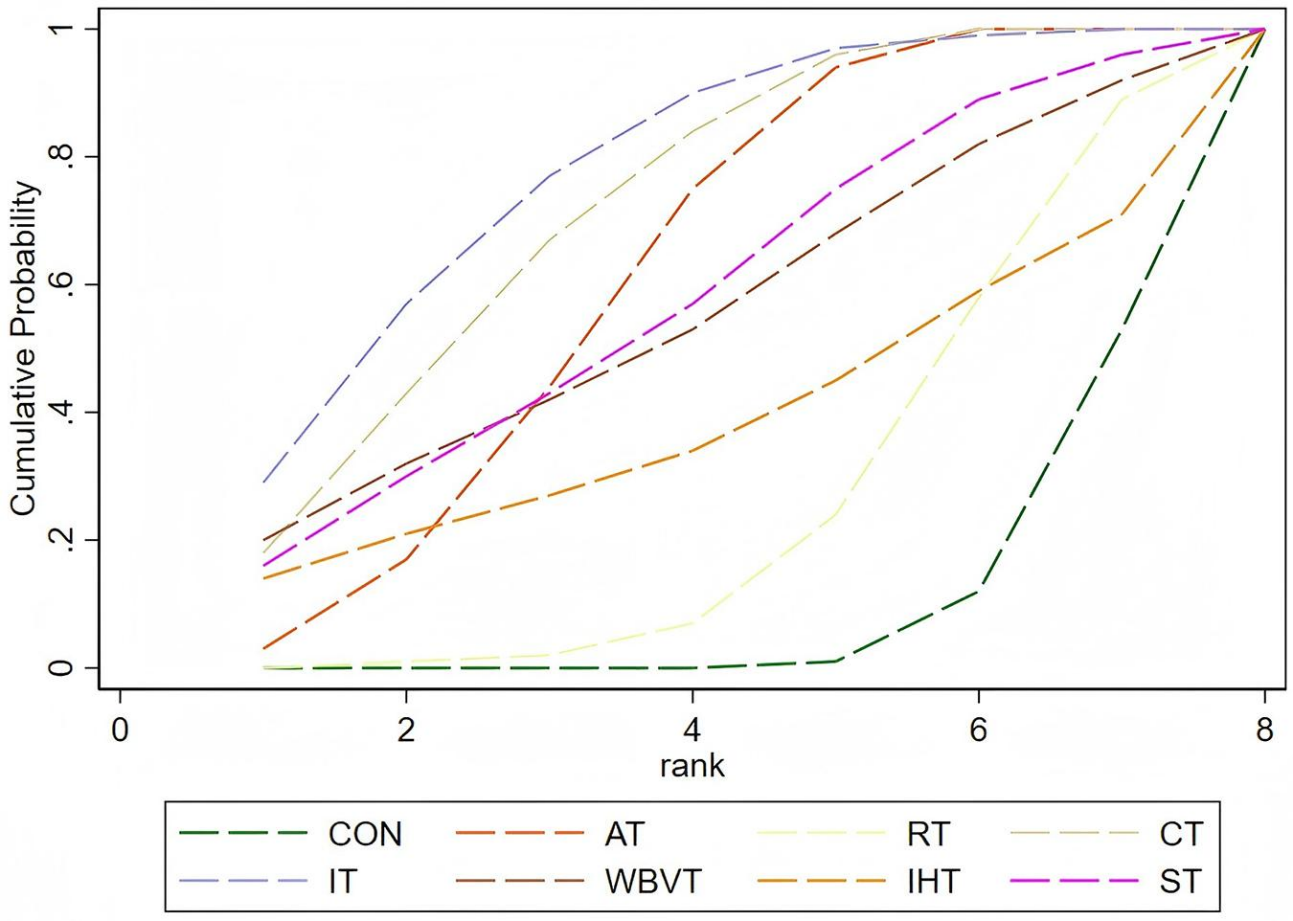
SUCRA ranking curves for exercise modalities. Rankings are descriptive and should not be interpreted as evidence of superiority.

Table 1 presents the direct pairwise estimates in the upper-right triangle and the network estimates in the lower-left triangle. In the primary network meta-analysis, aerobic training (WMD = −0.74, 95% CI: −1.07 to −0.40), combined training (WMD = −0.86, 95% CI: −1.35 to −0.37), and interval training (WMD = −0.94, 95% CI: −1.46 to −0.41) reduced cfPWV vs. non-exercise control. Resistance training, whole-body vibration training, isometric handgrip training, and stretching training did not show statistically significant effects vs. control, and no active exercise modality was statistically superior to another. Certainty of evidence vs. control was moderate for combined training, low for aerobic training, and very low for interval training. Although interval training had the highest SUCRA value, followed by combined and aerobic training, rankings should not be interpreted as evidence of superiority because active-vs.-active differences were non-significant and prediction intervals crossed the null. The cumulative ranking curves for the exercise modalities are shown in Figure 3.

In the Liu-excluded sensitivity network, aerobic, combined, and interval training retained statistically significant effects vs. control, whereas resistance training became statistically significant; no active-vs.-active comparison was statistically significant. Sensitivity league tables and rankings are provided in Supplementary Appendices 10, 12.

Evidence for whole-body vibration training, isometric handgrip training, and stretching training was sparse and imprecise; these modalities were therefore interpreted conservatively.

**Figure 4:**
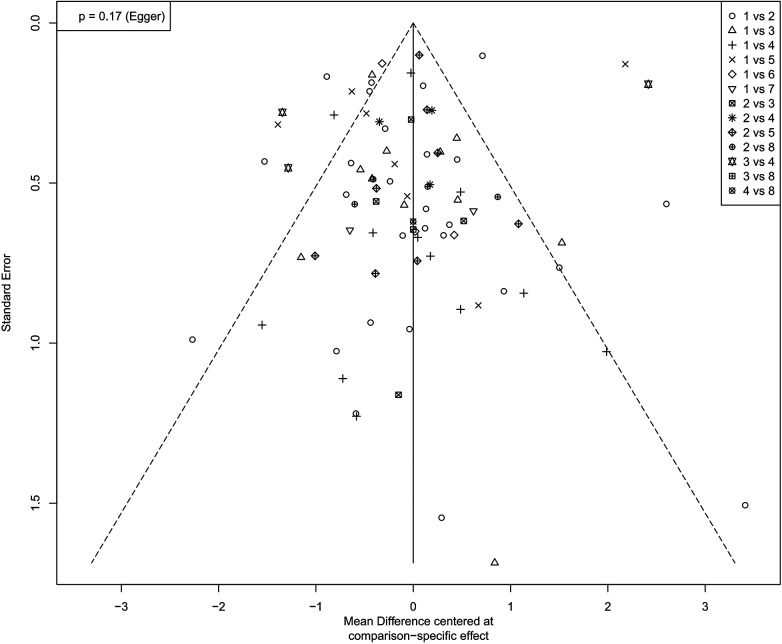
Comparison-adjusted funnel plot for the network meta-analysis. (1) Control; (2) Aerobic training; (3) Resistance training; (4) Combined training; (5) interval training; (6) WBVT; (7) isometric handgrip training; (8) Stretching training.

Visual inspection of the comparison-adjusted funnel plot (Figure 4) suggested acceptable symmetry, and Egger's test was not statistically significant (*P* = 0.17), indicating no clear evidence of publication bias or small-study effects in the network meta-analysis.

### 3.6 Dose-response results

The quadratic random-effects model provided the most parsimonious fit among the candidate dose-response functions. Key network assumptions, model-selection diagnostics, and model fit plots are reported in Supplementary Appendices 13, 14.

**Figure 5:**
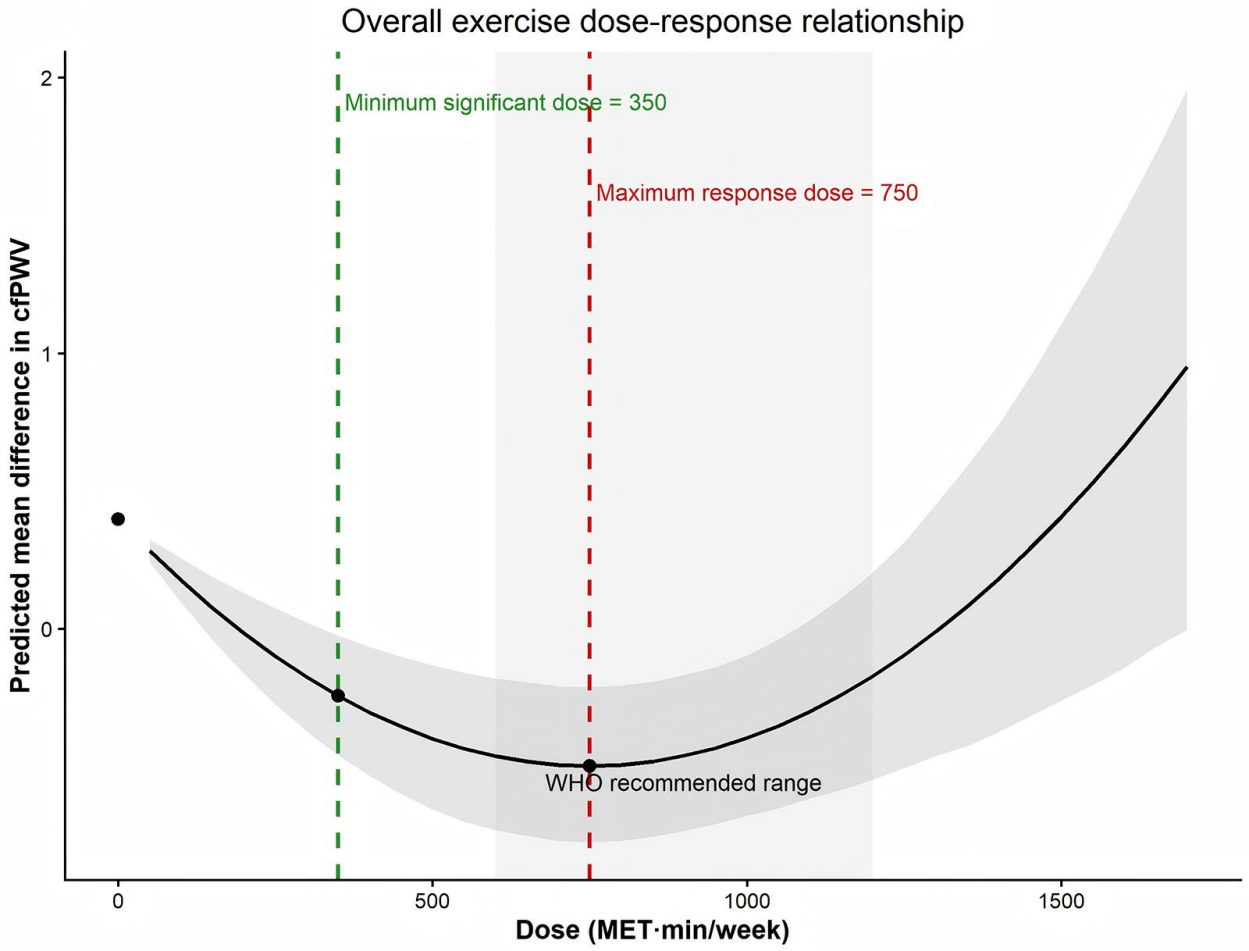
Overall Bayesian dose-response model for exercise dose and cfPWV. Shaded areas represent 95% credible intervals.

Bayesian dose-response modelling suggested a nonlinear association between weekly exercise dose and cfPWV (Figure 5). In the overall model, the fitted 95% credible interval first excluded the null at approximately 350 MET·min/week, and the most favourable fitted estimate occurred around 750 MET·min/week. These values should be interpreted as model-derived estimates rather than clinical dose thresholds. Uncertainty was greater at the lower and higher ends of the observed dose distribution, where data were sparse.

**Figure 6:**
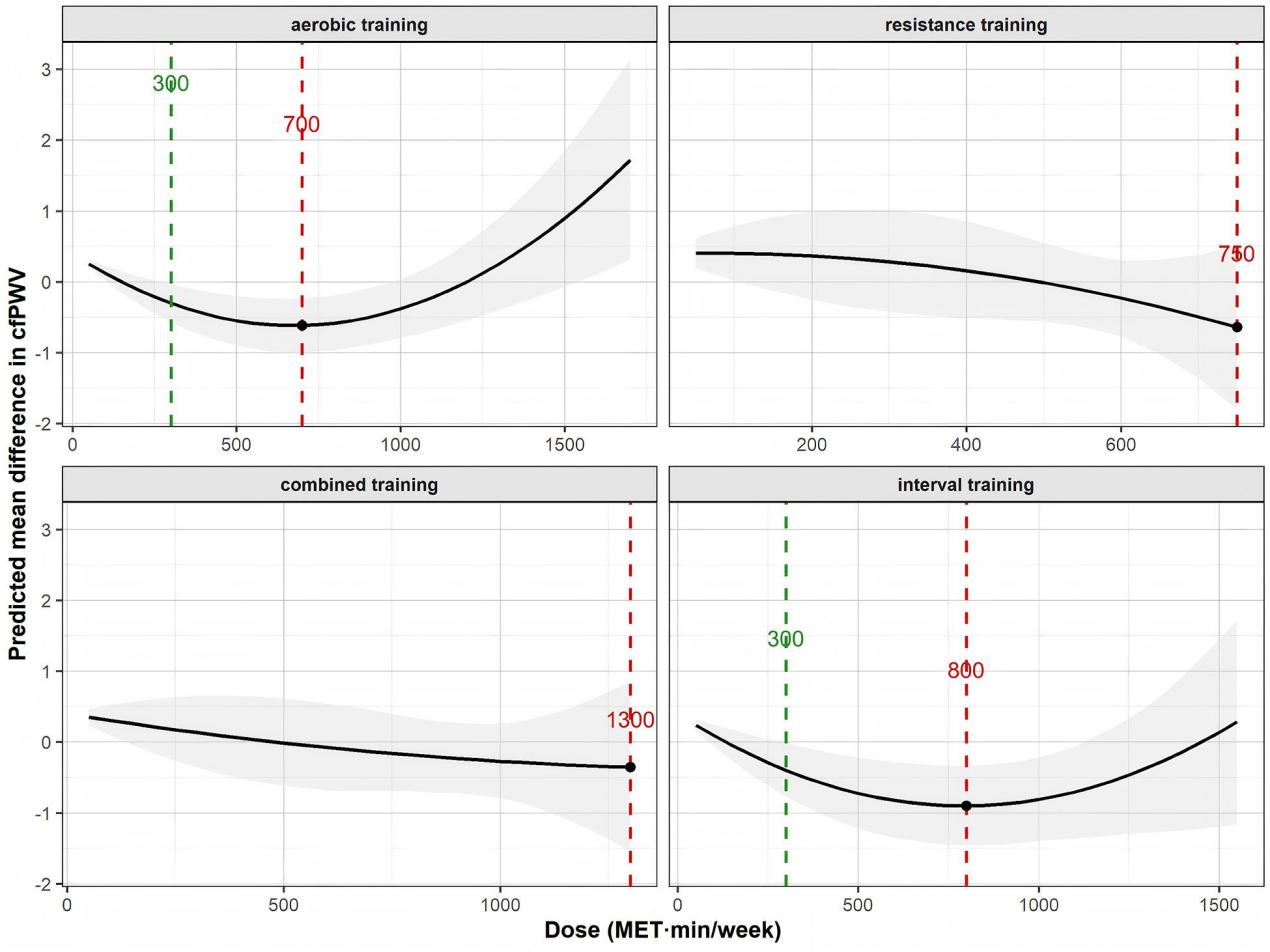
Modality-specific Bayesian dose-response curves for cfPWV. Curves are exploratory model-derived estimates.

Modality-specific curves suggested potentially different dose-response patterns across exercise types (Figure 6). Aerobic and interval training showed the clearest nonlinear fitted patterns, with the most favourable model-derived estimates occurring around 700–800 MET·min/week. Resistance and combined training showed more gradual fitted changes, whereas whole-body vibration training, isometric handgrip training, and stretching training produced unstable curves with wide credible intervals. These modality-specific estimates are exploratory and should not be translated directly into modality-specific prescription targets.

Exploratory rankings of modality-dose combinations are provided in Supplementary Appendix 16 and are not interpreted in the main text because they are model-derived and particularly sensitive to sparse data.

### 3.7 GRADE assessment

According to the GRADE system, the certainty of network evidence across comparisons with direct evidence ranged from moderate to very low (Supplementary Appendix 17). For the primary comparisons vs. non-exercise control, certainty was moderate for combined training, low for aerobic training, and very low for interval training. Downgrading was mainly attributable to imprecision, inconsistency, and study limitations.

## 4 Discussion

### 4.1 Discussion of the pairwise meta-analysis

The pairwise meta-analysis indicated that exercise was associated with lower cfPWV overall, with significant direct effects observed for aerobic training, combined training, and whole-body vibration training. This pattern is broadly consistent with previous evidence that aerobic-based and combined exercise can improve arterial stiffness in adults and populations at cardiovascular risk (100, 101). The overall reduction may be clinically relevant because cfPWV is an established prognostic marker of cardiovascular risk (1, 2), but the pooled change should not be translated directly into a proportional reduction in cardiovascular events.

Aerobic-based exercise may improve arterial stiffness through repeated shear-stress stimuli, enhanced endothelial function, blood-pressure changes, autonomic regulation, and inflammatory or oxidative pathways (72, 102). Interval training may evoke similar adaptations through higher intermittent haemodynamic stimuli, whereas resistance-training effects may depend on loading characteristics, rest intervals, breathing strategy, and participant characteristics (5, 6, 39). The significant estimate for whole-body vibration training was based on limited evidence and should not be generalized beyond the available trials (85).

Substantial heterogeneity remains central to interpretation. Large individual effects in several trials were rechecked and retained because they met the prespecified eligibility criteria, but their magnitude contributed materially to between-study variability. Sensitivity analyses indicated that the overall direction was not driven by a single influential study, although residual heterogeneity remained. The pooled estimate should therefore be interpreted as an average effect across heterogeneous settings rather than a uniform effect expected in all populations or programmes.

The moderator analyses provide exploratory context for this heterogeneity. Higher baseline cfPWV, BMI, and sex-related differences may be relevant to exercise responsiveness, but these findings were based on aggregate study-level characteristics and should not be interpreted as individual-level treatment-effect modifiers.

### 4.2 Discussion of the network meta-analysis

The network meta-analysis suggested that aerobic, combined, and interval training reduced cfPWV compared with non-exercise control, but the certainty of evidence differed across modalities. Evidence was of moderate certainty for combined training, low certainty for aerobic training, and very low certainty for interval training. Therefore, although interval training showed a statistically significant network estimate and the highest SUCRA value, this finding should be interpreted less confidently than the evidence for combined training. The current network does not establish a single preferred exercise modality because active-vs.-active comparisons were non-significant, direct evidence was sparse for several contrasts, and prediction intervals crossed the null.

Exercise prescription should therefore be individualized according to clinical status, safety, baseline fitness, musculoskeletal limitations, access to supervision, anticipated adherence, and participant preference rather than SUCRA values alone (3, 8).

The initial global inconsistency also requires caution. Although inconsistency was attenuated in the sensitivity analysis excluding Liu and Zhu (19), this result is best regarded as a diagnostic finding rather than proof that the original network fully satisfied the transitivity assumption. Differences in baseline cfPWV, clinical characteristics, and intervention composition may have contributed to inconsistency across the original network (21, 24).

### 4.3 Discussion of the dose-response analysis

The dose-response analysis should be interpreted as a modelling exercise rather than as a basis for clinical prescription. The fitted curves suggested that cfPWV reductions may occur within a moderate weekly dose range and that the most favourable model-derived estimates for aerobic and interval training clustered around 700–800 MET min/week. However, these estimates depend on the Bayesian model specification, MET assignment rules, and the distribution of available study arms. They are also less reliable at dose ranges with sparse observations. Accordingly, the present dose-response findings should be viewed as hypothesis-generating and should inform the design of future dose-ranging trials rather than immediate clinical dose recommendations.

### 4.4 Strengths and limitations

This study has several strengths. First, it focused specifically on cfPWV, the current gold-standard indicator of central arterial stiffness, improving the clinical relevance and comparability of the outcome assessment. Second, by combining pairwise meta-analysis, frequentist network meta-analysis, and Bayesian dose-response network meta-analysis, this study evaluated overall and direct effects, comparative modality patterns, and dose-response associations. Third, the review was based on a prospectively registered protocol, a comprehensive search strategy, and rigorous procedures for study selection, risk-of-bias assessment, and evidence appraisal. The inclusion of 69 randomized controlled trials involving 3,422 participants also provided a broad evidence base for the present synthesis.

Several limitations should be acknowledged. The included trials covered heterogeneous populations and intervention protocols, which may have challenged transitivity and limited the precision of modality-specific conclusions. Substantial residual heterogeneity persisted despite sensitivity, subgroup, and meta-regression analyses; therefore, moderator findings should be considered exploratory. The original network showed global inconsistency, and prediction intervals crossed the null for the principal modality-vs.-control comparisons. Dose-response estimates were approximate because MET·min/week required assumptions about exercise intensity and session structure, particularly for resistance and combined training. Finally, comparison-level GRADE certainty ranged from moderate to very low, mainly because of imprecision, inconsistency, and study limitations.

## 5 Conclusion

In this systematic review and network meta-analysis of randomized controlled trials, exercise training was associated with lower cfPWV relative to non-exercise control across heterogeneous adult populations. Aerobic, combined, and interval training reduced cfPWV vs. non-exercise control; however, no active exercise modality was statistically superior to another. Considering the GRADE assessments, the evidence was most consistent for combined training, whereas findings for interval training should be interpreted with greater caution.

The nonlinear dose-response analyses suggested model-derived associations between weekly exercise dose and cfPWV, with the most favourable fitted estimates clustering around moderate weekly doses for aerobic and interval training. These estimates should be regarded as hypothesis-generating rather than prescriptive dose targets because of substantial between-study heterogeneity, sparse data at the extremes of the dose distribution, and uncertainty in dose estimation for resistance-based and combined programmes. Future adequately powered trials with standardized exercise prescriptions, clearer reporting of medication use and adherence, and direct head-to-head comparisons are needed to determine whether specific exercise modalities or dose ranges provide clinically reproducible benefits for arterial stiffness.

## Data Availability

The original contributions presented in the study are included in the article/Supplementary Material, further inquiries can be directed to the corresponding author.
