# Supplementary material for: Comparative effectiveness of exercise modalities and doses on carotid-femoral pulse wave velocity in adults: a systematic review and network meta-analysis of randomized controlled trials
Source: Front Cardiovasc Med. 2026 Jul 28;13:1860243. doi: 10.3389/fcvm.2026.1860243 (PMC13457031; doi:10.3389/fcvm.2026.1860243)
Supplement: Supplementary file 1 [file Datasheet1.pdf]

Comparative effectiveness of exercise modalities and doses on carotid-femoral pulse wave velocity in adults: a systematic review, network meta-analysis of randomized controlled trials

Yanli Tan[1], Shiqi Liu[1], Liuhong Zang[1*]

[1]Xinjiang Normal University, Urumqi, Xinjiang 830017, China

**Correspondence:* zlh751225@126.com*

Appendix 1. Search strategy

Database	Search condition			
	Exercise	Adult	Arterial stiffness	Randomized controlled trials
PubMed/MEDLINE [Title/Abstract]	Exercise [Mesh] OR Exercise Therapy [Mesh] OR Sports [Mesh] OR Physical Exertion [Mesh] OR Resistance Training [Mesh] OR High-Intensity Interval Training [Mesh] OR exercise* OR training OR physical activity OR aerobic OR resistance OR strength OR isometric OR weight-bearing OR interval training OR HIIT OR walking OR running OR cycling OR yoga OR Tai Chi OR pilates	Adult[Mesh] OR Middle Aged [Mesh] OR Aged [Mesh] OR Young Adult [Mesh] OR adult* OR elderly OR older OR senior* OR men OR women OR postmenopausal	Vascular Stiffness[Mesh] OR Pulse Wave Analysis [Mesh] OR arterial stiffness OR vascular stiffness OR pulse wave velocity OR PWV OR baPWV OR cfPWV OR brachial-ankle pulse wave velocity OR carotid-femoral pulse wave velocity OR arterial compliance OR arterial elasticity OR beta stiffness OR stiffness index	Randomized Controlled Trial [Publication Type] OR randomized OR placebo OR randomly OR trial OR groups
Cochrane [Title/Abstract/keywords]	[mh "Exercise"] OR [mh "Exercise Therapy"] OR [mh "Sports"] OR [mh "Physical Exertion"] OR [mh "Resistance Training"] OR [mh "High-Intensity Interval Training"] OR exercise* OR training OR "physical activity" OR aerobic OR resistance OR strength OR isometric OR "weight bearing" OR "interval training" OR HIIT OR walking OR running OR cycling OR yoga OR "Tai Chi" OR pilates	[mh "Adult"] OR [mh "Middle Aged"] OR [mh "Aged"] OR [mh "Young Adult"] OR adult* OR elderly OR older OR senior* OR men OR women OR postmenopausal	[mh "Vascular Stiffness"] OR [mh "Pulse Wave Analysis"] OR "arterial stiffness" OR "vascular stiffness" OR "pulse wave velocity" OR "PWV" OR "baPWV" OR "cfPWV" OR "brachial-ankle pulse wave velocity" OR "carotid-femoral pulse wave velocity" OR "arterial compliance" OR "arterial elasticity" OR "beta stiffness" OR "stiffness index"	randomized controlled trial OR randomized OR placebo OR RCT
Embase [Title/Abstract/keywords]	'exercise'/exp OR 'exercise' OR 'kinesiotherapy'/exp OR 'kinesiotherapy' OR 'sport'/exp OR 'sport' OR 'resistance training'/exp OR 'resistance training' OR 'high intensity interval training'/exp OR 'high intensity interval training' OR 'aerobic exercise'/exp OR 'aerobic exercise' OR 'isometric exercise'/exp OR 'isometric exercise' OR exercise* OR training OR 'physical activity' OR aerobic OR resistance OR strength OR isometric OR 'weight bearing' OR 'interval training' OR 'hiit' OR walking OR running OR cycling OR yoga OR 'tai chi' OR pilates	'adult'/exp OR 'middle aged'/exp OR 'aged'/exp OR adult* OR elderly OR older OR senior* OR men OR women OR postmenopausal	'arterial stiffness'/exp OR 'pulse wave velocity'/exp OR 'vascular stiffness'/exp OR 'arterial compliance'/exp OR 'arterial stiffness' OR 'vascular stiffness' OR 'pulse wave velocity' OR 'pwv' OR 'bapwv' OR 'cfpwv' OR 'brachial ankle pulse wave velocity' OR 'carotid femoral pulse wave velocity' OR 'arterial compliance' OR 'arterial elasticity' OR 'beta stiffness' OR 'stiffness index'	'randomized controlled trial'/exp OR 'controlled clinical trial'/exp OR randomized OR placebo OR randomly OR trial OR groups
Web of Science [Topic]	"Exercise" OR "Exercise Therapy" OR "Sports" OR "Physical Exertion" OR "Resistance Training" OR "High-Intensity Interval Training" OR exercis* OR training OR "physical activity" OR aerobic OR resistance OR strength OR isometric OR "weight bearing" OR "interval training" OR "HIIT" OR walking OR running OR cycling OR yoga OR "Tai Chi" OR pilates	"Adult" OR "Middle Aged" OR "Aged" OR "Young Adult" OR adult* OR elderly OR older OR senior* OR men OR women OR postmenopausal	"Vascular Stiffness" OR "Pulse Wave Analysis" OR "arterial stiffness" OR "vascular stiffness" OR "pulse wave velocity" OR "PWV" OR "baPWV" OR "cfPWV" OR "brachial-ankle pulse wave velocity" OR "carotid -femoral pulse wave velocity" OR "arterial compliance" OR "arterial elasticity" OR "beta stiffness" OR "stiffness index"	"Randomized Controlled Trial" OR randomized OR placebo OR randomly OR trial OR groups
EBSCO [Abstract]	exercise* OR training OR "physical activity" OR aerobic OR resistance OR strength OR isometric OR "weight bearing" OR "interval training" OR HIIT OR walking OR running OR cycling OR yoga OR "Tai Chi" OR pilates OR sport*) OR AB (exercise* OR training OR "physical activity" OR aerobic OR resistance OR strength OR isometric OR "weight bearing" OR "interval training" OR HIIT OR walking OR running OR cycling OR yoga OR "Tai Chi" OR pilates OR sport*	adult* OR "middle aged" OR aged OR elderly OR older OR senior* OR men OR women OR postmenopausal) OR AB (adult* OR "middle aged" OR aged OR elderly OR older OR senior* OR men OR women OR postmenopausal	"arterial stiffness" OR "vascular stiffness" OR "pulse wave velocity" OR PWV OR baPWV OR cfPWV OR "brachial-ankle pulse wave velocity" OR "carotid -femoral pulse wave velocity" OR "arterial compliance" OR "arterial elasticity" OR "beta stiffness" OR "stiffness index") OR AB ("arterial stiffness" OR "vascular stiffness" OR "pulse wave velocity" OR PWV OR baPWV OR cfPWV OR "brachial-ankle pulse wave velocity" OR "carotid-femoral pulse wave velocity" OR "arterial compliance" OR "arterial elasticity" OR "beta stiffness" OR "stiffness index"	"randomized controlled trial" OR randomized OR placebo OR randomly OR trial OR groups) OR AB ("randomized controlled trial" OR randomized OR placebo OR randomly OR trial OR groups
Scopus [Title/Abstract/keywords]	exercise* OR training OR "physical activity" OR aerobic OR resistance OR strength OR isometric OR "weight bearing" OR "interval training" OR HIIT OR walking OR running OR cycling OR yoga OR "Tai Chi" OR pilates	adult* OR "middle aged" OR aged OR elderly OR older OR senior* OR men OR women OR postmenopausal	"arterial stiffness" OR "vascular stiffness" OR "pulse wave velocity" OR PWV OR baPWV OR cfPWV OR "brachial-ankle" OR "carotid-femoral" OR "arterial compliance" OR "arterial elasticity" OR "beta stiffness" OR "stiffness index"	"randomized controlled trial" OR randomized OR placebo OR randomly OR trial OR groups

Appendix 2. Characteristics of the included studies.

Author year	Country	Population characteristics				Outcome (cfPWV [m/s])		Characteristics of the intervention	
		Age (years)	Sample size (%males)	Type of population	BMI	Measuring device	Baseline	PE intervention	PE characteristics
Rugolo 2019(E. S. V. R. Oliveira et al. 2019)	Brazil	IG=50 ±17.2<br>CG=58 ±15.0	IG=15(46.7)<br>CG=15(53.3)	Hemodialysis Patients	IG=25.7±3.5<br>CG=26.7±4.6	SphygmoCor	IG=8.5 ±2.86<br>CG=10.3 ±3.80	Aerobic training	IG = Cycling<br>• 16 weeks<br>• 3 times/week<br>• 30 min/session<br>• 65%–75%HRmax
Greenwood 2015(b)(Greenwood, Koufaki, Mercer, Rush, et al. 2015)	United Kingdom	IG1=53.9 ±10.7<br>IG2=54.6±10.6<br>CG=49.5±10.6	IG1=13(80)<br>IG2=13(54)<br>CG=20(50)	Kidney transplant recipients	IG1=26.6 ± 4.7<br>IG2=28.2 ± 3.6<br>CG=27.3 ± 3.6	SphygmoCor	IG1=9.0±1.4<br>IG2=9. 1±1.8<br>CG=8.9 ± 2.3	Aerobic training	IG1 = Walking, jogging or cycling<br>• 12 weeks<br>• 3 times/week<br>• 60 min/session<br>• Vigorous (80%HRR)
								resistance training	IG2 = 8 major muscle groups, bodyweight and equipment<br>• 12 weeks<br>• 3 times/week<br>• 60 min/session<br>• Vigorous (80%1RM)
Chrysohoou 2015(Chrysohoou et al. 2015)	Greece	IG=63 ±9<br>CG=56 ±11	IG=33(88)<br>CG=39(72)	chronic heart failure patients(CHF)	IG=28.85 ± 4.2<br>CG=31.3 ± 7	SphygmoCor	IG=9.5 ±2.5<br>CG=8.8 ±1.3	Combined training	IG = HIIT(bike)+resistance training<br>• 12 weeks<br>• 3 times/week<br>• 45 min/session<br>• AT:80%–100% WRpea<br>• RT:30%-90%1RM
Taylor 2020(Taylor et al. 2022)	Australia	IG1=64 ± 8<br>IG2=63 ± 8	IG1=28(93)<br>IG2=26(93)	coronary artery disease(CAD)	IG1=28.7 ± 4.3<br>IG2=29.5 ± 4.2	SphygmoCor	IG1=8.3±1.5<br>IG2=8.5±1.5	High-Intensity Interval Training(HIIT)	IG1 =bike/run<br>• 4 weeks<br>• 3 times/week<br>• 40 min/session<br>• Vigorous (85-95 %HRpeak)
								Aerobic training	IG2 = bike/run<br>• 4 weeks<br>• 3 times/week<br>• 40 min/session<br>• Moderate (65-75%HRpeak)
Greenwood 2015(a)(Greenwood, Koufaki, Mercer, MacLaughlin, et al. 2015)	United Kingdom	IG=53.8 ±13.5<br>CG=53.3 ±12.9	IG=8(75)<br>CG=10(90)	chronic kidney disease (CKD)	IG=27.40 ± 3.52<br>CG=28.44 ± 4.24	Vicorder	IG=9.5 ±2.2<br>CG=8. 1 ±2.4	Combined training	IG = Aerobic + Resistance<br>• 52 weeks<br>• 3 times/week<br>• 40 min/session<br>• Vigorous (80%HRR)
Kim 2021(Kim et al. 2021)	South Korea	IG=20.5 ± 2.78<br>CG=20±2.83	IG=16(NA)<br>CG=8(NA)	Healthy	IG=21.6 ± 2.43<br>CG=22.4±2.55	SphygmoCor	IG=5.23±0.33<br>CG=5.40 ± 0.45	resistance training	IG = Resistance Training<br>• 8 weeks<br>• 2 times/week<br>• 55 min/session<br>• Vigorous (70%–80% 1RM)
Liu 2025(Liu and Zhu 2025)	China	IG1= 62.35 ± 8.45<br>IG2=64.39 ± 7.66	IG1=35(51.4)<br>IG2=35(54.3)	coronary heart disease (CHD)	NA	Boso ABI-100	IG1= 12.09 ± 0.56<br>IG2=12.15 ± 0.45	Combined training	IG1 =Aerobic(table tennis) + Resistance<br>• 17 weeks<br>• NA<br>• NA<br>• Moderate-to-high intensity
								resistance training	IG2 = Resistance training<br>• 17 weeks<br>• 7 times/week<br>• 30 min/session<br>• Moderate-to-high intensity

Lee 2015(Y. H. Lee et al. 2015)	South Korea	IG= 64 ± 7.40<br>CG=63 ± 5.45	IG=14(64.3)<br>CG=12(58.3)	Chronic poststroke hemiparesis patients	IG= 31.3 ± 4.2<br>CG=31. 1 ± 4.7	SphygmoCor	IG=10.50 ± 1.93<br>CG=9.85 ± 1.98	Combined training	IG = Aerobic + Resistance training<br>• 16 weeks<br>• 3 times/week<br>• 60 min/session<br>• Moderate (50%-70%HRR)
Holloway 2016(Holloway et al. 2018)	United Kingdom	IG= 21 ± 2<br>CG=21 ± 2	IG=12(100)<br>CG=9(100)	Healthy	IG= 24±3<br>CG=23±3	SphygmoCor	IG=6 ± 1<br>CG=5 ± 1	High-Intensity Interval Training(HIIT)	IG = bike<br>• 6 weeks<br>• 3 times/week<br>• 15 min/session<br>• Vigorous (90% VO_2max)
Jeong 2023(Jeong et al. 2023)	America	IG1= 61.7 ± 8.5<br>IG2=65.4 ± 8.5	IG1=32(81)<br>IG2=26(80)	chronic kidney disease (CKD)	IG1= 32.6 ± 6.3<br>IG2=31.9 ± 3.4	SphygmoCor	IG1= 8.6 ± 2.8<br>IG2=9.4 ± 1.3	Aerobic training	IG1 =bike<br>• 12 weeks<br>• 3 times/week<br>• 20-45 min/session<br>• Moderate (50%-85%HRR)
								Stretching training	IG2 =Stretching and balance<br>• 12 weeks<br>• 3 times/week<br>• 20-45 min/session<br>• Light (below 50%HRR)
Koh 2010(Koh et al. 2010)	Australia	IG1=52.3 ± 10.9<br>IG2=52. 1 ±13.6<br>CG=51.3 ± 14.4	IG1=15(66.7)<br>IG2=15(73.3)<br>CG=16(50)	chronic kidney disease (CKD)	IG1=27.6 ± 7.2<br>IG2=27.9 ± 4.9<br>CG=28.6 ± 7.3	SphygmoCor	IG1=9. 1 ± 2.8<br>IG2=9.7 ± 3.2<br>CG=8.7 ± 2.5	Aerobic training	IG1 =Cycling<br>• 24 weeks<br>• 3 times/week<br>• 30 min/session<br>• Moderate (RPE 12–13)
								Aerobic training	IG2 =Walking<br>• 24 weeks<br>• 3 times/week<br>• 30 min/session<br>• Moderate (RPE 12–13)
Heydari 2013(Heydari et al. 2013)	Australia	24.9 ± 4.3	IG=20(100)<br>CG=18(100)	physically inactive	IG= 28.4 ± 2.4<br>CG=29 ± 3.9	SphygmoCor	IG= 6.30 ± 0.96<br>CG=5.80 ± 0.84	High-Intensity Interval Training(HIIT)	IG = Cycling<br>• 12 weeks<br>• 3 times/week<br>• 20 min/session<br>• Vigorous (80-90% HRmax)
Thompson 2022(Thompson et al. 2022)	Canada	IG= 67.7±10.3<br>CG=65.0±18.2	IG=21(61.9)<br>CG=23(65.2)	chronic kidney disease (CKD)	IG=30.3±5.6<br>CG=30.7±7.9	Complior		Combined training	IG = Aerobic + Resistance training<br>• 8 weeks<br>• 3 times/week<br>• 50-60 min/session<br>• Moderate (40%-59%HRR)
Williams 2021(Williams et al. 2021)	Canada	IG= 45.8±13.6<br>CG=45.6±10.5	IG=14(64.3)<br>CG=14(57.1)	Individuals with chronic Spinal Cord Injury (SCI)	NA	SPT-301	IG= 10.6 ± 2.9<br>CG=8.0 ± 2.0	Combined training	IG = Aerobic + Resistance training<br>• 8 weeks<br>• 5 times/week<br>• NA<br>• Moderate-to-vigorous
Kobayashi 2022(Kobayashi et al. 2022)	Japan	IG1=62.5 ± 2.8<br>IG2=62.5 ± 2.8<br>CG=60.9 ± 3.9	IG1=15(0)<br>IG2=15(0)<br>CG=15(0)	Postmenopausal females	IG1=22.4 ± 1.6<br>IG2=22.8 ± 1.1<br>CG=22.7 ± 1.4	SphygmoCor	IG1=9.19 ± 0.22<br>IG2=9.26 ± 0.28<br>CG=9.12 ±0.26	Aerobic training	IG1 =Walking<br>• 8 weeks<br>• 2 times/week<br>• 30 min/session<br>• Moderate (65% HRmax)
								Aerobic training	IG2 =Walking<br>• 8 weeks<br>• 4 times/week<br>• 30 min/session<br>• Moderate (65% HRmax)

Cortez-Cooper 2008(Cortez-Cooper et al. 2008)	America	IG1=52 ± 7.2<br>IG2=51 ± 3.5<br>IG3=54 ± 6.9	IG1=13(23.1)<br>IG2=12(25)<br>IG3=12(50)	Healthy	IG1=26.8 ± 4.0<br>IG2=27.0 ± 3.8<br>IG3=25.7 ± 3.8	VP-2000	IG1=11.09±1.33<br>IG2=11.00 ± 0.90<br>IG3=11.33 ± 1.91	resistance training	IG1 =Strength training<br>• 13 weeks<br>• 3 times/week<br>• 30-45 min/session<br>• Vigorous (70% 1RM)
								Combined training	IG2 = Aerobic + Resistance training<br>• 13 weeks<br>• 4 times/week<br>• 30-45 min/session<br>• Vigorous (60%-75%HRR)
								Stretching training	IG3 = Stretching training<br>• 13 weeks<br>• 4 times/week<br>• 30-45 min/session<br>• light
Slivovskaja 2017(Slivovskaja et al. 2018)	Lithuania	IG=53.89 ± 6.43<br>CG=52.01 ± 7.67	IG=84(40.5)<br>CG=42(57.1)	Metabolic Syndrome	IG=30.89 ± 3.97<br>CG=30.97 ± 3.51	SphygmoCor	IG= 8.47 ± 1.40<br>CG=8.01 ± 1.06	Aerobic Training	IG = bike<br>• 8 weeks<br>• 5 times/week<br>• 30-40 min/session<br>• NA
Figueroa 2015(Figueroa et al. 2015)	America	IG=57.00 ± 3.54<br>CG=58.00 ± 3.46	IG=24(NA)<br>CG=12(NA)	Obese Postmenopausal Women	IG=34.2±4.2<br>CG=32.2±7.27	VP-2000	IG= 12.40 ± 1.71<br>CG=12.10 ± 2.08	Whole-body vibration training (WBVT)	IG = leg exercises<br>• 12 weeks<br>• 3 times/week<br>• 10-30 min/session<br>• NA
Lin 2021(Lin et al. 2021)	China	IG1=66 ±7.21<br>IG2=69 ± 7.62<br>CG=70 ± 9.00	IG1=13(46.2)<br>IG2=12(50)<br>CG=9(44.4)	Healthy	IG1=23.8 ± 3.97<br>IG2=26.5 ± 5.54<br>CG=25.4 ± 3.60	Sonosite Ultrasound System	IG1=9.34 ± 2.29<br>IG2=8.39 ± 3.74<br>CG=8.96 ±3.05	Aerobic training	IG1 = walking<br>• 8 weeks<br>• 3 times/week<br>• 30 min/session<br>• Moderate (60-70% HRmax)
								Combined training	IG2 = Aerobic + Resistance training<br>• 8 weeks<br>• 3 times/week<br>• 30 min/session<br>• Moderate (60-70% HRmax)
Pascoalino 2014(Pascoalino et al. 2015)	Brazil	IG=45.0 ± 16.7<br>CG=45.0 ± 18.0	IG=31(74.2)<br>CG=9(55.6)	Heart Transplant Recipients	IG=26.7 ± 5.0<br>CG=25. 1 ± 7.5	Complior	IG=10.00 ± 1.89<br>CG=10.38 ± 2.28	Aerobic training	IG = walking/jogging<br>• 12 weeks<br>• 3 times/week<br>• 40 min/session<br>• Vigorous (70% VO_2max)
Farah 2018(Farah et al. 2018)	Brazil	IG=60. 12 ± 8.00<br>CG=58.00 ± 12.00	IG=32(30)<br>CG=16(31)	Hypertensive Patients	IG=30.58 ±5.62<br>CG=31.70 ± 5.20	SphygmoCor	IG=9.19 ± 1.98<br>CG=8.70 ± 2.00	Isometric handgrip Training(IHT)	IG = isometric contractions<br>• 12 weeks<br>• 3 times/week<br>• 12 min/session<br>• light 30% of maximal voluntary contraction
Omar 2023(Omar et al. 2023)	Malaysia	IG=26.17 ± 6.68<br>CG=26.62 ± 7.39	IG=36(100)<br>CG=34(100)	sedentary	IG=26.13 ± 5.99<br>CG=24.49 ± 4.54	Vicorder	IG=7.22 ± 0.83<br>CG=7.25 ± 0.64	Aerobic training	IG =Walking<br>• 12 weeks<br>• 5 times/week<br>• NA<br>• NA
Guimarães 2010(Guimarães et al. 2010)	Brazil	IG1=50±8<br>IG2=45±9<br>CG=47±6	IG1=16(43.8)<br>IG2=16(25)<br>CG=11(18.2)	hypertensive patients	IG1=28±4<br>IG2=29±5<br>CG=26±5	Complior	IG1=10. 15±1.66<br>IG2=9.44±0.91<br>CG= 10.23±1.82	Combined training	IG1 = Aerobic + Resistance training<br>• 16 weeks<br>• 3 times/week<br>• 60 min/session<br>• Vigorous (60%HRR)

								Combined training	IG2 = Aerobic + Resistance training<br>• 16 weeks<br>• 3 times/week<br>• 60 min/session<br>• Moderate/Vigorous (50%HRR alternate 80%HRmax)
Lai 2025(Lai et al. 2025)	China	IG=55.76 ± 14.11<br>CG=60.28 ± 8.93	IG=75(62.7)<br>CG=39(59)	Chronic hemodialysis (CHD) patients	IG=23.80 ± 4.38<br>CG=24.88 ± 3.78	SphygmoCor	IG=9.17 ± 2.67<br>CG=8.40 ± 2.67	Aerobic training	IG =Cycling<br>• 52 weeks<br>• 3 times/week<br>• 20 min/session<br>• light to Moderate (3-5.9 METs)
Croymans 2014(Croymans et al. 2014)	America	IG=21.50 ± 2.22<br>CG=21.87 ± 1.48	IG=28(100)<br>CG=8(100)	Healthy	IG=31.10 ± 2.22<br>CG=33.17 ± 2.59	SphygmoCor	IG=6.77 ± 0.89<br>CG=6.93 ± 0.67	Resistance training	IG =Resistance training<br>• 12 weeks<br>• 3 times/week<br>• 60 min/session<br>• Vigorous
DeVallance 2016(DeVallance et al. 2016)	America	IG=49.0 ± 12.0<br>CG=44.0 ± 10.4	IG=16(NA)<br>CG=12(NA)	Healthy	IG=24.0 ± 3.6<br>CG=24.0 ± 3.5	SphygmoCor	IG=6.90 ± 1.20<br>CG=6.80 ± 1.04	Resistance training	IG =Resistance training<br>• 8 weeks<br>• 3 times/week<br>• NA<br>• Progressive((60%-85% 1RM)
		IG=51.0 ± 10.8<br>CG=51.0 ± 16.0	IG=13(NA)<br>CG=16(NA)	Metabolic Syndrome	IG=32.0 ± 3.6<br>CG=33.0 ± 8.0	SphygmoCor	IG=7.90 ± 1.44<br>CG=7.90 ± 1.60	Resistance training	IG =Resistance training<br>• 8 weeks<br>• 3 times/week<br>• NA<br>• Progressive((60%-85% 1RM)
Gómez-Sánchez 2022(Gómez-Sánc hez et al. 2022)	Spain	IG=47.96 ± 9.95<br>CG=47.78 ± 9.62	IG=127(33.1)<br>CG=126(27.8)	Overweight/Obese sedentary adults	IG=32.71 ± 3.27<br>CG=33.01 ± 3.31	SphygmoCor	IG=7.42 ± 1.38<br>CG=7.64 ±1.60	Aerobic training	IG = walking<br>• 12 weeks<br>• NA<br>• NA<br>• NA
Okamoto 2012(Okamoto et al. 2013)	Japan	IG= 19.2 ± 0.69<br>CG=19.1 ± 0.6	IG=20(50)<br>CG=10(50)	Healthy sedentary young adults	IG=22.35 ± 3.35<br>CG=22.3 ± 3.7	PWV/ABI	IG=6.11 ± 1.35<br>CG=6.36 ± 1.29	Resistance training	IG =resistance training<br>• 10 weeks<br>• 2 times/week<br>• NA<br>• Moderate-vigorous (50-80%1RM)
Cox 2024(Cox et al. 2024)	Australia	IG1=59.0±8.8<br>IG2=60.1±7.3<br>CG=59.4±10.2	IG1=23(60.9)<br>IG2=23(60.9)<br>CG=23(60.9)	Type 2 Diabetes (T2D)	IG1=32.6±5.1<br>IG2=34.0±6.6<br>CG=33.9±6.4	SphygmoCor	IG1=8.7±1.6<br>IG2=9.2±1.3<br>CG=9.6±1.8	Combined training	IG1 = Aerobic + resistance<br>• 8 weeks<br>• 3 times/week<br>• 26 min/session<br>• AT:50-60% HRpeak<br>• RT:85-95% HRpeak
								Combined training	IG2 = Aerobic + resistance<br>• 8 weeks<br>• 4 times/week<br>• 52.5 min/session<br>• AT:50-60% HRpeak<br>• RT:Moderate（RPE 11-13）
Cooke 2018(Cooke et al. 2018)	Canada	IG=58.2 ± 17.2<br>CG=52.5 ± 15.4	IG=10(70)<br>CG=10(70)	chronic kidney disease (CKD) patients on hemodialysis	IG=25.6 ± 4.3<br>CG=27.2 ± 6.1	SphygmoCor	IG=8.6 ± 2.3<br>CG=8.33 ±1.48	Aerobic training	IG = pedaling<br>• 16 weeks<br>• 3 times/week<br>• 42.6 min/session<br>• Moderate to vigorous (RPE 12-16 on Borg 6-20 scale)
Van Craenenbroeck 2015(Van Craenenbroeck et al. 2015)	Belgium	IG=51.5 ± 11.8<br>CG=54.7 ± 14.1	IG=19(58)<br>CG=21(52)	chronic kidney disease (CKD)	IG=28.3 ± 6.2<br>CG=28.3 ± 5.8	SphygmoCor	IG=9.1 ±1.9<br>CG=8.8 ±1.6	Aerobic training	IG = cycling<br>• 12 weeks<br>• 7 times/week<br>• 40 min/session<br>• Moderate (90% HR_{AT})

Figueroa 2014(Figueroa et al. 2014)	America	IG=55.5 ± 2.5<br>CG=56.4 ± 3.5	IG=13(0)<br>CG=12(0)	Postmenopausal women with prehypertension and hypertension	IG=33.6 ± 4.7<br>CG=36.0 ± 3.1	VP-2000	IG=13.4 ±1.44<br>CG=12.5 ±1.39	Whole-body vibration training (WBVT)	IG = Squats/Calf raises<br>• 12 weeks<br>• 3 times/week<br>• NA<br>• 25–40 Hz (progressive)
Yoshizawa 2008(Yoshizawa et al. 2009)	Japan	IG1=47.0 ± 6.63<br>IG2=47.0 ± 6.93<br>CG=49.0 ± 10.39	IG1=11(0)<br>IG2=12(0)<br>CG=12(0)	Healthy sedentary middle-aged women	IG1=24.8 ± 3.32<br>IG2=24.6 ± 3.81<br>CG=21.8 ± 3.46	PWV/ABI	IG1=9.22 ± 1.66<br>IG2=9.20 ± 0.97<br>CG=8.88 ± 1.06	Resistance training	IG1 = variable resistance training equipment<br>• 12 weeks<br>• 2 times/week<br>• NA<br>• Moderate (60% 1RM)
								Aerobic training	IG2 = Cycling<br>• 12 weeks<br>• 2 times/week<br>• 30 min/session<br>• Moderate-Vigorous (60%-70% VO_2max)
Chang 2025(Chang et al. 2025)	Australia	IG=62.90 ± 7.63<br>CG=63.2 ± 8.5	IG=21(50)<br>CG=14(52)	Adults with Type 2 Diabetes (T2D)	IG=30.05 ± 5.58<br>CG=29.6 ± 4.1	SphygmoCor	IG=8.91 ± 1.63<br>CG=8.1 ± 1.9	Aerobic training	IG =Aerobic training<br>• 8 weeks<br>• 7 times/week<br>• 22 min/session<br>• Moderate
Correia 2020(A. Correia M et al. 2020)	Brazil	IG=66 ± 12<br>CG=67 ± 11	IG=43(66)<br>CG=41(62)	Peripheral Artery Disease (PAD) patients	IG=27 ± 5<br>CG=26 ± 7	SphygmoCor	IG=9.9 ±3.28<br>CG=9.6 ±3.20	Isometric handgrip Training(IHT)	IG = handgrip device<br>• 8 weeks<br>• 3 times/week<br>• 20 min/session<br>• light 30% of maximal voluntary contraction
Banks 2023(Banks et al. 2024)	America	IG=52 ± 6<br>CG=55 ± 6	IG=13(61.5)<br>CG=13(61.5)	Untreated elevated BP or stage-1 hypertension	IG=29.9 ±4.2<br>CG=28.0 ± 4.3	SphygmoCor	IG=7.0 ±0.92<br>CG=7.3 ±1.10	Resistance training	IG = Resistance training<br>• 9 weeks<br>• 3 times/week<br>• 40 min/session<br>• NA
Blumenthal 2021(Blumenthal et al. 2021)	America	IG=62 ± 9<br>CG=63 ± 9	IG=90(52)<br>CG=50(52)	Resistant Hypertension (RH)	IG=36 ± 6<br>CG=36 ± 5	Doppler ultrasound	IG=9.8 ± 4.2<br>CG=10.1 ± 2.3	Aerobic training	IG = Cycling/walking<br>• 16 weeks<br>• 3 times/week<br>• 45 min/session<br>• Vigorous (70-85%HRR)
Brubaker 2022(Brubaker et al. 2023)	America	IG1=67.9±5.4<br>IG2=69.7±5.8	IG1=38(16)<br>IG2=39(14)	Obese older HFpEF(heart failure with preserved ejection fraction.) patients	IG1=40.0±5.9<br>IG2=39.2±5.6	Doppler echocardiography	IG1=10.11±2.56<br>IG2=10.57±3.52	Aerobic training	IG1 = Cycling/walking<br>• 20 weeks<br>• 3 times/week<br>• 40 min/session<br>• Moderate-Vigorous (50-70%HRR)
								Combined training	IG2 = Aerobic + resistance training<br>• 20 weeks<br>• 3 times/week<br>• 60 min/session<br>• progressive (20-50%1RM)
Choo 2014(Choo et al. 2014)	Korea	IG1=42.2±9.5<br>IG2=46.0±10.0<br>IG3=41.8±6.6	IG1=50(0)<br>IG2=30(0)<br>IG3=30(0)	Women with abdominal obesity	IG1=28.5±0.54<br>IG2=27.9±0.58<br>IG3=29.1±0.79	SphygmoCor	IG1=7.77 ± 1.61<br>IG2=7.89 ± 1.21<br>IG3=7.99 ± 1.01	Aerobic training	IG1 = Treadmill/Bike<br>• 36 weeks<br>• 3 times/week<br>• 60 min/session<br>• Moderate-Vigorous (50-70%HRR)
								Resistance training	IG2 = Resistance training<br>• 36 weeks<br>• 3 times/week<br>• 60 min/session<br>• progressive (40-60%1RM)

								Combined training	IG3 = Aerobic + resistance training<br>• 36 weeks<br>• 3 times/week<br>• 60 min/session<br>• • Moderate-Vigorous (50-70%HRR)<br>• progressive (40-60%1RM)
Donley 2014(Donley et al. 2014)	America	IG=46.0 ± 13.27<br>CG=44.0 ± 9.95	IG=11(27)<br>CG=11(45)	Metabolic syndrome (MetS)	IG=38.0 ± 6.63<br>CG=34.0 ± 6.63	SphygmoCor	IG=7.93 ± 1.92<br>CG=7.45 ± 1.46	Aerobic training	IG = Treadmill/Elliptical/Cycle<br>• 8 weeks<br>• 3 times/week<br>• 60 min/session<br>• Vigorous (60-85%HRR)
		IG=41.0 ± 13.27<br>CG=40.0 ± 12.65	IG=11(36)<br>CG=10(20)	Healthy	IG=24.0 ± 3.32<br>CG=25.0 ± 3.16	SphygmoCor	IG=6.34 ± 0.83<br>CG=6.06 ± 0.90	Aerobic training	IG = Treadmill/Elliptical/Cycle<br>• 8 weeks<br>• 3 times/week<br>• 60 min/session<br>• Vigorous (60-85%HRR)
Fernandez-del-Valle 2018(Fernandez-del-Valle et al. 2018)	America	IG=22.69 ± 3.35<br>CG=25.00 ± 4.47	IG=6(0)<br>CG=5(0)	Women with obesity	IG=34.19 ± 3.03<br>CG=34.05 ± 3.68	SphygmoCor	IG=6.64 ± 0.52<br>CG=6.66 ± 0.76	Resistance training	IG = Resistance training<br>• 3 weeks<br>• 3 times/week<br>• 50 min/session<br>• Vigorous (70-75%1RM)
Jamka 2021(Jamka et al. 2021)	Poland	IG1=55 ± 7<br>IG2=55 ± 7	IG1=44(0)<br>IG2=41(0)	Abdominally obese women	IG1=35.45 ± 4.51<br>IG2=35.00 ± 4.32	SphygmoCor	IG1=7.1 ± 1.3<br>IG2=6.8 ± 1.2	Aerobic training	IG1 = Cycling<br>• 12 weeks<br>• 3 times/week<br>• 60 min/session<br>• Moderate-Vigorous (50-70%HRmax.)
								Combined training	IG2 = Aerobic + resistance training<br>• 12 weeks<br>• 3 times/week<br>• 60 min/session<br>• Moderate-Vigorous (50-70%HRmax.)<br>• Moderate(50-60% 1RM)
Reed 2023(Reed et al. 2024)	America	IG=53 ± 7<br>CG=51 ± 7	IG=18(32)<br>CG=8(33)	Middle-aged adults with elevated BP/stage-1 hypertension	IG=30.2 ± 4.5<br>CG=30.2 ± 4.6	NIHem	IG=8.27 ± 1.67<br>CG=7.29 ± 0.67	Aerobic training	IG = cycle/treadmills<br>• 12 weeks<br>• 3 times/week<br>• 30-40 min/session<br>• Vigorous (70-85%HRR)
Scott 2019(Scott et al. 2019)	United Kingdom	IG1=29 ± 7.9<br>IG2=29 ± 13.2	IG1=5(71.4)<br>IG2=6(71.4)	Type 1 Diabetes	IG1=29.2 ± 3.2<br>IG2=25.3 ± 3.2	SphygmoCor	IG1=6.1 ± 1.12<br>IG2=6.1 ± 0.98	High-Intensity Interval Training(HIIT)	IG1 = Cycling<br>• 6 weeks<br>• 3 times/week<br>• 15-23 min/session<br>• Vigorous (100% VO2peak)
								Aerobic training	IG2 = Cycling<br>• 6 weeks<br>• 3 times/week<br>• 30-50 min/session<br>• Moderate (65% VO2peak)
O'Gorman 2023(O'Gorman et al. 2023)	Australia	IG=57 ±2<br>CG=51 ± 4	IG=14(100)<br>CG=7(100)	Adults with type 2 diabetes	IG=36.0±1.5<br>CG=36.6±1.9	SphygmoCor		Aerobic training	IG = Aerobic training<br>• 12 weeks<br>• 3 times/week<br>• 50 min/session<br>• Moderate-Vigorous (60-90% VO_2peak)
		IG=56 ± 2<br>CG=53 ± 2	IG=10(0)<br>CG=4(0)	Adults with type 2 diabetes	IG=35.8±1.3<br>CG=34.4±3.8	SphygmoCor		Aerobic training	IG = Aerobic training<br>• 12 weeks<br>• 3 times/week<br>• 50 min/session<br>• Moderate-Vigorous (60-90% VO_2peak)

Madden 2013(Madden et al. 2013)	Canada	IG=68.5 ± 4.5<br>CG=70.0 ± 4.2	IG=25(52)<br>CG=27(63)	Older adults with T2DM, HTN, and Hyperlipidemia	IG=30.9 ± 5.0<br>CG=28.6 ± 4.2	Complior	IG=13.40 ± 3.45<br>CG=12.03 ± 3.27	Aerobic training	IG =treadmill /cycle<br>• 24 weeks<br>• 3 times/week<br>• 60 min/session<br>• Moderate-Vigorous (60-75%HRR)
Lee 2022(E. Lee et al. 2022)	Finland	IG=51 ± 9<br>CG=49 ± 8	IG=16(12.5)<br>CG=16(12.5)	Adults with low physical activity and CVD risk factors	IG=31.3 ± 4.2<br>CG=31. 1 ± 4.7	Pulsepen	IG=9.2 ± 1.7<br>CG=8.5 ± 1.5	Combined training	IG = Aerobic + resistance training<br>• 8 weeks<br>• 3 times/week<br>• 60 min/session<br>• Moderate-Vigorous (50-70%HRmax.)<br>• progressive (65-80% HRmax)
Moncion 2024(Moncion et al. 2024)	Canada	IG1=65.4±8.9<br>IG2=64.4±9.7	IG1=37(64)<br>IG2=30(57)	Individuals post-stroke	IG1=28.0±5.2<br>IG2=29.2±8.2	SPT-301	IG1=9.77±2.51<br>IG2= 10.04±2.49	High-Intensity Interval Training(HIIT)	IG1 = treadmill<br>• 12 weeks<br>• 3 times/week<br>• 24 min/session<br>• Vigorous (80–100%HRR alternating with 30% HRR)
								Aerobic training	IG2 = treadmill<br>• 12 weeks<br>• 3 times/week<br>• 20-30 min/session<br>• Moderate (40%-60% HRR)
Stewart 2005(Stewart et al. 2005)	America	IG=63.0 ± 5.46<br>CG=64. 1 ± 6.31	IG=40(49)<br>CG=42(49)	Untreated mildly hypertensive older adults	IG=29.4 ± 3.83<br>CG=29.7 ± 5.0	Doppler probes	IG=9.01 ± 2.81<br>CG=9.40 ± 3.21	Combined training	IG = Aerobic + resistance training<br>• 26 weeks<br>• 3 times/week<br>• NA<br>• Moderate (50%1RM)<br>• Moderate-vigorous (60-90% HRmax)
Morgan 2023(Morgan et al. 2023)	America	IG1=20.8±1.2<br>IG2= 19.8±1.7<br>CG=20.9±1.8	IG1=8(0)<br>IG2=8(0)<br>CG=8(0)	Healthy, untrained young women	IG1=25.8 ± 3.4<br>IG2=22.4 ± 3.4<br>CG=24.0 ± 3.9	Complior	IG1=6.05 ± 0.80<br>IG2=6.81 ± 1.43<br>CG=6.39 ± 0.73	Resistance training	IG1 = high-intensity (HI) resistance training<br>• 11 weeks<br>• 3-5 times/week<br>• NA<br>• Vigorous (80–90% 1RM)
								Resistance training	IG2= Periodic resistance training<br>• 11 weeks<br>• 3-5 times/week<br>• NA<br>• Moderate (50–70% 1RM)
Ghardashi-Afousi 2025(Ghardashi-A fousi et al. 2025)	Iran	IG=60.15 ± 5.95<br>CG=59.47 ± 6.38	IG=40(55)<br>CG=40(60)	Older adults with mild to moderate hypertension	IG=27.28 ± 1.36<br>CG=27.04 ± 1.36	AtCor Medical	IG=16.29 ± 1.31<br>CG=15.98 ± 1.16	High-Intensity Interval Training(HIIT)	IG = bike<br>• 12 weeks<br>• 3 times/week<br>• 50-60 min/session<br>• Vigorous (85%-90% HRR)
Okamoto 2019(Okamoto et al. 2018)	Japan	IG1=72±4.7<br>IG2=73±4.9	IG1=22(50)<br>IG2=24(41.6)	Healthy	IG1=22.2±2.3<br>IG2=22.2±2.0	PWV/ABI	IG1=12.6±1.97<br>IG2=12.76±2.55	High-Intensity Interval Training(HIIT)	IG1 = Walk<br>• 20 weeks<br>• 3 times/week<br>• 30 min/session<br>• Light/vigorous (40%VO_2max alternate >70%VO_2max.)
								Aerobic training	IG2 = Walk<br>• 20 weeks<br>• 3 times/week<br>• NA<br>• Moderate (50%VO_2max.)

Teixeira do Amaral 2022(Teixeira Do Amaral et al. 2022)	Brazil	IG=51.9 ± 10.2<br>CG=53.3 ± 11.6	IG=12(58)<br>CG=20(40)	Recovering COVID-19 patients	IG=32.6 ± 7.4<br>CG=32.4 ± 7.8	Complior	IG=9.03 ± 2.04<br>CG=8.81 ± 2.66	Combined training	IG = Aerobic + resistance training<br>• 12 weeks<br>• 7 times/week<br>• NA<br>• Moderate-vigorous(RPE 11-17)
Baynard 2024(Baynard et al. 2023)	America	IG1=49 ± 11<br>IG2=44 ± 12	IG1=26(27)<br>IG2=22(27)	multiple sclerosis (MS)	IG1=28.8 ± 5.6<br>IG2=29.2 ± 7.7	SphygmoCor	IG1=6.85 ± 1.88<br>IG2=7.26 ± 1.05	Aerobic training	IG1 =cycle<br>• 12 weeks<br>• 3 times/week<br>• 10-30 min/session<br>• progressive (40-65%VO_2max)
								Stretching training	IG2 =stretching<br>• 12 weeks<br>• 3 times/week<br>• 10-30 min/session<br>• NA
Fujie 2022(Fujie et al. 2022)	Japan	IG=65.5 ± 8.2<br>CG=67.4 ± 6.0	IG=17(41.2)<br>CG=16(37.5)	Healthy	IG=24.41 ± 5.36<br>CG=21.19 ± 4.36	PWV/ABI	IG=8.96 ± 1.32<br>CG=8.27 ± 0.96	Aerobic training	IG =cycle<br>• 8 weeks<br>• 3 times/week<br>• 55 min/session<br>• Moderate-vigorous (60-70%VO_2max)
Tomoto 2023(Tomoto et al. 2023)	America	IG1=68.2 ± 5.3<br>IG2=67.8 ± 4.9	IG1=28(25)<br>IG2=27(30)	Healthy	IG1=25.8 ± 3.9<br>IG2=26.8 ± 3.6	SphygmoCor	IG1=8.99 ± 1.95<br>IG2=8.99 ± 1.80	Aerobic training	IG1 =walk<br>• 52 weeks<br>• 3-5 times/week<br>• progressive 25-50 min/session<br>• Moderate-vigorous (75-90% HRmax)
								Stretching training	IG2 =stretching<br>• 52 weeks<br>• 3-5 times/week<br>• progressive 25-50 min/session<br>• light (<50% HRmax)
Magalhães 2019(Magalhães et al. 2019)	Portugal	IG1=59.7±6.5<br>IG2=56.7±8.3<br>CG=59.0±8.1	IG1=16(44)<br>IG2=13(70)<br>CG=22(50)	Type 2 diabetes	IG1=31.1±5.0<br>IG2=30.1±5.7<br>CG=30.7±5.0	Complior	IG1=13.3±3.8<br>IG2=12.8±4.0<br>CG=13.1±4.8	Combined training	IG1 = MCT + resistance training<br>• 52 weeks<br>• 3 times/week<br>• 42 min/session<br>• Moderate(40-60% HRR)
								Combined training	IG2 = HIIT+ resistance training<br>• 52 weeks<br>• 3 times/week<br>• 34 min/session<br>• 40–60% HRR alternating with 90% HRR
Oliveira 2020(G. H. de Oliveira et al. 2020)	Brazil	IG1=29.4 ± 4.6<br>IG2=27.0 ± 5.0	IG1=11(0)<br>IG2=14(0)	Young obese women	IG1=34.1 ± 3.6<br>IG2=35.7 ± 2.4	SphygmoCor	IG1=6.6 ± 0.8<br>IG2=6.5 ± 0.8	High-Intensity Interval Training(HIIT)	IG1 = running<br>• 8 weeks<br>• NA<br>• NA<br>• 85–95% HRmax alternating with 65-75% HRmax
								Aerobic training	IG2 = running<br>• 8 weeks<br>• 3 times/week<br>• 41 min/session<br>• Moderate (65-75% HRmax)
Shiotsu 2018(Shiotsu et al. 2018)	Japan	IG=70.0 ± 4.3<br>CG=71.0 ± 4.4	IG=30(100)<br>CG=10(100)	Healthy	IG=23.85 ± 2.61<br>CG=23.8 ± 2.5	VaSera	IG=8.55 ± 0.47<br>CG=8.30 ± 0.14	Combined training	IG = Aerobic + Resistance<br>• 10 weeks<br>• 2 times/week<br>• 40 min/session<br>• 60% HRR alternating with 70-80% 1RM

Tomoto 2021(Tomoto et al. 2021)	America	IG1=64.8 ± 6.4<br>IG2=66.1 ± 6.8	IG1=18(45)<br>IG2=28(47)	Amnestic mild cognitive impairment (MCI) patients	IG1=27.3 ± 4.8<br>IG2=28.2 ± 4.2	SphygmoCor	IG1=8.73 ± 1.97<br>IG2=9.95 ± 1.97	Aerobic training	IG =walk<br>• 52 weeks<br>• 3-5 times/week<br>• progressive 25-40 min/session<br>• Moderate-vigorous (75-90% HRmax)
								Stretching training	IG2 =stretching<br>• 52 weeks<br>• 3-5 times/week<br>• progressive 25-40 min/session<br>• light (<50% HRmax)
Way 2020(Way et al. 2020)	Australia	IG1=54.8 ± 2.4<br>IG2=56.9 ± 2.1<br>CG=51.9 ± 1.4	IG1=12(50)<br>IG2=12(58)<br>CG=11(64)	Obese, inactive adults with type 2 diabetes	IG1=34.3 ± 3.7<br>IG2=37.5 ± 5.4<br>CG=35.8 ± 5.7	SphygmoCor	IG1=8.5 ± 1.5<br>IG2=8.8 ± 1.6<br>CG=8.4 ± 2.6	Aerobic training	IG1 =cycling<br>• 12 weeks<br>• 3 times/week<br>• 55 min/session<br>• Moderate (60% VO_{2peak})
								High-Intensity Interval Training(HIIT)	IG2 =cycling<br>• 12 weeks<br>• 3 times/week<br>• 19 min/session<br>• Vigorous (90% VO_{2peak} alternate 50% VO2peak)
Zhang 2025(Zhang et al. 2025)	China	IG=19.13 ± 1.45<br>CG=19.25 ± 1.81	IG=16(100)<br>CG=16(100)	Healthy	IG=21.06 ± 2.14<br>CG=21.92 ± 2.38	PowerLab	IG=6.14 ± 0.86<br>CG=6.03 ± 1.13	Resistance training	IG = resistance training<br>• 8 weeks<br>• 3 times/week<br>• 60 min/session<br>• Moderate (65% 1RM)
Alvarez-Alvarado 2017(Alvarez-Alvarado et al. 2017)	America	IG=20±5.0<br>CG=21±3.6	IG=25(0)<br>CG=13(0)	young overweight/obese women	IG=30.7±3.5<br>CG=31.3±4.0	VP-2000	IG=7.8±1.0<br>CG=7.7±0.36	Whole-body vibration training (WBVT)	IG =leg exercises<br>• 6 weeks<br>• 3 times/week<br>• 11-30 min/session<br>• Moderate (30-35 Hz)
Petrick 2021(Petrick et al. 2021)	Canada	IG1=35.3 ± 15.1<br>IG2=39.4 ± 14.9	IG1=11(100)<br>IG2=12(100)	Overweight/obese males	IG1=33.9 ± 2.4<br>IG2=34.1 ± 4.3	Sphygmocor	IG1=6.77 ± 1.41<br>IG2=6.72 ± 1.0	Aerobic training	IG1 =cycling<br>• 6 weeks<br>• 5 times/week<br>• 30-40 min/session<br>• Moderate (60% Wpeak)
								High-Intensity Interval Training(HIIT)	IG2 =cycling<br>• 6 weeks<br>• 3 times/week<br>• 13-18 min/session<br>• Vigorous (170% Wpeak)
Ciolac 2010(Ciolac et al. 2010)	Brazil	IG1=26.6 ± 4.9<br>CG1=25.3 ± 3.7	IG1= 16 (0)<br>CG1=12 (0)	Healthy	IG1=23.5 ± 4.8 CG1=23.8 ± 3.9	Complior	IG1= 7.47±0.48<br>CG1=7.38±0.67	High-Intensity Interval Training(HIIT)	IG1 = Walk/run<br>• 16 weeks<br>• 3 times/week<br>• 60 min/session<br>•Moderate/vigorous(50–60% VO_{2max} alternate 80-90% VO_{2max})
		IG2=24.4 ± 3.8<br>CG2=25.3 ± 3.1	IG2= 16 (0)<br>CG2=15 (0)		IG2=24.3 ± 4.6<br>CG2=23.5 ± 2.4		IG2=7.55±0.73<br>CG2=6.93±0.18	Aerobic training	IG2 = Walk/run<br>• 16 weeks<br>• 3 times/week<br>• 60 min/session<br>• Moderate-Vigorous (60-70% VO_{2max})
Dobrosielski 2012(Dobrosielski et al. 2012)	America	IG=57.0 ± 6.0<br>CG=56.0 ± 6.0	IG=70 (59)<br>CG=70 (57)	type 2 diabetes (T2DM)	IG=33.0 ± 0.6<br>CG=33.6 ± 0.5	Doppler	IG=9.22±3.36<br>CG=9.09±3.58	Combined training	IG = Aerobic exercise (walk/run) + Resistance training<br>• 24 weeks<br>• 3 times/week<br>• 60 minutes/ session<br>• Moderate- Vigorous (65–90% HR_{max})

Goldberg 2012(Goldberg et al. 2012)	Australia	IG=20.5 ± 1.94<br>CG=20.7 ± 1.94	IG= 15 (0)<br>CG= 15 (0)	Healthy	IG=23.3 ± 1.94<br>CG=23.4 ± 2.32	Sphygmocor	IG=5.10 ± 1.16<br>CG=5.00 ±0.77	Aerobic training	IG= Bike<br>• 4 weeks<br>• 3 times/week<br>• 30 min/session<br>• Vigorous (65%VO_{2max})
Hasegawa 2018(Hasegawa et al. 2018)	Japan	IG1=23.10 ± 7.14<br>IG2=23.70 ± 2.91<br>CG=20.70 ± 0.53	IG1=7(100)<br>IG2=7(100)<br>CG=7(100)	Healthy	IG1=21.60±1.59<br>IG2=22.10 ± 1.59<br>CG=24.80 ± 3.44	PWV/ABI	IG1=9.14±0.96<br>IG2=9.00 ±0.94<br>CG=7.51± 1.02	Aerobic training	IG1 = Bike<br>• 8 weeks<br>• 3 times/week<br>• 45 min/session<br>• Moderate-vigorous (60-70%VO_{2max})
								Sprint Interval Training(SIT)	IG2 = Bike<br>• 6 weeks<br>• 4 times/week<br>• NA<br>• Vigorous (170%VO_{2max} alternate repose)
Deiseroth 2019(Deiseroth et al. 2019)	Switzerland	IG=58.0 ± 5.0<br>CG=57.0 ± 6.0	IG=42 (47.4)<br>CG=37 (63.3)	Sedentary	IG=33.3 ± 3.0<br>CG=33.1 ± 5.1	Sphygmocor	IG=8.2 ± 1.2<br>CG=8.2 ± 1.6	High-Intensity Interval Training(HIIT)	IG = Walk<br>• 12 weeks<br>• 3 times/week<br>• 60 min/session<br>• Moderate/Vigorous<br>(60-70%HRmax alternate 80-90%HRmax.)

Abbreviations: IG, intervention group; CG, control group; PE, physical exercise; BMI, body mass index; cfPWV, carotid–femoral pulse wave velocity; HRmax, maximum heart rate; HRR, heart -rate reserve; VO_2max, maximal oxygen uptake; RPE, rating of perceived exertion; 1RM, one-repetition maximum.

Appendix 3. Dataset of the dose-response meta-analysis

StudyID	Frequency times/ week	Intensity METs	Time min/times	Agent	Exact_dose	Dose	Residual dose	y	se	N
Rugolo 2019	3	4.5	30	AT	405	300	105	-0.4	0.775	15
Rugolo 2019	0	0	0	CON	0	0	0	-0.2	0.942	15
Greenwood 2015(b)	3	7	60	AT	1260	1200	60	-0.6	0.419	13
Greenwood 2015(b)	3	3.5	60	RT	630	600	30	-1.4	0.455	13
Greenwood 2015(b)	0	0	0	CON	0	0	0	0.5	0.514	20
Chrysohoou 2015	3	7	45	CT	945	900	45	-0.8	0.454	33
Chrysohoou 2015	0	0	0	CON	0	0	0	0.3	0.269	39
Taylor 2020	3	4.25	40	IT	510	600	90	-0.5	0.204	28
Taylor 2020	3	4.5	40	AT	540	600	60	-0.4	0.178	26
Greenwood 2015(a)	3	10.5	40	CT	1260	1200	60	-1.6	0.746	8
Greenwood 2015(a)	0	0	0	CON	0	0	0	1	0.705	10
Kim 2021	2	3.82	55	RT	420	300	120	0.03	0.08	16
Kim 2021	0	0	0	CON	0	0	0	-0.02	0.141	8
Lee 2015	3	3.33	60	CT	600	600	0	-0.78	0.465	14
Lee 2015	0	0	0	CON	0	0	0	0.97	0.704	12
Holloway 2016	3	9	15	IT	405	300	105	-1	0.289	12
Holloway 2016	0	0	0	CON	0	0	0	0	0.333	9
Jeong 2023	3	4.15	32.5	AT	405	300	105	0.2	0.43	32
Jeong 2023	3	2.3	32.5	ST	224	300	76	-0.1	0.277	26
Koh 2010	3	4.5	30	AT	405	300	105	-0.3	0.736	15
Koh 2010	3	3.5	30	AT	315	300	15	-0.2	0.852	15
Koh 2010	0	0	0	CON	0	0	0	0.5	0.78	16
Heydari 2013	3	8	20	IT	480	600	120	-0.4	0.215	20
Heydari 2013	0	0	0	CON	0	0	0	0.31	0.184	18
Thompson 2022	3	3.14	55	CT	517.5	600	82.5	0.11	0.803	21
Thompson 2022	0	0	0	CON	0	0	0	0	0.767	23
Kobayashi 2022	2	4.5	30	AT	270	300	30	-1.53	0.106	15
Kobayashi 2022	4	4.5	30	AT	540	600	60	-1.58	0.075	15
Kobayashi 2022	0	0	0	CON	0	0	0	-0.06	0.08	15
Cortez-Cooper 2008	3	3.27	37.5	RT	367.5	300	67.5	-0.61	0.344	13
Cortez-Cooper 2008	4	6	37.5	CT	900	900	0	-0.55	0.294	12

Cortez-Cooper 2008	4	1.61	37.5	ST	241.5	300	58.5	-0.01	0.546	12
Slivovskaja 2017	5	5	35	AT	875	900	25	-0.46	0.141	84
Slivovskaja 2017	0	0	0	CON	0	0	0	-0.12	0.16	42
Figueroa 2015	3	3.75	20	WBVT	225	300	75	-0.3	0.337	24
Figueroa 2015	0	0	0	CON	0	0	0	0.4	0.557	12
Lin 2021	3	4.5	30	AT	405	300	105	-0.57	0.668	13
Lin 2021	3	3	30	CT	270	300	30	-0.7	0.95	12
Lin 2021	0	0	0	CON	0	0	0	0.51	1.393	9
Pascoalino 2014	3	6	40	AT	720	600	120	-0.34	0.345	31
Pascoalino 2014	0	0	0	CON	0	0	0	0.01	0.87	9
Farah 2018	3	3.44	12	IHT	124	300	176	-0.87	0.308	32
Farah 2018	0	0	0	CON	0	0	0	0.1	0.5	16
Guimarães 2010	3	5.67	60	CT	1020	900	120	-0.36	0.348	16
Guimarães 2010	0	0	0	CON	0	0	0	0.3	0.573	11
Lai 2025	3	4.5	20	AT	270	300	30	-1.84	0.276	75
Lai 2025	0	0	0	CON	0	0	0	1.55	0.493	39
Croymans 2014	3	4	60	RT	720	600	120	-0.2	0.268	28
Croymans 2014	0	0	0	CON	0	0	0	0.45	0.301	8
Cox 2024	3	4.77	26	CT	372	300	72	0.4	0.25	23
Cox 2024	4	5.21	52.5	CT	1095	1200	105	0	0.125	23
Cox 2024	0	0	0	CON	0	0	0	0	0.25	23
Cooke 2018	3	4.5	42.6	AT	575	600	25	-0.83	0.585	10
Cooke 2018	0	0	0	CON	0	0	0	0.33	0.234	10
Van Craenenbroeck 2015	7	6	40	AT	1680	1800	120	-0.13	0.236	19
Van Craenenbroeck 2015	0	0	0	CON	0	0	0	-0.87	0.362	21
Yoshizawa 2008	2	4.5	30	AT	270	300	30	−0.32	0.294	12
Yoshizawa 2008	0	0	0	CON	0	0	0	0.61	0.286	12
Chang 2025	7	4	22	AT	616	600	16	0.08	0.284	21
Chang 2025	0	0	0	CON	0	0	0	1.8	0.788	14
Correia 2020	3	2.5	20	IHT	150	300	150	-0.2	0.457	43
Correia 2020	0	0	0	CON	0	0	0	-0.5	0.458	41
Banks 2023	3	3.5	40	RT	420	300	120	-0.2	0.283	13
Banks 2023	0	0	0	CON	0	0	0	-0.1	0.283	13
Blumenthal 2021	3	5.33	45	AT	720	600	120	-0.1	0.385	90
Blumenthal 2021	0	0	0	CON	0	0	0	0	0.373	50
Brubaker 2022	3	4.5	40	AT	540	600	60	-0.72	0.367	38
Brubaker 2022	3	4.67	60	CT	840	900	60	-0.84	0.347	39
Choo 2014	3	4.5	60	AT	810	900	90	0.11	0.209	50
Choo 2014	3	3.5	60	RT	630	600	30	-0.15	0.217	30
Choo 2014	3	5.25	60	CT	945	900	45	-0.03	0.175	30
Donley 2014(a)	3	6	60	AT	1080	1200	120	-0.69	0.525	11
Donley 2014(a)	0	0	0	CON	0	0	0	-0.01	0.407	11
Donley 2014(b)	3	6	60	AT	1080	1200	120	0.25	0.302	11
Donley 2014(b)	0	0	0	CON	0	0	0	0.8	0.392	10
Fernandez-del-Valle 2018	3	4	50	RT	600	600	0	0.09	0.335	6
Fernandez-del-Valle 2018	0	0	0	CON	0	0	0	0.04	0.353	5
Jamka 2021	3	4.5	60	AT	810	900	90	-0.6	0.181	44
Jamka 2021	3	3.42	60	CT	615	600	15	-0.2	0.25	41
Reed 2023	3	6	35	AT	630	600	30	-0.09	0.361	18
Reed 2023	0	0	0	CON	0	0	0	0.82	0.53	8
Scott 2019	3	7.16	19	IT	408	300	108	-0.7	0.626	5
Scott 2019	3	4.5	40	AT	540	600	60	-0.7	0.4	6
O'Gorman 2023(a)	3	2.01	50	AT	302.1	300	2.1	-0.12	0.289	14
O'Gorman 2023(a)	0	0	0	CON	0	0	0	0.03	0.329	7
O'Gorman 2023(b)	3	2.01	50	AT	302.1	300	2.1	-1.25	0.658	10
O'Gorman 2023(b)	0	0	0	CON	0	0	0	2.95	1.355	4
Madden 2013	3	6	60	AT	1080	1200	120	1.21	0.7	25
Madden 2013	0	0	0	CON	0	0	0	-0.27	0.699	27
Dobrosielski 2012	3	6.35	60	CT	1143	1200	57	0.08	0.399	70

Dobrosielski 2012	0	0	0	CON	0	0	0	0.01	0.429	70
Lee 2022	3	5	60	CT	900	900	0	0	0.393	16
Lee 2022	0	0	0	CON	0	0	0	0.2	0.525	16
Moncion 2024	3	6.73	24	IT	484.5	600	115.5	0.54	0.464	37
Moncion 2024	3	4.5	25	AT	337.5	300	37.5	-0.51	0.561	30
Ghardashi-Afousi 2025	3	5.67	55	IT	936	900	36	-3.06	0.079	40
Ghardashi-Afousi 2025	0	0	0	CON	0	0	0	0.31	0.101	40
Baynard 2024	3	4.5	20	AT	270	300	30	1.02	0.339	26
Baynard 2024	3	2.3	20	ST	138	300	162	0	0.424	22
Fujie 2022	3	6	55	AT	990	900	90	-1.61	0.308	17
Fujie 2022	0	0	0	CON	0	0	0	-0.37	0.295	16
Tomoto 2023	4	7.2	37.5	AT	1080	1200	120	-0.36	0.372	28
Tomoto 2023	4	2.11	37.5	ST	316	300	16	-0.1	0.316	27
Magalhães 2019	3	5.91	42	CT	744.3	600	144.3	0.87	0.748	29
Magalhães 2019	0	0	0	CON	0	0	0	0.9	0.974	22
Shiotsu 2018	2	6	40	CT	480	600	120	-0.19	0.15	30
Shiotsu 2018	0	0	0	CON	0	0	0	0.4	0.044	10
Tomoto 2021	4	8.31	32.5	AT	1080	1200	120	-0.61	0.441	18
Tomoto 2021	4	2.38	32.5	ST	310	300	10	-0.16	0.355	28
Way 2020	3	4.5	55	AT	742.5	600	142.5	-0.12	0.323	12
Way 2020	3	8.5	19	IT	484.5	600	115.5	-0.33	0.245	12
Way 2020	0	0	0	CON	0	0	0	0.8	0.482	11
Zhang 2025	3	3.5	60	RT	630	600	30	-0.77	0.195	16
Zhang 2025	0	0	0	CON	0	0	0	0.05	0.303	16
Alvarez-Alvarado 2017	3	2.5	20.5	WBVT	154	300	146	-0.26	0.09	25
Alvarez-Alvarado 2017	0	0	0	CON	0	0	0	0.1	0.089	13
Petrick 2021	5	4.5	35	AT	787.5	900	112.5	-0.25	0.398	11
Petrick 2021	3	8.71	15.5	IT	405	300	105	0.17	0.329	12
Ciolac 2010(a)	3	5.33	60	IT	960	900	60	-0.41	0.113	16
Ciolac 2010(a)	0	0	0	CON	0	0	0	0.15	0.182	12
Ciolac 2010(b)	3	3	60	AT	540	600	60	-0.32	0.173	16
Ciolac 2010(b)	0	0	0	CON	0	0	0	0.04	0.07	15
Goldberg 2012	3	4.5	30	AT	405	300	105	-0.3	0.263	15
Goldberg 2012	0	0	0	CON	0	0	0	0.2	0.199	15
Hasegawa 2018	3	4.5	45	AT	607.5	600	7.5	-1.58	0.457	7
Hasegawa 2018	0	0	0	CON	0	0	0	0.71	0.612	7
Deiseroth 2019	3	8.5	60	IT	1530	1500	30	-0.1	0.177	42
Deiseroth 2019	0	0	0	CON	0	0	0	-0.3	0.263	37

This supplementary file presents the study-arm-level dataset used for the model-based network meta-analysis of the relationship between exercise dose and carotid-femoral pulse wave velocity (cfPWV). StudyID indicates the first author and publication year. Frequency indicates the number of exercise sessions per week. Intensity indicates the average energy expenditure per exercise session used for dose calculation, expressed in metabolic equivalents (METs). For single-component interventions, MET values were matched to the closest applicable activity entry in the 2024 Adult Compendium of Physical Activities; for combined training (CT) and interval training (IT), session-average MET values were calculated as time-weighted averages of component activities or work–recovery periods. Time indicates the duration of each exercise session in minutes. Agent indicates the exercise modality: aerobic training (AT), interval training (IT), combined training (CT), resistance training (RT), stretching training (ST), isometric handgrip training (IHT), whole-body vibration training (WBVT), or non-exercise control (CON). Exact_dose indicates the estimated weekly exercise dose, calculated as METs × minutes per session × sessions per week and expressed as MET·min/week. Dose indicates the approximated dose category used in the model (0, 300, 600, 900, 1200, 1500 MET·min/week). Residual_dose indicates the absolute difference between Exact_dose and the assigned Dose category. The y value represents the observed mean change in cfPWV for each study arm (m/s), and se represents its standard error, calculated as the standard deviation of change divided by the square root of the sample size (N). N indicates the number of participants in each study arm.

Appendix 4. RoB 2 assessment of included randomized controlled trials.

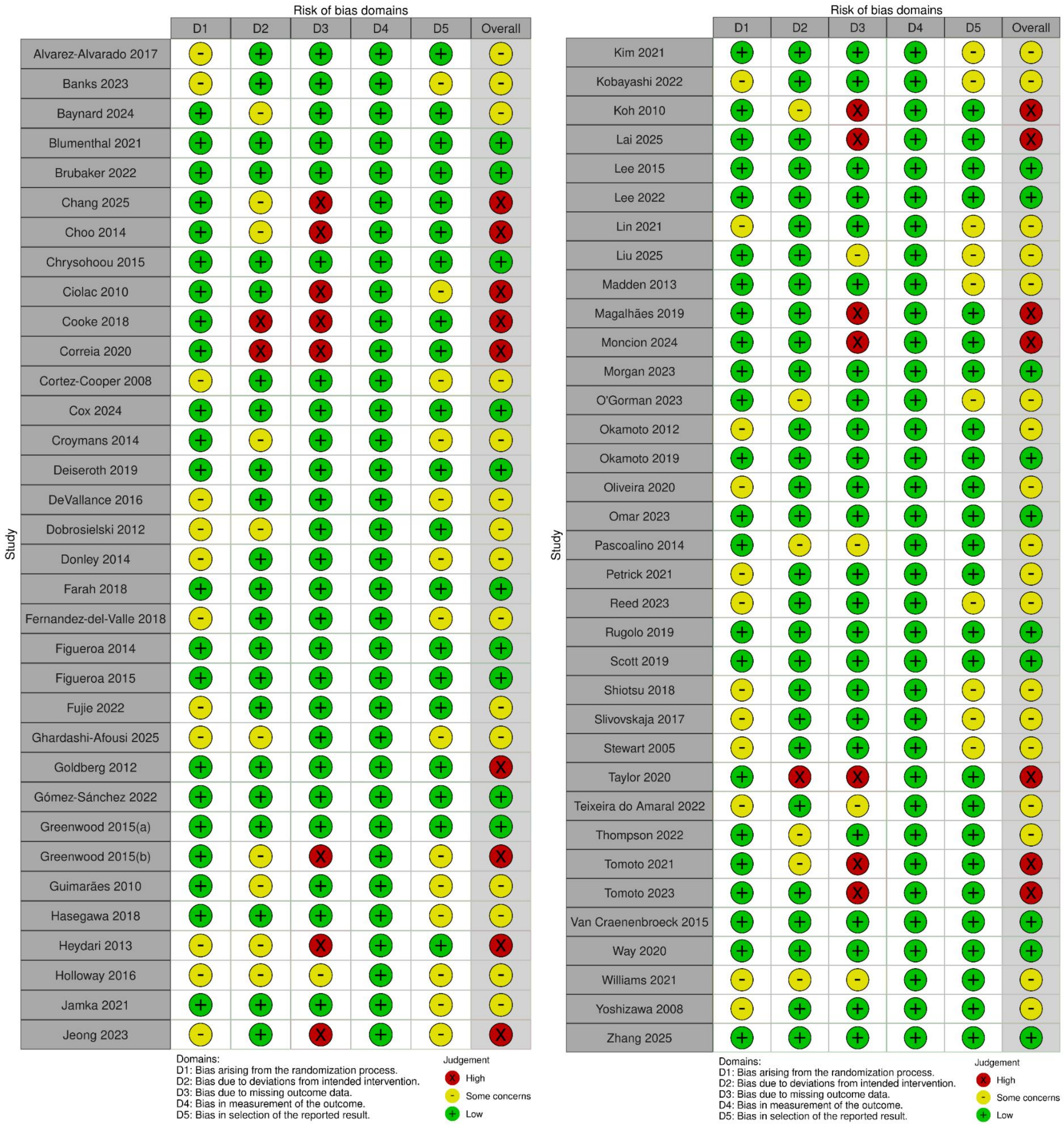

Appendix 5. Summary plot of RoB 2 judgments across the five risk-of-bias domains and overall risk of bias.

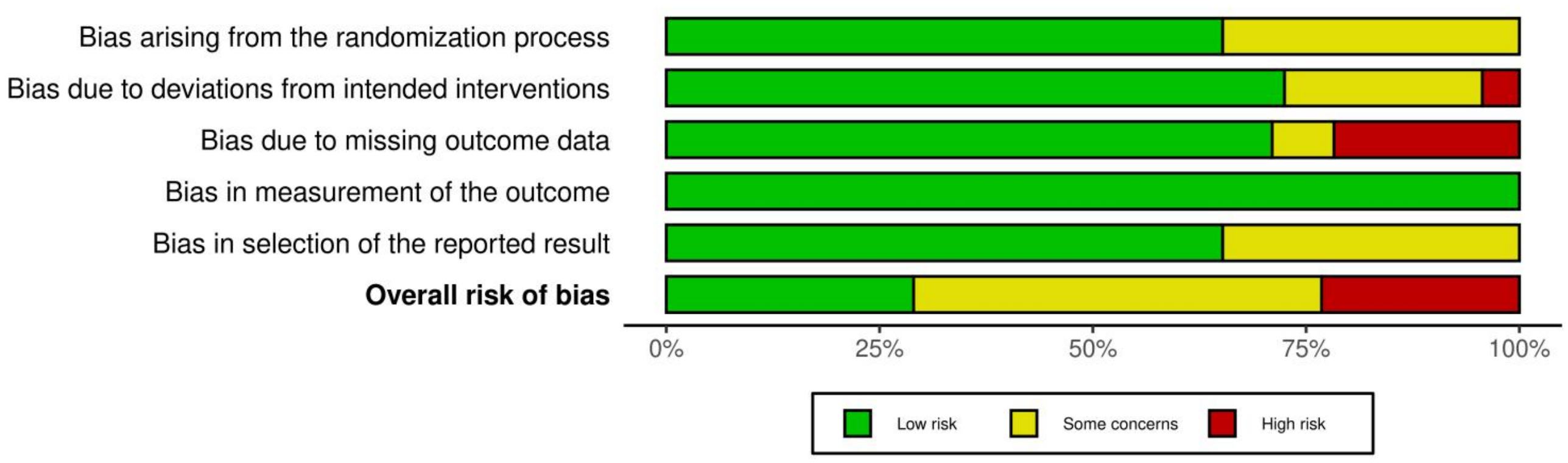

Appendix 6. Funnel plot of carotid–femoral pulse wave velocity.

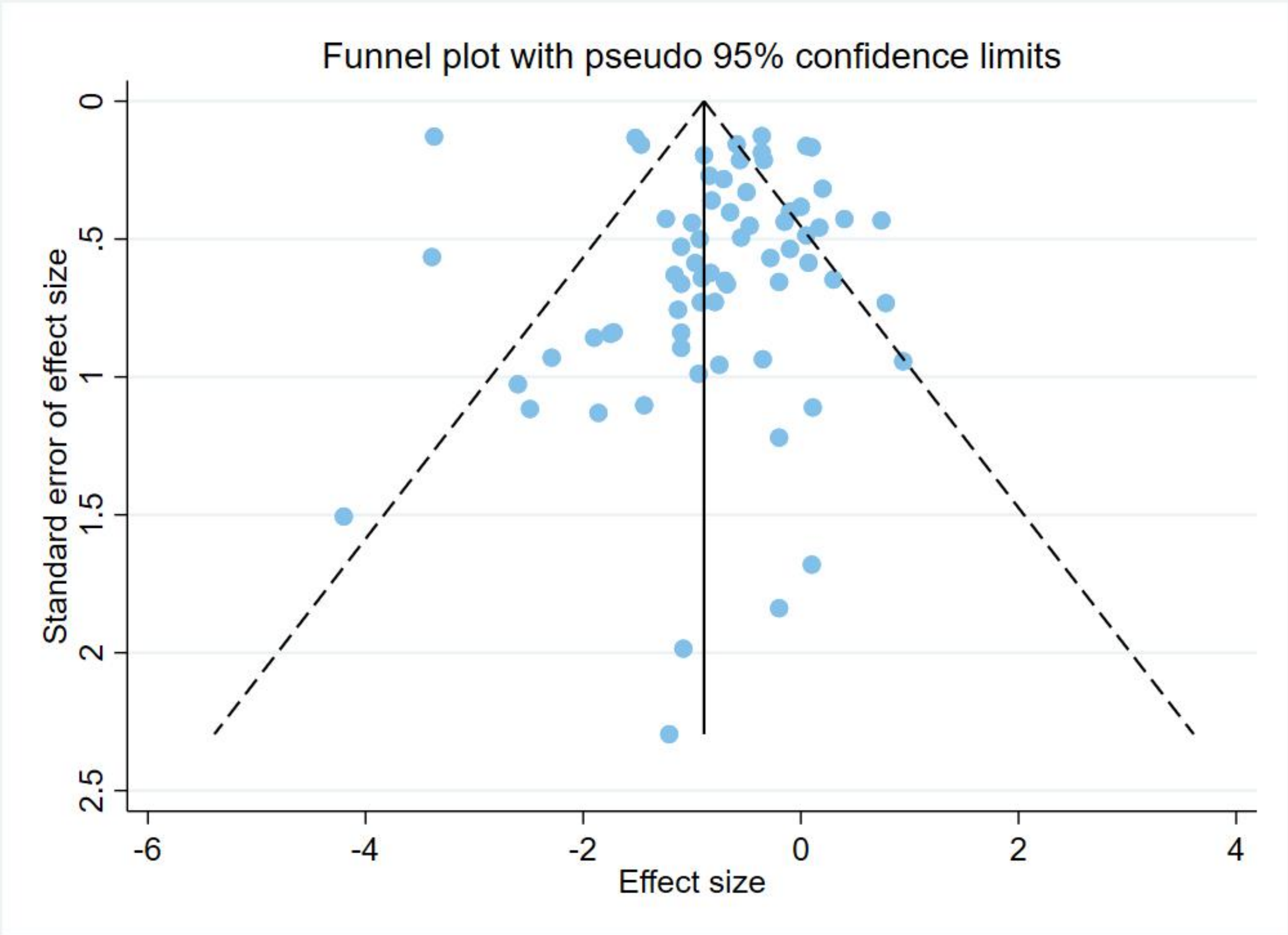

Appendix 7. Forest plot of the effects of exercise versus non-exercise control on carotid-femoral pulse wave velocity (cfPWV) in adults. CON, non-exercise control; AT, aerobic training; RT, resistance training; CT, combined training; IT, interval training; WBVT, whole-body vibration training; IHT, isometric handgrip training; ST, stretching training.

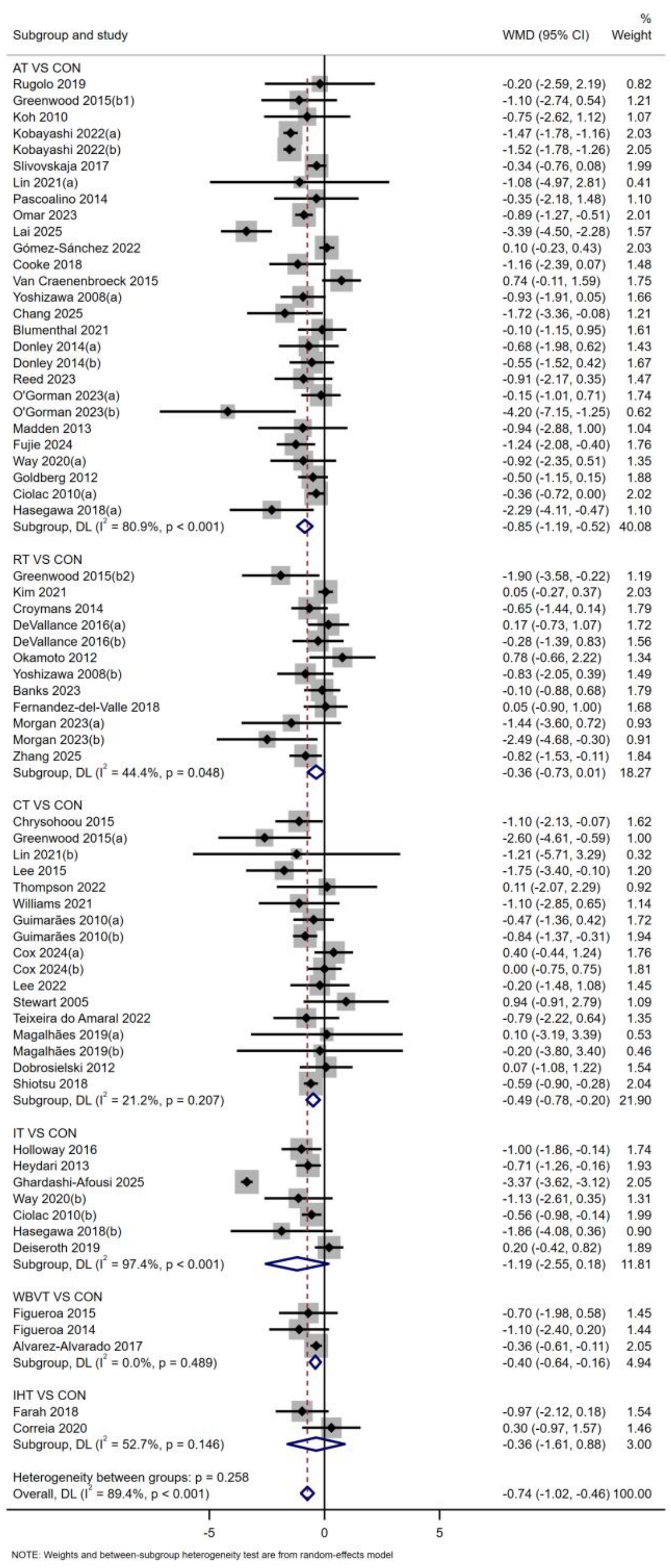

Appendix 8. Sensitivity analysis

(A) Baujat plot of study contributions to overall heterogeneity and pooled effect.

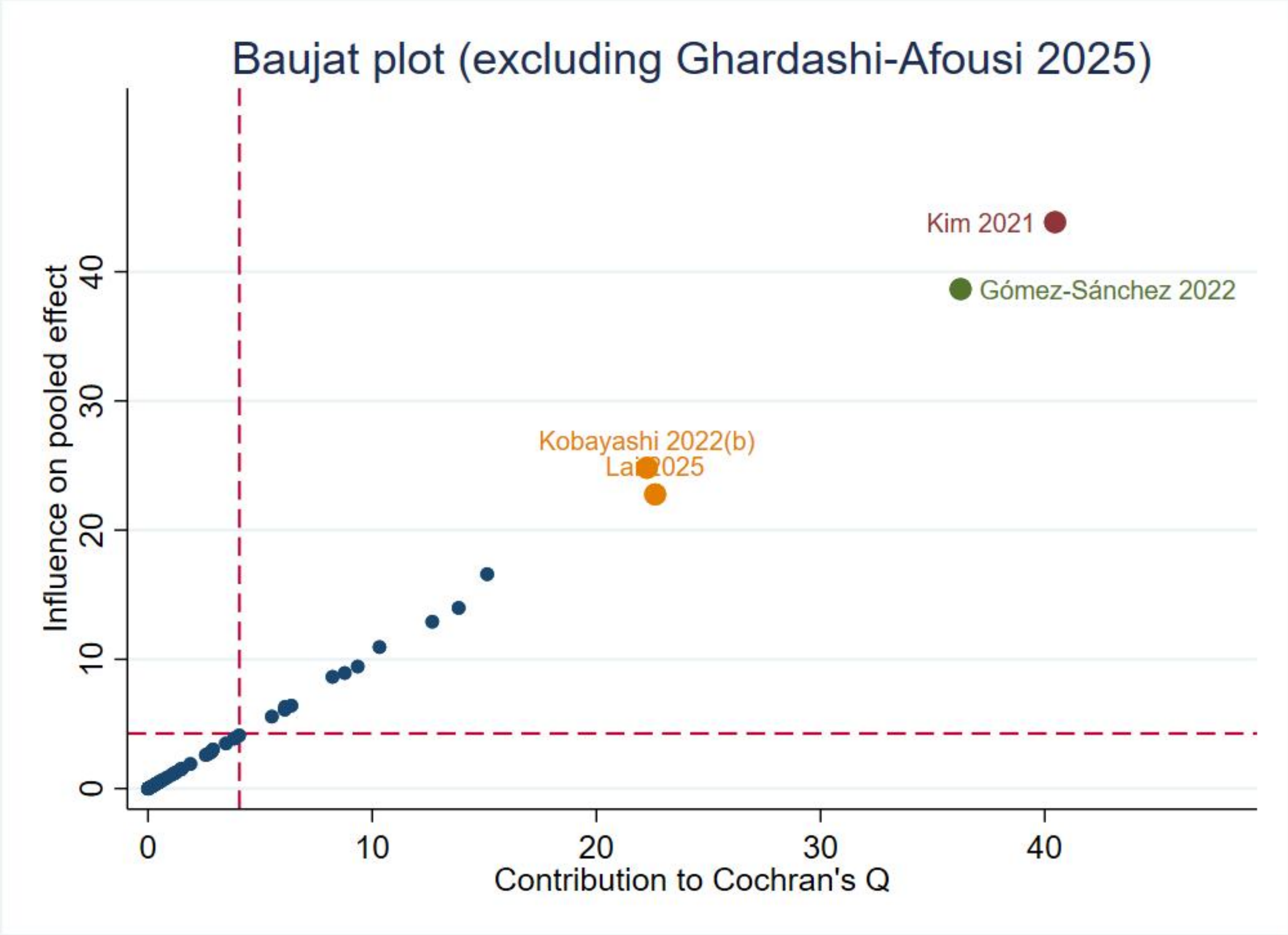

(B) Pooled effect estimates after excluding specified studies.

Excluded literature	WMD (95% CI)	I^2	P
Ghardashi-Afousi 2025	-0.64 (-0.82,-0.45)	70.2	< 0.001
Ghardashi-Afousi 2025 and Kim 2021	-0.65 (-0.84, -0.47)	68	< 0.001
Ghardashi-Afousi 2025 and Gómez-Sánchez 2022	-0.66 (-0.84,-0.47)	67.7	< 0.001

(C) Sensitivity analyses of pooled pairwise effects using alternative correlation coefficients for change-score SDs

Comparison	r = 0.3		r = 0.5		r = 0.7	
	WMD (95% CI)	I^2 (%)	WMD (95% CI)	I^2 (%)	WMD (95% CI)	I^2 (%)
Overall exercise vs CON	-0.73 (-0.95, -0.51)	70.7	-0.74 (-1.02, -0.46)	89.4	-0.74 (-0.95, -0.53)	86.6
AT vs CON	-0.87 (-1.21, -0.53)	72.6	-0.86 (-1.19, -0.52)	80.8	-0.89 (-1.21, -0.57)	87
RT vs CON	-0.35 (-0.71, 0.01)	31.1	-0.36 (-0.73, 0.01)	44.4	-0.40 (-0.75, -0.04)	64.8
CT vs CON	-0.56 (-0.75, -0.37)	0	-0.49 (-0.78, -0.20)	21.2	-0.50 (-0.83, -0.16)	39.5
IT vs CON	-1.18 (-2.27, -0.09)	91.5	-1.19 (-2.55, 0.18)	97.4	-1.19 (-2.26, -0.12)	96.3
WBVT vs CON	-0.44 (-0.85, -0.03)	0	-0.40 (-0.64, -0.16)	0	-0.46 (-0.77, -0.15)	2.1
IHT vs CON	-0.37 (-1.62, 0.87)	34.4	-0.36 (-1.61, 0.88)	52.7	-0.35 (-1.60, 0.89)	70.7

(D) Sensitivity analysis of pooled pairwise effects using alternative random-effects methods

Comparison	DL random-effects WMD (95% CI)	REML random-effects WMD (95% CI)	REML + HKSJ WMD (95% CI)
Overall exercise vs CON	−0.74 (−1.02, −0.46)	−0.72 (−0.94, −0.50)	−0.72 (−0.95, −0.50)
AT vs CON	−0.85 (−1.19, −0.52)	−0.85 (−1.19, −0.52)	−0.85 (−1.21, −0.50)
RT vs CON	−0.36 (−0.73, 0.01)	−0.34 (−0.67, −0.00)	−0.34 (−0.76, 0.08)
CT vs CON	−0.49 (−0.78, −0.20)	−0.49 (−0.78, −0.20)	−0.49 (−0.81, −0.17)
IT vs CON	−1.19 (−2.55, 0.18)	−1.18 (−2.13, −0.23)	−1.18 (−2.36, 0.01)
WBVT vs CON	−0.40 (−0.64, −0.16)	−0.40 (−0.64, −0.16)	−0.40 (−0.92, 0.13)
IHT vs CON	−0.36 (−1.61, 0.88)	−0.36 (−1.61, 0.88)	−0.36 (−8.42, 7.70)

Appendix 9. Inconsistency of the outcome indicator assessed using loop-specific heterogeneity estimates, an inconsistency model, and node-splitting analysis. Node codes: A, CON (non-exercise control); B, AT (aerobic training); C, RT (resistance training); D, CT (combined training); E, IT (interval training); F, WBVT (whole-body vibration training); G, IHT (isometric handgrip training); H, ST (stretching training).

Appendix 9-1

**loop-specific heterogeneity estimates**						
Loop	IF	seIF	z_value	p_value	CI_95	Loop_Heterog_tau2
RT-CT-ST	2.498	0.908	2.75	0.006	(0.72,4.28)	0
AT-RT-CT	1.512	1.89	0.8	0.424	(0.00,5.22)	3.281
CON-RT-CT	0.973	0.884	1.101	0.271	(0.00,2.70)	1.316
AT-CT-ST	0.755	0.759	0.994	0.32	(0.00,2.24)	0.049
CON-AT-RT	0.666	0.55	1.212	0.226	(0.00, 1.74)	0.444
CON-AT-IT	0.503	0.617	0.815	0.415	(0.00, 1.71)	0.987
AT-RT-ST	0.476	0.736	0.647	0.518	(0.00, 1.92)	0
CON-AT-CT	0.117	0.541	0.216	0.829	(0.00, 1. 18)	0.434

loop-specific heterogeneity estimates after exclusion of Liu 2025						
Loop	IF	seIF	z_value	p_value	CI_95	Loop_Heterog_tau2
AT-CT-ST	0.755	0.759	0.994	0.32	(0.00,2.24)	0.049
CON-AT-RT	0.666	0.55	1.212	0.226	(0.00, 1.74)	0.444
CON-AT-IT	0.503	0.617	0.815	0.415	(0.00, 1.71)	0.987
AT-RT-ST	0.476	0.736	0.647	0.518	(0.00, 1.92)	0
CON-RT-CT	0.311	0.448	0.693	0.488	(0.00, 1. 19)	0.12
AT-RT-CT	0.247	0.494	0.5	0.617	(0.00, 1.22)	0
CON-AT-CT	0.117	0.541	0.216	0.829	(0.00, 1. 18)	0.434
RT-CT-ST	0 06	0 937	0 064	0 949	(0 00, 1 90)	0

Appendix 9-2

Inconsistency model		
	Overall network	Network after exclusion of Liu 2025
chi2	27.63	6.99
Prob > chi2	0.0037	0.7266

Appendix 9-3

Node splitting analysis							
Side	Direct Coef.	Std. Err.	Indirect Coef.	Std. Err.	Difference Coef.	Std. Err.	P>|z|
A B	-0.7679939	0.1970572	-0.6276337	0.3675262	-0.1403601	0.4179115	0.737
A C	-0.3642712	0.2959714	0.0665699	0.4783794	-0.4308411	0.5624201	0.444
A D	-0.5999348	0.3124821	-1.30729	0.4094796	0.7073549	0.5151665	0.17
A E	-1.195632	0.3436225	-0.5473935	0.4236514	-0.6482387	0.5468923	0.236
B C	-0.2542601	0.5056512	0.8107039	0.3301327	-1.064964	0.6021248	0.077
B D	0.0579828	0.5196225	-0.1957504	0.3296686	0.2537332	0.6155142	0.68
B E	0.0410641	0.3424652	-0.5686555	0.4208071	0.6097197	0.5420576	0.261
B H	-0.1512728	0.4865316	0.7879356	0.9521132	-0.9392084	1.06922	0.38
C D	-1.274749	0.4954213	-0.2014232	0.3951097	-1.073326	0.6336801	0.09
C H	0.6104124	1.032863	-0.745392	0.5535675	1.355804	1.162258	0.243
D H	0.4119325	1.024695	0.0961008	0.5562623	0.3158317	1.154974	0.785

Node splitting analysis after exclusion of Liu 2025							
Side	Direct Coef.	Std. Err.	Indirect Coef.	Std. Err.	Difference Coef.	Std. Err.	P>|z|
A B	-0.7561116	0.1705583	-0.6206417	0.3139521	-0.1354699	0.358188	0.705
A C	-0.3546317	0.2550919	-0.9062473	0.4630602	0.5516156	0.5281925	0.296
A D	-0.5862656	0.2793207	-0.6236794	0.3869138	0.0374137	0.4769916	0.937
A E	-1.205437	0.2876289	-0.5447442	0.3615403	-0.6606924	0.4635198	0.154
B C	-0.2560354	0.4442851	0.4633669	0.2971097	-0.7194023	0.5324687	0.177
B D	0.0596965	0.4395175	0.1567138	0.2991521	-0.0970173	0.5318282	0.855
B E	0.0320724	0.2927604	-0.5937525	0.3512893	0.6258249	0.4568077	0.171
B H	-0.1507103	0.4230324	0.7937084	0.8479623	-0.9444187	0.9476258	0.319
C D	0.0814746	0.539193	-0.1994952	0.3517234	0.2809699	0.6437428	0.663
C H	0.5539936	0.9185227	-0.4119378	0.488376	0.9659313	1.030278	0.348
D H	0.4400228	0.9008876	-0.2379384	0.4876893	0.6779612	1.012694	0.503

Appendix 10. Combined sensitivity-analysis league table

a. Sensitivity-analysis league table using r = 0.3							
AT							
-0.59 (-1.15,-0.03)	RT						
0.06 (-0.49,0.62)	0.65 (0.03,1.27)	CT					
0.14 (-0.40,0.68)	0.73 (0.02,1.44)	0.08 (-0.63,0.79)	IT				
-0.19 (-1.34,0.97)	0.40 (-0.81,1.61)	-0.25 (-1.46,0.97)	-0.33 (-1.55,0.90)	WBVT			
-0.47 (-1.99,1.05)	0.12 (-1.44,1.69)	-0.53 (-2.09,1.04)	-0.61 (-2.18,0.97)	-0.28 (-2.13,1.57)	IHT		
-0.04 (-0.91,0.83)	0.55 (-0.45,1.54)	-0.10 (-1.09,0.89)	-0.18 (-1.20,0.83)	0.14 (-1.29,1.58)	0.42 (-1.32,2.17)	ST	
-0.83 (-1.17,-0.49)	-0.24 (-0.74,0.26)	-0.89 (-1.39,-0.39)	-0.97 (-1.50,-0.43)	-0.64 (-1.75,0.46)	-0.36 (-1.84,1.12)	-0.79 (-1.70,0.13)	CON
b. Sensitivity-analysis league table using r = 0.7							
AT							
-0.49 (-1.03,0.05)	RT						
0.09 (-0.44,0.62)	0.58 (-0.03,1.18)	CT					
0.17 (-0.35,0.70)	0.66 (-0.03,1.36)	0.09 (-0.61,0.78)	IT				
-0.09 (-1.21,1.02)	0.40 (-0.78,1.57)	-0.18 (-1.35,0.99)	-0.27 (-1.46,0.92)	WBVT			
-0.43 (-1.82,0.96)	0.06 (-1.37,1.50)	-0.52 (-1.95,0.92)	-0.60 (-2.05,0.85)	-0.33 (-2.06,1.39)	IHT		
-0.07 (-0.89,0.75)	0.42 (-0.51,1.36)	-0.15 (-1.09,0.78)	-0.24 (-1.21,0.73)	0.03 (-1.34,1.40)	0.36 (-1.24,1.97)	ST	
-0.78 (-1.11,-0.45)	-0.29 (-0.77,0.19)	-0.87 (-1.34,-0.39)	-0.95 (-1.48,-0.42)	-0.68 (-1.75,0.38)	-0.35 (-1.70,1.00)	-0.71 (-1.58,0.15)	CON
c. Sensitivity-analysis league table after exclusion of Liu 2025							

AT							
-0.27 (-0.76,0.22)	RT						
-0.16 (-0.64,0.33)	0.12 (-0.46,0.69)	CT					
0.20 (-0.25,0.65)	0.47 (-0.14,1.09)	0.36 (-0.25,0.97)	IT				
-0.13 (-1.10,0.85)	0.14 (-0.89,1.17)	0.03 (-1.00,1.06)	-0.33 (-1.36,0.70)	WBVT			
-0.40 (-1.70,0.89)	-0.13 (-1.46,1.21)	-0.25 (-1.58,1.09)	-0.60 (-1.94,0.73)	-0.27 (-1.84,1.29)	IHT		
-0.04 (-0.79,0.70)	0.23 (-0.63,1.09)	0.11 (-0.74,0.96)	-0.25 (-1.11,0.62)	0.09 (-1.13,1.30)	0.36 (-1.13,1.84)	ST	
-0.77 (-1.06,-0.47)	-0.49 (-0.93,-0.05)	-0.61 (-1.05,-0.17)	-0.97 (-1.41,-0.52)	-0.64 (-1.57,0.29)	-0.36 (-1.62,0.90)	-0.72 (-1.51,0.06)	CON

Appendix 11. Forest plots of eligible comparisons of outcome indicator.

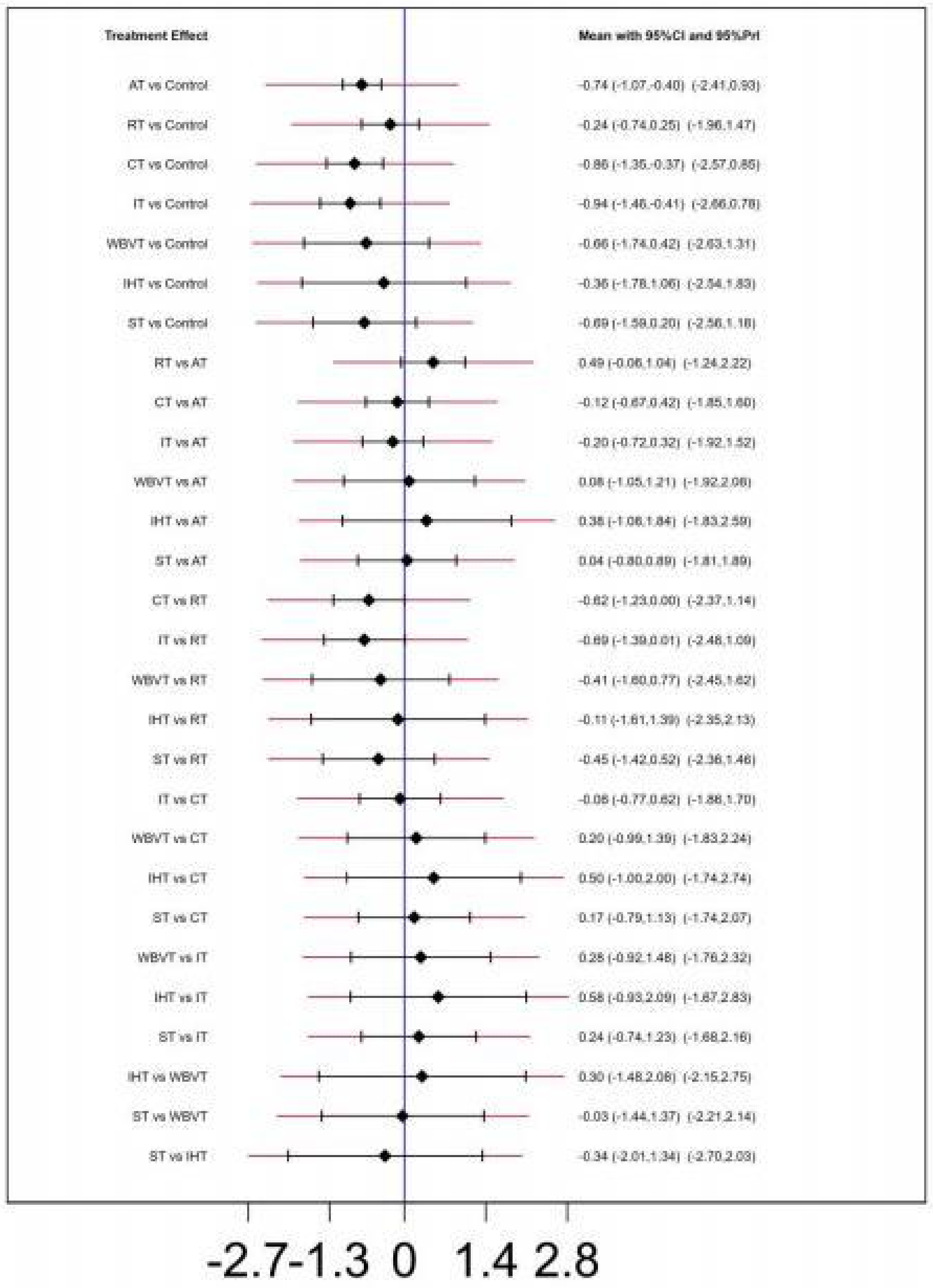

Appendix 12. Sensitivity analysis of SUCRA rankings

Treatment	r = 0.3			r = 0.5			r = 0.7			Excluding Liu 2025		
	SUCRA	PrBest (%)	Mean rank	SUCRA	PrBest (%)	Mean rank	SUCRA	PrBest (%)	Mean rank	SUCRA	PrBest (%)	Mean rank
CON	9.4	0	7.3	9.4	0	7.3	8.3	0	7.4	6.2	0	7.6
AT	65.6	4.8	3.4	61.6	2.6	3.7	63.5	3.3	3.6	66.9	4.6	3.3
RT	25	0.1	6.3	25.7	0.1	6.2	26.6	0.2	6.1	40.1	1.5	5.2
CT	71.1	16.9	3	72.7	19.2	2.9	70.7	16.2	3.1	50.9	3.9	4.4
IT	76.8	27.4	2.6	77.9	28	2.5	78.2	30.3	2.5	84.3	40.2	2.1
WBVT	52.5	17.9	4.3	55.7	20	4.1	56.7	21.3	4	53.8	18	4.2
IHT	38.2	13.2	5.3	38.9	13.7	5.3	38.2	12.2	5.3	36.7	12.6	5.4
ST	61.4	19.8	3.7	58.1	16.5	3.9	57.9	16.4	3.9	61.1	19.2	3.7

Appendix 13. Key assumptions of Network Meta-Analysis

There are three key assumptions underpinning the validity of network meta-analysis (NMA): network connectivity, transitivity, and consistency(Donegan et al. 2013; Watt et al. 2019).

1. Connectivity

Connectivity is a key assumption in NMA which if deemed insufficient (i.e., due to lack of direct comparators) can lead to low statistical power and misleading results (Ter Veer et al. 2019). In our study, we assessed connectivity of the network at both treatment and agent levels visually and found no evidence of unconnectedness on either network (Appendix 13-1 and Appendix 13-2).

Appendix 13-1 Treatment-level network.

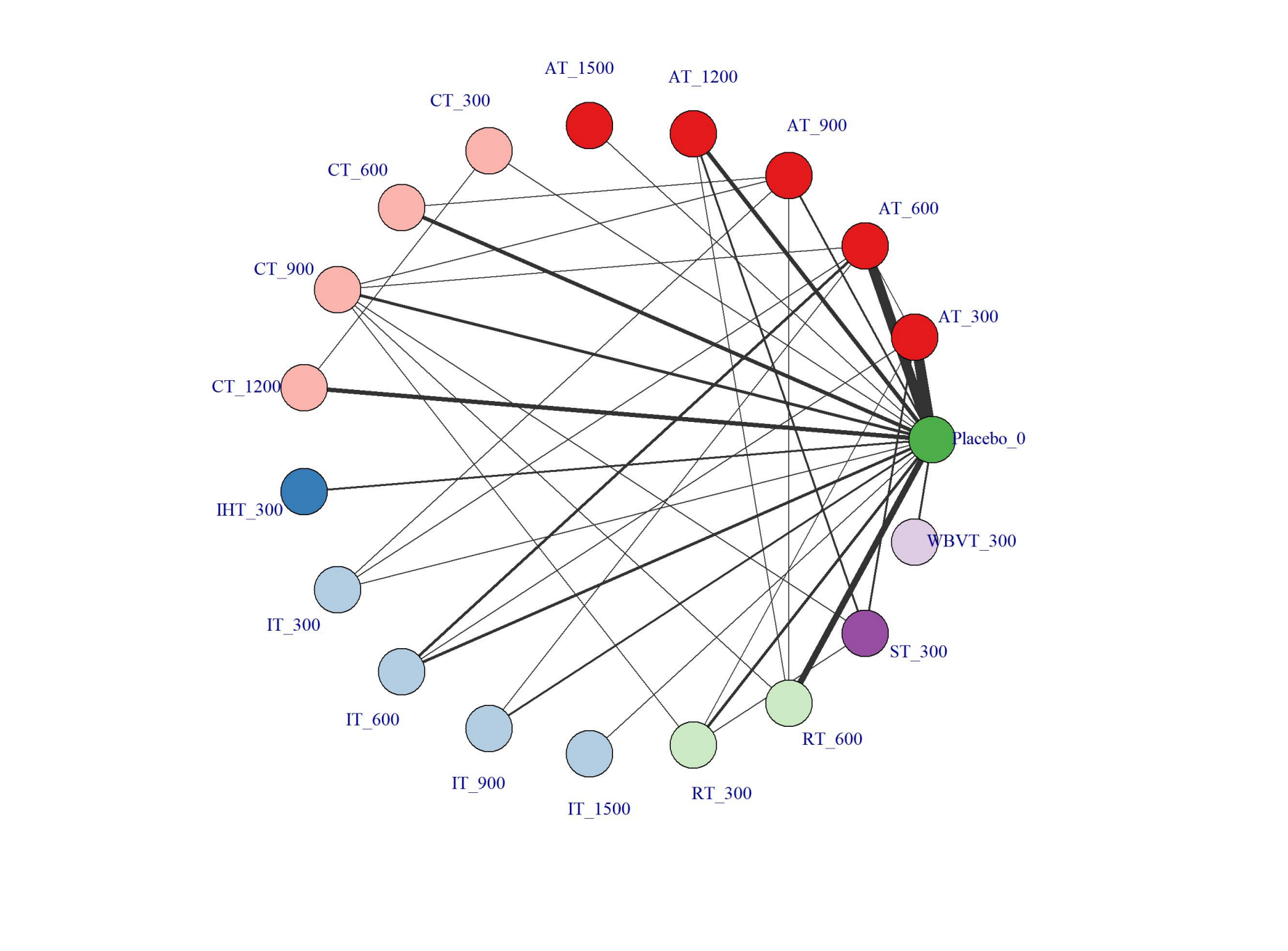

Appendix 13-2 Agent-level network.

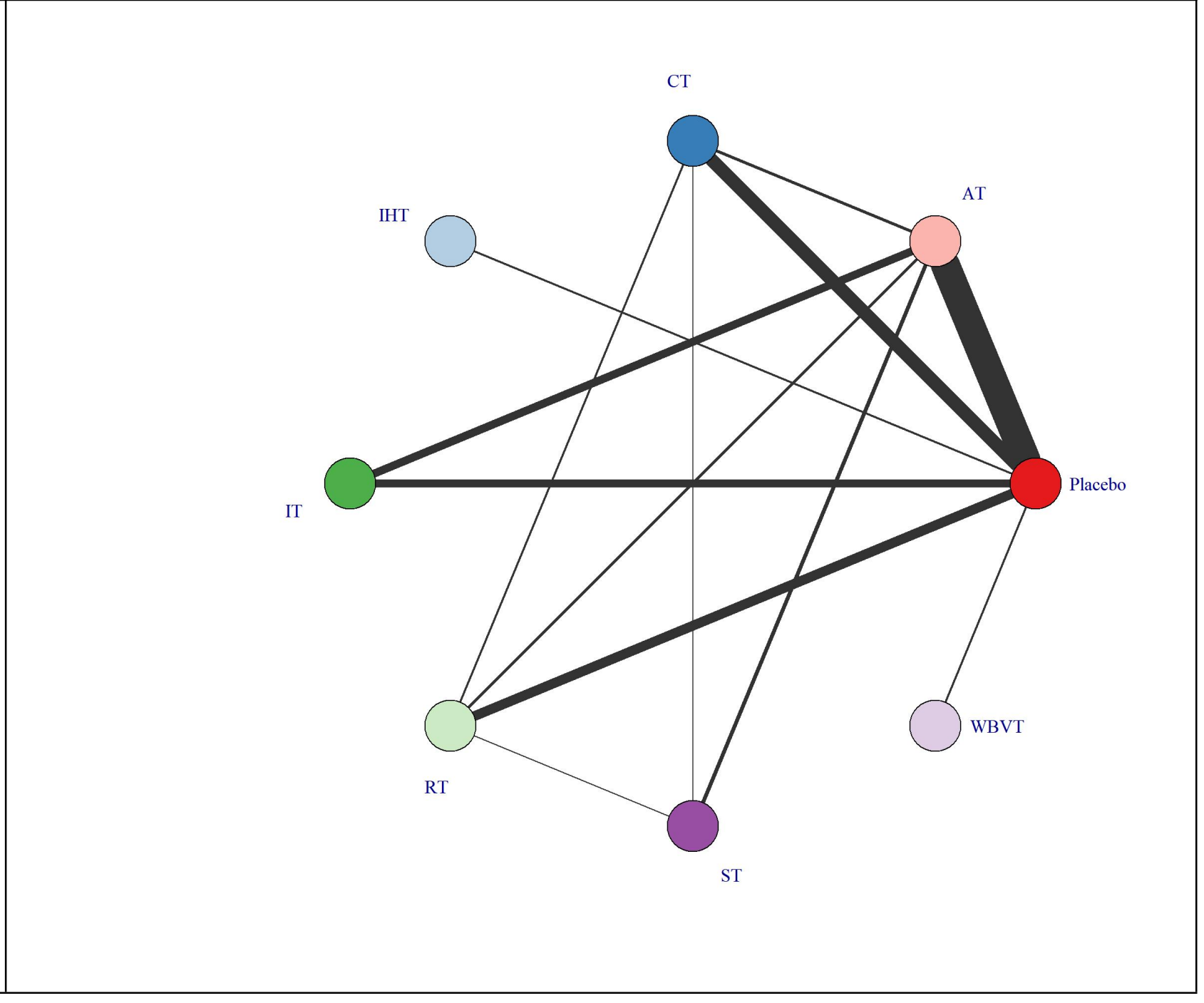

The first value indicates the specific exercise modality and the second indicates the corresponding dose category (MET·min/week) used for visualization. CON, non-exercise control; AT, aerobic training; RT, resistance training; CT, combined training; IT, interval training; WBVT, whole-body vibration training; IHT, isometric handgrip training; ST, stretching training.

2. Transitivity

The validity of indirect comparisons in NMA relies on the assumption of transitivity, which requires that the included comparisons are sufficiently similar with respect to important effect modifiers. In this study, transitivity was assessed qualitatively by comparing the distribution of key potential effect modifiers across intervention nodes, including age, baseline cfPWV, and BMI. The corresponding distributions are shown in Appendix 13-3. Overall, the main intervention nodes showed broadly overlapping distributions, although some degree of clinical heterogeneity remained, particularly in sparsely informed nodes such as WBVT, IHT, and ST.

Appendix 13-3. Distribution of key potential effect modifiers across intervention nodes.

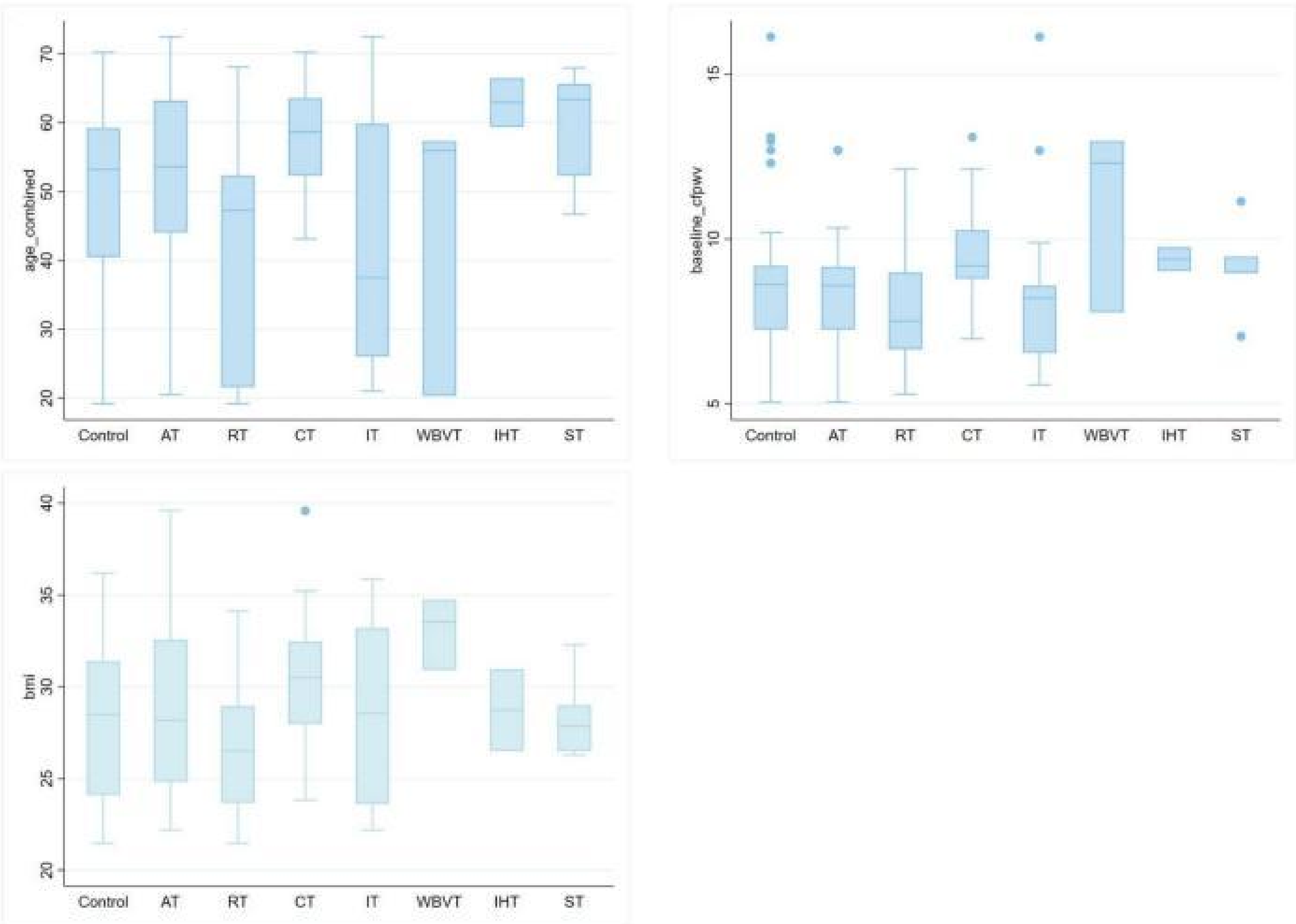

3. Consistency

Consistency refers to agreement between direct and indirect evidence within the network. In the present study, consistency was evaluated using both model-level comparison and local inconsistency assessment. First, consistency and unrelated mean effects (UME) models were compared in terms of pV, residual deviance, and Deviance Information Criterion (DIC) (Wheeler et al. 2010). Similar values between the two models indicated acceptable overall consistency of the network (Appendix 13-4). Second, local consistency was evaluated using node-splitting analysis, which separates direct and indirect evidence for specific treatment contrasts and tests for disagreement between them (van Valkenhoef et al. 2016) (Appendix 13-5).

Appendix 13-4. Consistency and UME models fit comparison.

Model	pV	Residual deviance	DIC
Consistency	132.9	135.6	224.8
UME	133.6	135.2	225.1

DIC: Deviance Information Criterion; pV: effective number of parameters reported in the current JAGS output; UME: unrelated mean effects. Similar values of DIC and residual deviance between the consistency and UME models indicate acceptable consistency of the network.

Appendix 13-5 Node-splitting analysis (density plot).

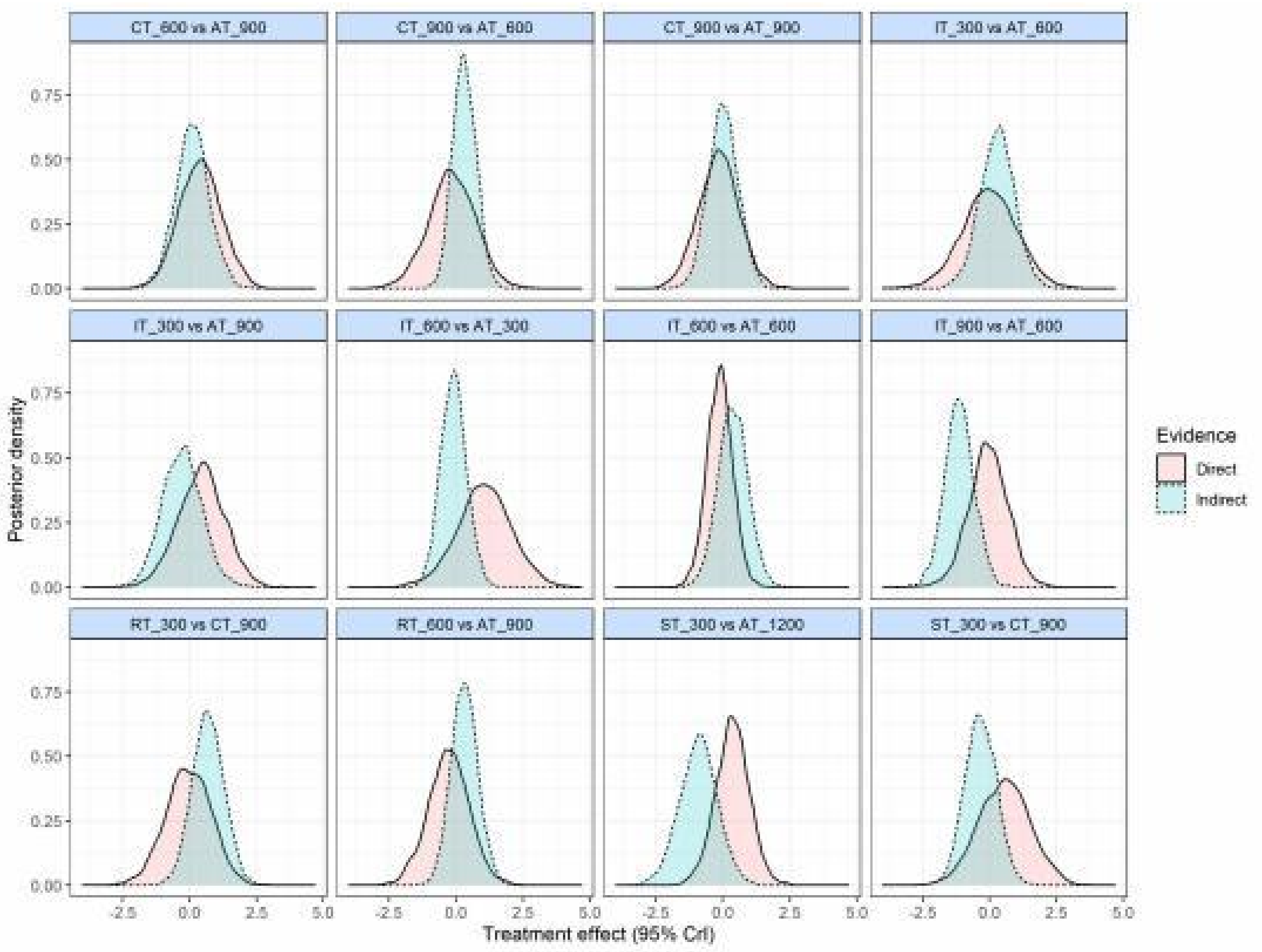

Appendix 14. Model selection for the dose-response network meta-analysis

A meta-analysis (i.e., a "split" NMA) of the different doses of physical activity as separate and unrelated treatments were performed. This step helps determine which function is more appropriate for the data and should be used in a model-based network meta-analysis (MBNMA) (Mawdsley et al. 2016).

1. Models selection

Appendix 14-1 Models fit comparison

Model	DIC	Residual deviance	pD
Emax (common treatment effects)	655.51	638.2	65.48
Restricted cubic spline	206.09	134.48	109.75
Quadratic function (random treatment effects)	202.34	133.38	108.44

In the present dataset, the quadratic model yielded the lowest DIC (202.34), whereas the restricted cubic spline model showed a similar fit (DIC = 206.09). Because the difference in DIC was small, these two models were considered broadly comparable in fit. The quadratic model was retained for subsequent analyses because it provided a slightly better fit together with greater parsimony and easier clinical interpretation.

2. Deviation check

In addition to the model fit index, a deviation plot showing the contribution of each data point to the residuals can also help to confirm the robustness of the model selection. The contribution of each data point to the posterior mean bias should be around 1, which indicates a good model fit [7]. Deviation plots for overall (Appendix 14-2) treatment effects (Appendix 14-3) confirm the robustness of our model selection (i.e., deviations <1.5).

Appendix 14-2 Deviance plot at overall exercise

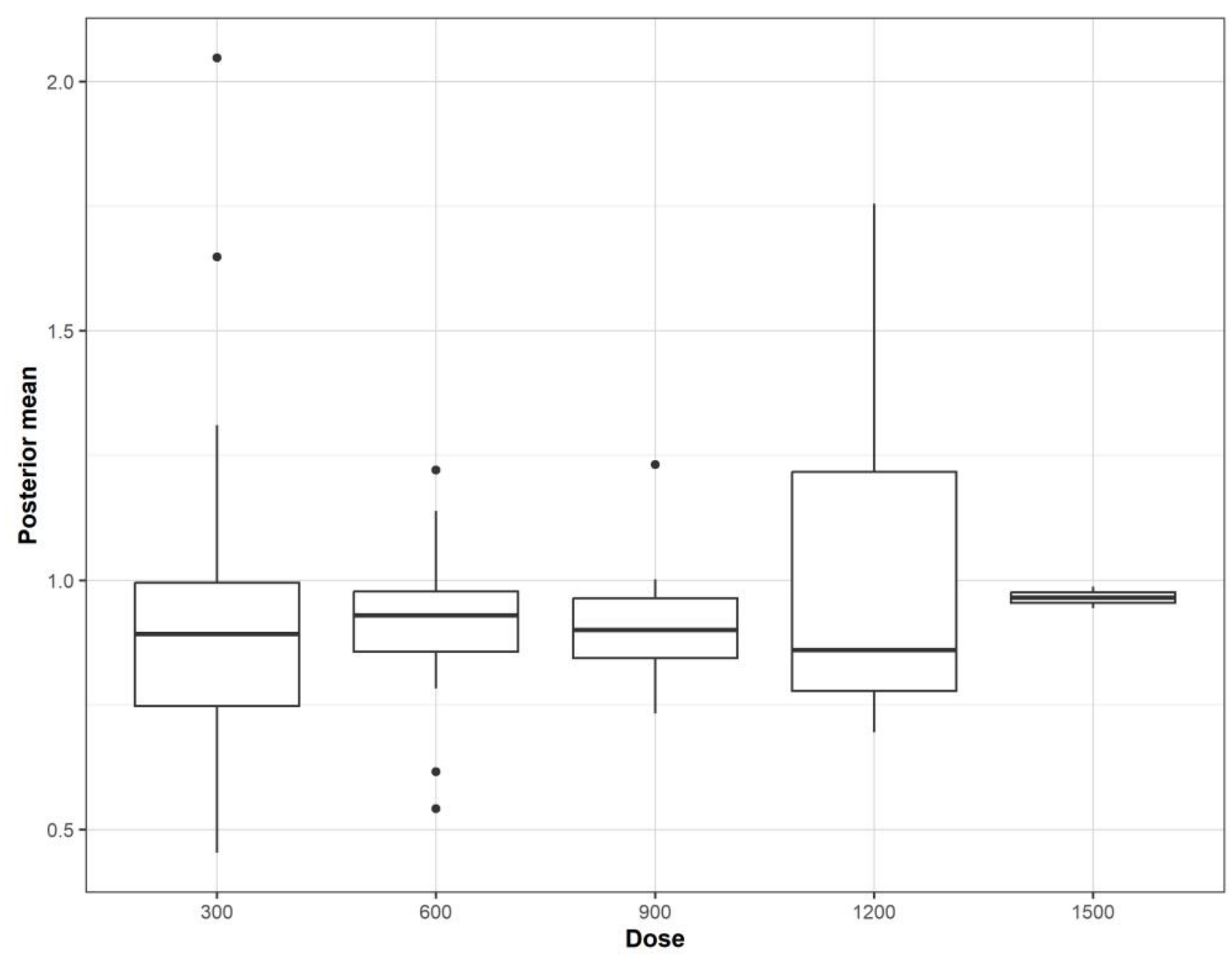

Appendix 14-3 Deviance plots at treatment-level

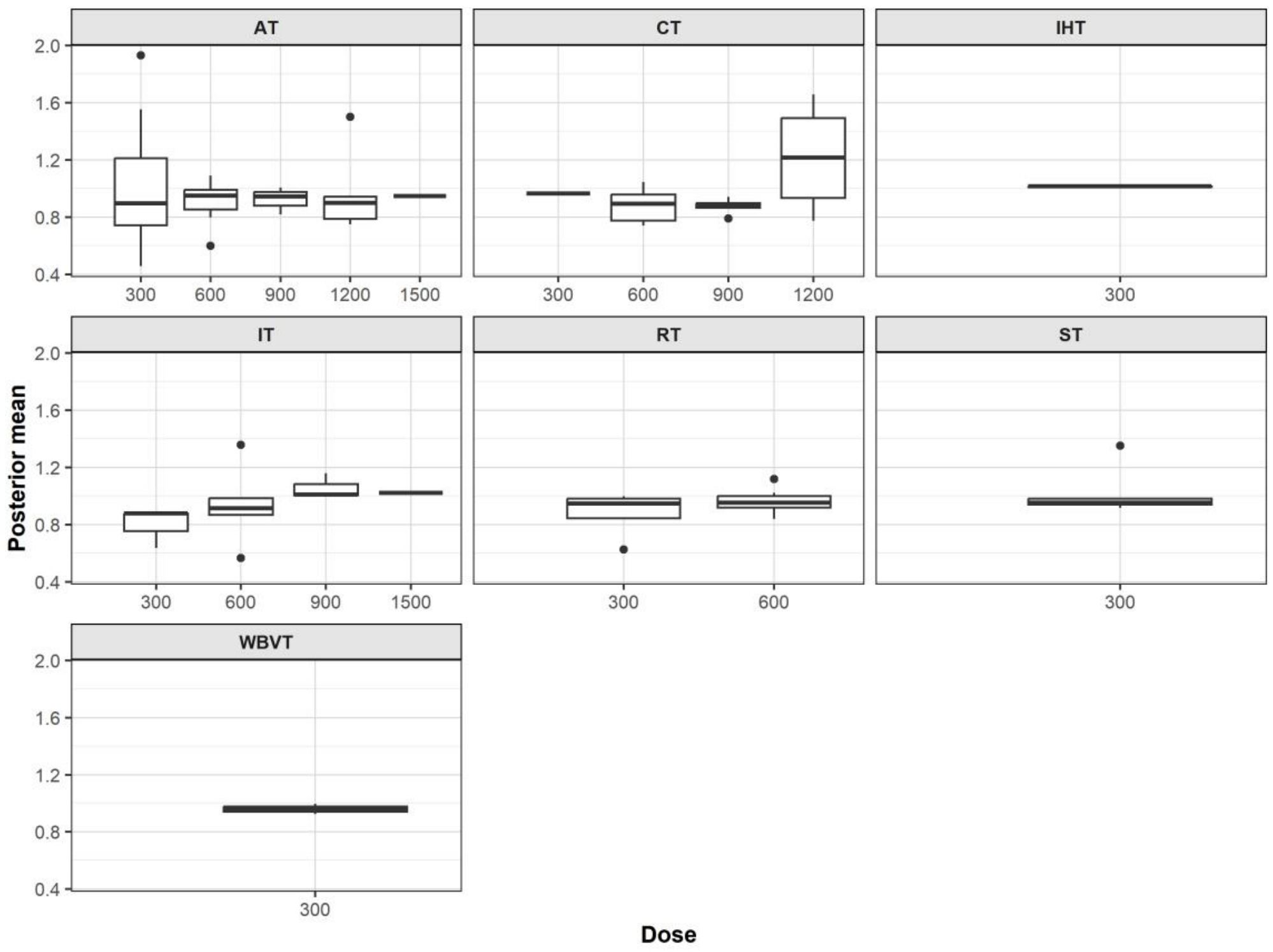

3. Degree of fit test

In addition, we also plotted the fit further to assess the degree of fit of the model. The fit values are plotted as connecting lines, and the observations in the original dataset are plotted as points. These plots can determine if the model fits the data well for different exercises and doses of the dose-response function(*Dose-Response Functions*, n.d.).

Appendix 14-4 Fit plots at overall exercise level.

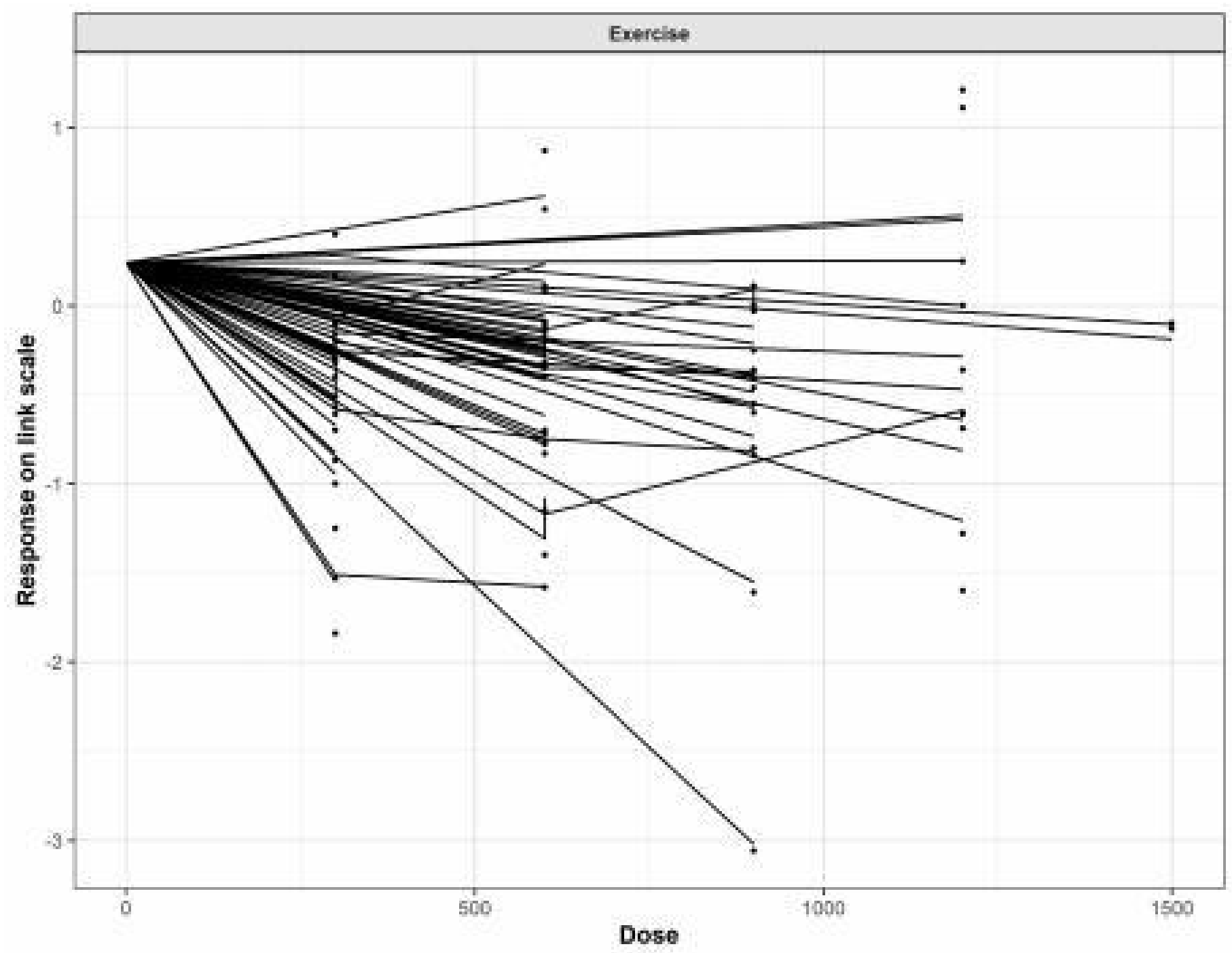

Appendix 14-5 Fit plots at agent-level.

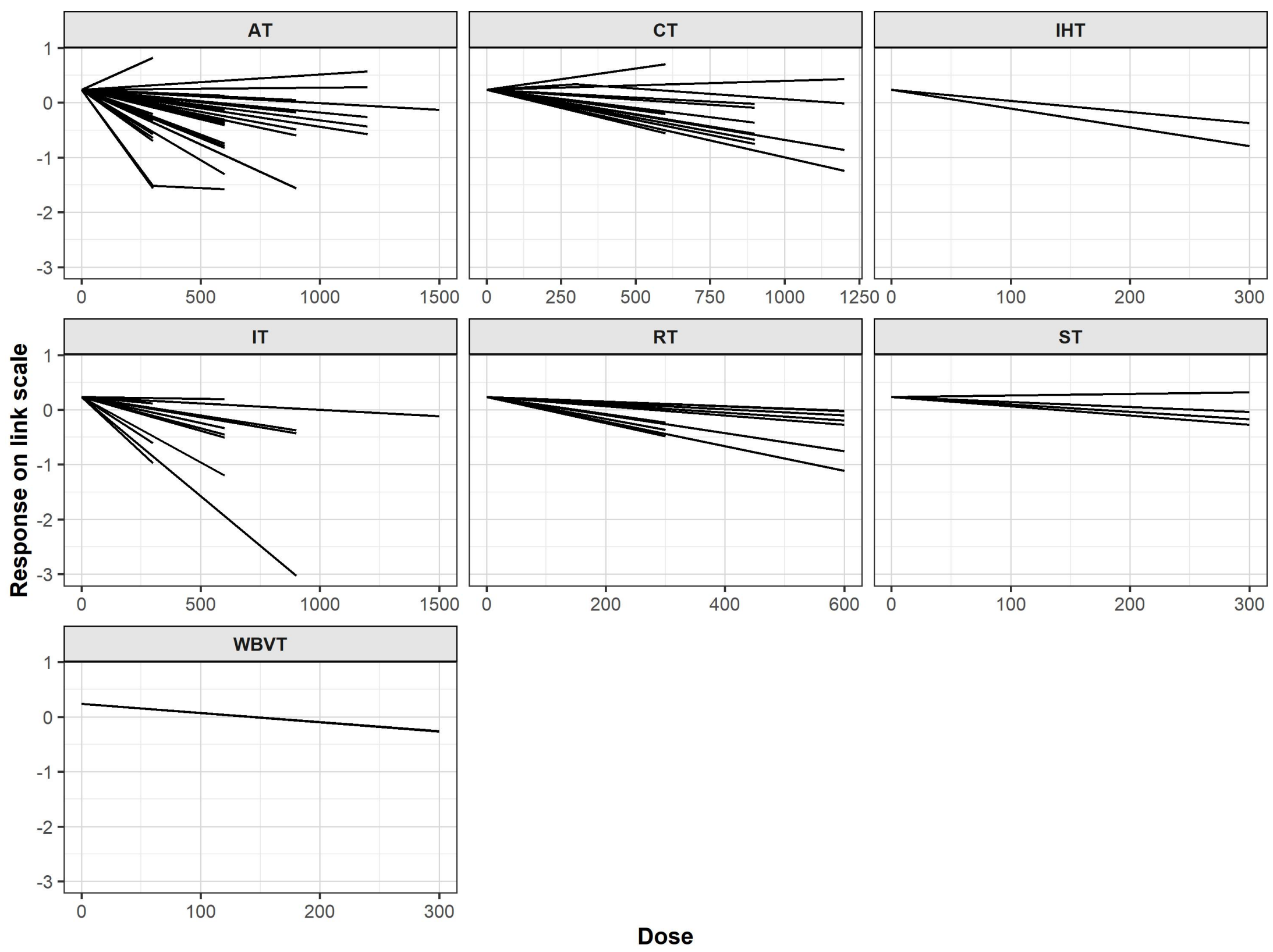

Appendix 15. Exercise Dose and carotid–femoral pulse wave velocity Dose-Response Relationships

Appendix 15-1 Overall Exercise Dose and cfPWV Dose-Response Relationships

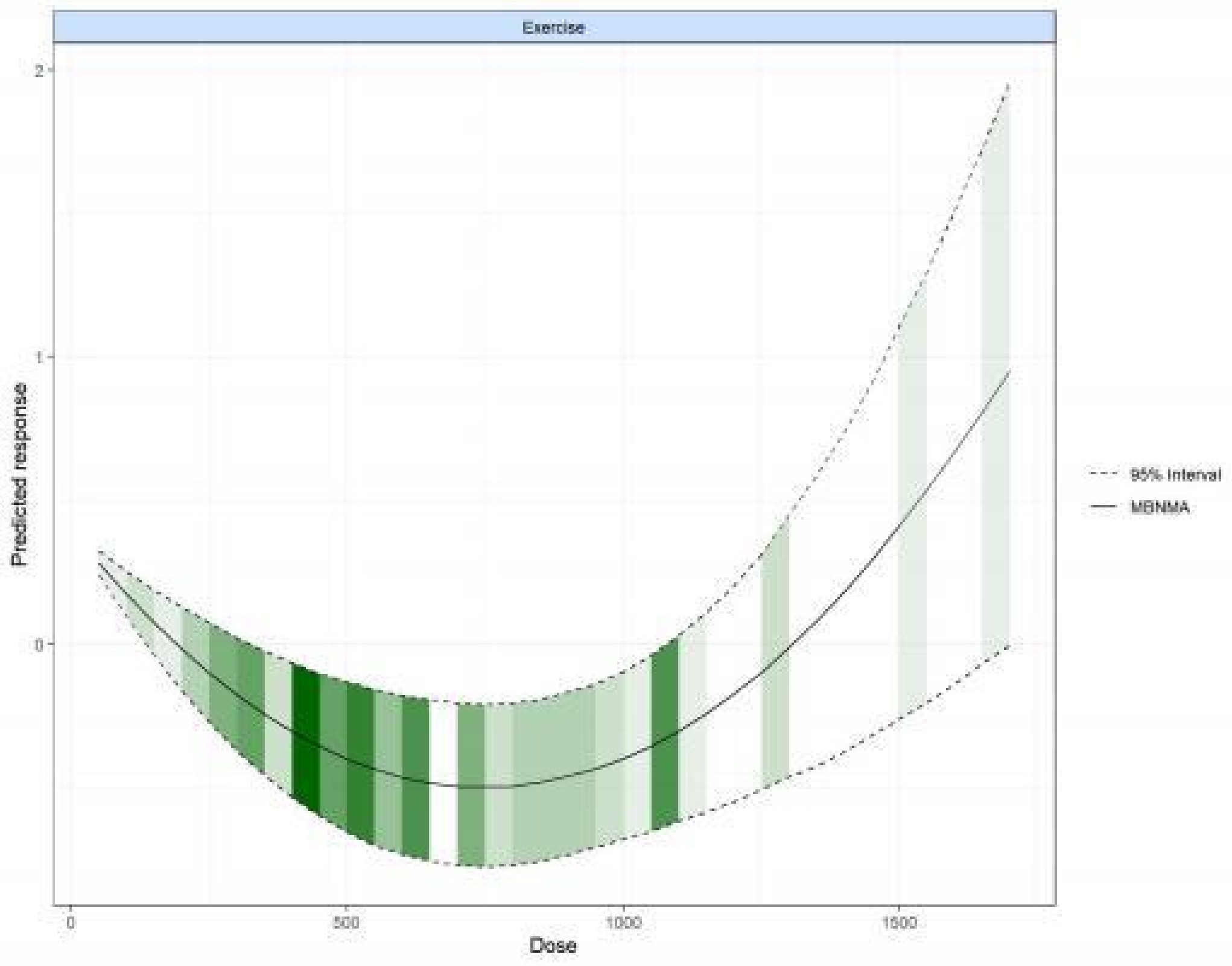

Appendix 15-2 Dose-Response Relationships of Dose and cfPWV by Exercise Modality

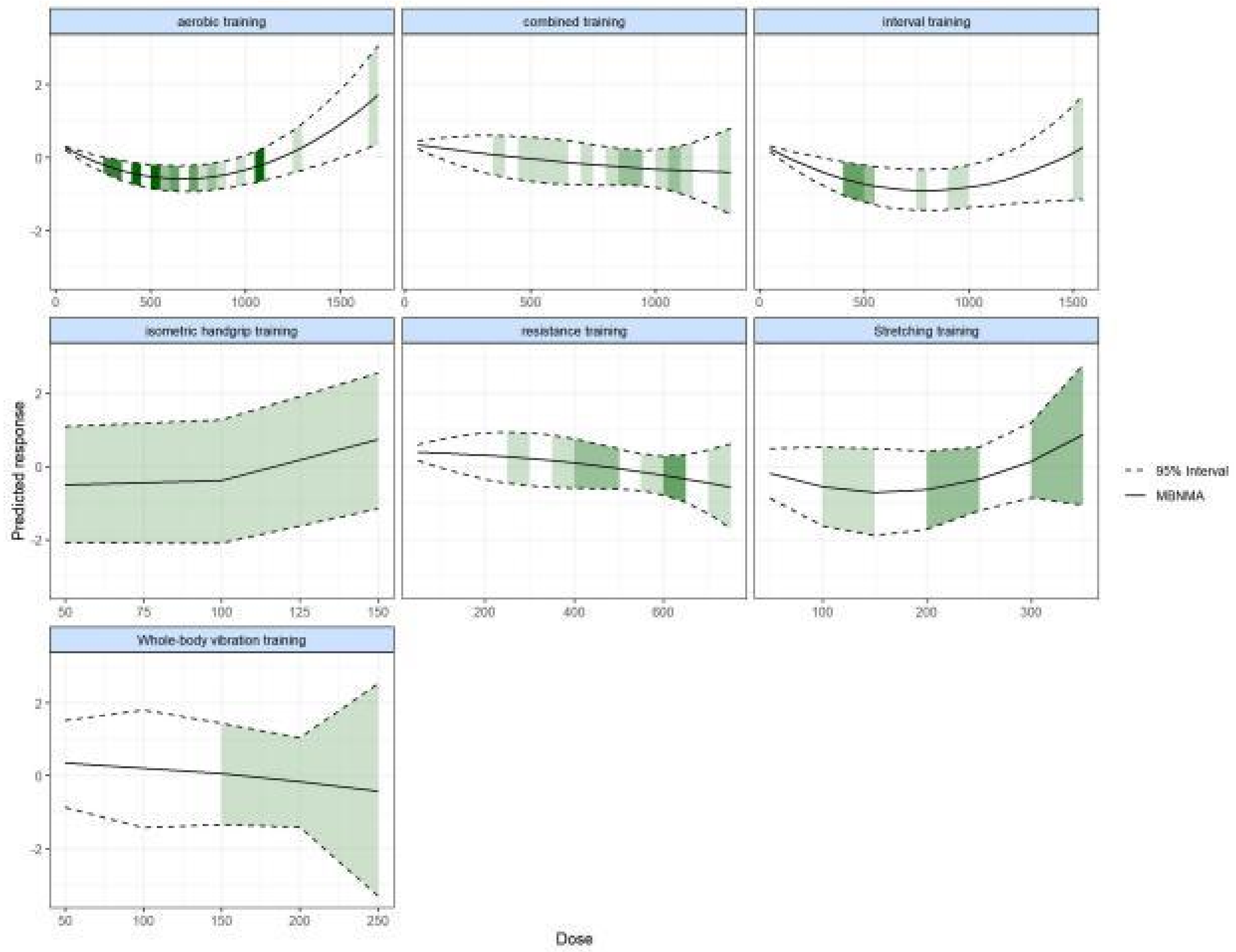

Dose-response relationship between exercise dose and cfPWV. The green shaded area in the figure indicates the original study dataset; the darker the color, the larger the amount of data.

Appendix 16. Ranking of exercise modality-dose combinations

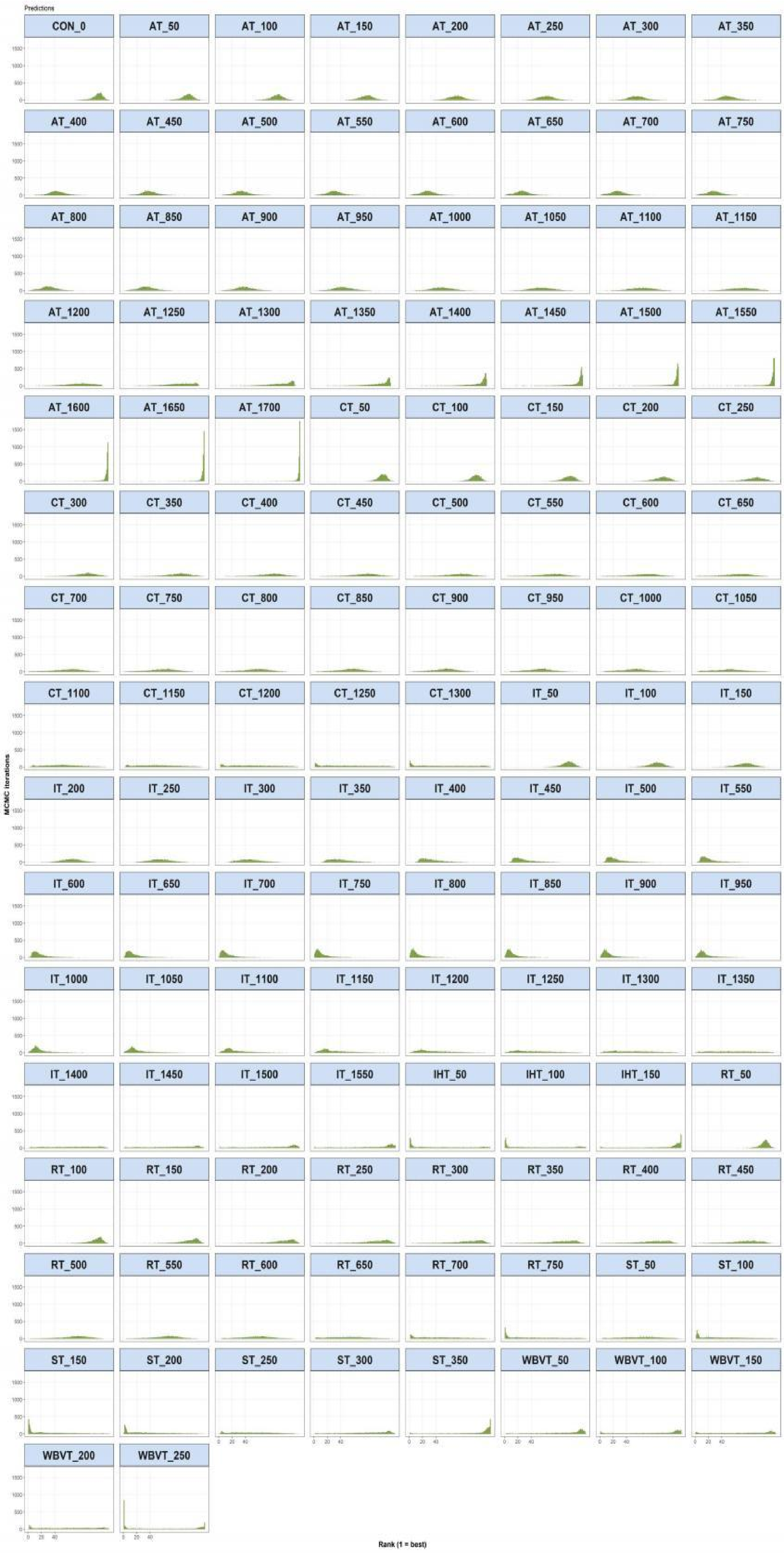
Predictions
CON_0
AT_50
AT_100
AT_150
AT_200
AT_250
AT_300
AT_350
AT_400
AT_450
AT_500
AT_550
AT_600
AT_650
AT_700
AT_750
AT_800
AT_850
AT_900
AT_950
AT_1000
AT_1050
AT_1100
AT_1150
AT_1200
AT_1250
AT_1300
AT_1350
AT_1400
AT_1450
AT_1500
AT_1550
AT_1600
AT_1650
AT_1700
CT_50
CT_100
CT_150
CT_200
CT_250
CT_300
CT_350
CT_400
CT_450
CT_500
CT_550
CT_600
CT_650
CT_700
CT_750
CT_800
CT_850
CT_900
CT_950
CT_1000
CT_1050
CT_1100
CT_1150
CT_1200
CT_1250
CT_1300
IT_50
IT_100
IT_150
IT_200
IT_250
IT_300
IT_350
IT_400
IT_450
IT_500
IT_550
IT_600
IT_650
IT_700
IT_750
IT_800
IT_850
IT_900
IT_950
IT_1000
IT_1050
IT_1100
IT_1150
IT_1200
IT_1250
IT_1300
IT_1350
IT_1400
IT_1450
IT_1500
IT_1550
IHT_50
IHT_100
IHT_150
RT_50
RT_100
RT_150
RT_200
RT_250
RT_300
RT_350
RT_400
RT_450
RT_500
RT_550
RT_600
RT_650
RT_700
RT_750
ST_50
ST_100
ST_150
ST_200
ST_250
ST_300
ST_350
WBVT_50
WBVT_100
WBVT_150
WBVT_200
WBVT_250
MCMC Iterations
Rank (1 = best)

The first value indicates the specific intervention and the second one is the corresponding dose of that intervention (MET·min/week).

Appendix 17. GRADE assessment for comparisons with direct evidence in the network meta-analysis.

Comparison	k	Direct WMD [95% CI]	Quality of evidence	Indirect WMD [95% CI]	Quality of evidence	Network WMD [95% CI]	Quality of evidence	P-value
AT vs CON	27	-0.85 (-1.19, -0.52)	Low ‡*	-0.63 (-1.35, 0.09)	Very low †*	-0.74 (-1.07, -0.40)	Low ‡*	0.737
RT vs CON	11	-0.36 (-0.73, 0.01)	Low †*	0.07 (-0.87, 1.00)	Very low †‡*	-0.24 (-0.74, 0.25)	Low †*	0.444
CT vs CON	12	-0.49 (-0.78, -0.20)	Moderate *	-1.31 (-2.11, -0.50)	Low †‡*	-0.86 (-1.35, -0.37)	Moderate *	0.17
IT vs CON	7	-1.19 (-2.55, 0.18)	Very low †‡*	-0.55 (-1.38, 0.28)	Very low †‡*	-0.94 (-1.46, -0.41)	Very low †‡*	0.236
WBVT vs CON	3	-0.40 (-0.64, -0.16)	Moderate *	NA	NA	-0.66 (-1.74, 0.42)	Low †*	NA
IHT vs CON	2	-0.36 (-1.61, 0.88)	Very low †‡*	NA	NA	-0.36 (-1.78, 1.06)	Very low †‡*	NA
AT vs RT	4	0.27 (-0.20, 0.74)	Low †*	-0.81 (-1.46, -0.16)	Low †‡*	-0.49 (-1.04, 0.06)	Low †*	0.077
AT vs CT	3	-0.07 (-0.44, 0.30)	Low †*	0.20 (-0.45, 0.84)	Very low †‡*	0.12 (-0.42, 0.67)	Low †*	0.68
AT vs IT	8	0.02 (-0.15, 0.19)	Low †*	0.57 (-0.26, 1.39)	Very low †‡*	0.20 (-0.32, 0.72)	Low †*	0.261
AT vs ST	4	0.15 (-0.48, 0.78)	Low †*	-0.79 (-2.65, 1.08)	Very low †‡*	-0.04 (-0.89, 0.80)	Low †*	0.38
CT vs RT	3	-1.17 (-3.99, 1.66)	Very low †‡*	-0.20 (-0.98, 0.57)	Very low †*	-0.62 (-1.23, 0.00)	Very low †‡*	0.09
CT vs ST	1	-0.54 (-1.76, 0.68)	Very low †‡*	-0.10 (-1.19, 0.99)	Very low †*	-0.17 (-1.13, 0.79)	Very low †‡*	0.785
RT vs ST	1	-0.60 (-1.86, 0.66)	Very low †‡*	0.75 (-0.34, 1.83)	Very low †*	0.45 (-0.52, 1.42)	Very low †‡*	0.243

k = number of studies providing direct evidence. Network WMD = estimated treatment effect (WMD, m/s) derived from the network meta-analysis. Direct WMD = estimated treatment effect (WMD, m/s) derived from direct evidence. Indirect WMD = estimated treatment effect (WMD, m/s) derived from indirect evidence. P = p-value of the test for disagreement between direct and indirect evidence. AT, aerobic training; RT, resistance training; CT, combined training; IT, interval training; ST, stretching training; IHT, isometric handgrip training; WBVT, whole-body vibration training; CON, non-exercise control; NA, not available. Lower cfPWV values indicate a more favourable effect.

Reasons for downgrading: † imprecision (one-level downgrade when the 95% confidence interval crossed the null value); ‡ inconsistency (one-level downgrade for substantial heterogeneity in direct evidence or loop-specific inconsistency); * study limitations/risk of bias (one-level downgrade when studies with some concerns or high risk of bias accounted for ≥25% of the direct evidence); ^ indirectness (one-level downgrade).

The p-value indicated that direct and indirect estimates were not statistically different; therefore, no comparison was downgraded specifically because of direct–indirect disagreement.

References

A. Correia M, P. L. Oliveira, B. Q. Farah, et al. 2020. "Effects of Isometric Handgrip Training in Patients With Peripheral Artery Disease: A Randomized Controlled Trial." *Journal of the American Heart Association* 9 (4): e013596. https://doi.org/10.1161/JAHA.119.013596.

Alvarez-Alvarado, S., S. J. Jaime, M. J. Ormsbee, et al. 2017. "Benefits of Whole-Body Vibration Training on Arterial Function and Muscle Strength in Young Overweight/Obese Women." *Hypertension Research : Oficial Journal ofthe Japanese Society ofHypertension* 40 (5): 487–92. https://doi.org/10.1038/hr.2016.178.

Banks, N. F., E. M. Rogers, A. E. Stanhewicz, K. M. Whitaker, and N. D. M. Jenkins. 2024. "Resistance Exercise Lowers Blood Pressure and Improves Vascular Endothelial Function in Individuals with Elevated Blood Pressure or Stage-1 Hypertension." *American Journal of Physiology. Heart and Circulatory Physiology* 326 (1): H256-H269. https://doi.org/10.1152/ajpheart.00386.2023.

Baynard, T., G. J. Griffith, S. O. Wee, et al. 2023. "Home-Based Exercise Improves Subclinical Atherosclerosis Marker in Multiple Sclerosis." *Multiple Sclerosis and Related Disorders* 79. https://doi.org/10.1016/j.msard.2023.105002.

Blumenthal, J. A., A. L. Hinderliter, P. J. Smith, et al. 2021. "Effects of Lifestyle Modification on Patients With Resistant Hypertension: Results of the TRIUMPH Randomized Clinical Trial." *Circulation* 144 (15): 1212-1226. https://doi.org/10.1161/CIRCULATIONAHA.121.055329.

Brubaker, P. H., B. J. Nicklas, D. K. Houston, et al. 2023. "A Randomized, Controlled Trial of Resistance Training Added to Caloric Restriction Plus Aerobic Exercise Training in Obese Heart Failure with Preserved Ejection Fraction." *Circulation. Heart Failure* 16 (2): e010161. https://doi.org/10.1161/CIRCHEARTFAILURE.122.010161.

Chang, C. R., L. A. Roach, B. M. Russell, and M. E. Francois. 2025. "Using Continuous Glucose Monitoring to Prescribe an Exercise Time: A Randomised Controlled Trial in Adults with Type 2 Diabetes." *Diabetes Research and Clinical Practice* 222: 112072. https://doi.org/10.1016/j.diabres.2025.112072.

Choo, J., J. Lee, J. H. Cho, L. E. Burke, A. Sekikawa, and S. Y. Jae. 2014. "Effects of Weight Management by Exercise Modes on Markers of Subclinical Atherosclerosis and Cardiometabolic Profile among Women with Abdominal Obesity: A Randomized Controlled Trial." *BMC Cardiovascular Disorders* 14: 82. https://doi.org/10.1186/1471-2261-14-82.

Chrysohoou, C., A. Angelis, G. Tsitsinakis, et al. 2015. "Cardiovascular Effects of High-Intensity Interval Aerobic Training Combined with Strength Exercise in Patients with Chronic Heart Failure. A Randomized Phase III Clinical Trial." *International Journal of Cardiology* 179: 269-274. https://doi.org/10.1016/j.ijcard.2014.11.067.

Ciolac, E. G., E. A. Bocchi, L. A. Bortolotto, V. O. Carvalho, J. M. Greve, and G. V. Guimarães. 2010. "Effects of High-Intensity Aerobic Interval Training vs. Moderate Exercise on Hemodynamic, Metabolic and Neuro-Humoral Abnormalities of Young Normotensive Women at High Familial Risk for Hypertension." *Hypertension Research* 33 (8): 836-843. https://doi.org/10.1038/hr.2010.72.

Cooke, A. B., V. Ta, S. Iqbal, et al. 2018. "The Impact of Intradialytic Pedaling Exercise on Arterial Stiffness: A Pilot Randomized Controlled Trial in a Hemodialysis Population." *American Journal ofHypertension* 31 (4): 458-466. https://doi.org/10.1093/ajh/hpx191.

Cortez-Cooper, M. Y., M. M. Anton, A. E. Devan, D. B. Neidre, J. N. Cook, and H. Tanaka. 2008. "The Effects of Strength Training on Central Arterial Compliance in Middle-Aged and Older Adults." *European Journal of Cardiovascular Prevention and Rehabilitation* 15 (2): 149-155. https://doi.org/10.1097/HJR.0b013e3282f02fe2.

Cox, E. R., T. Gajanand, S. E. Keating, et al. 2024. "Effect of Low-Volume Combined Aerobic and Resistance High-Intensity Interval Training on Vascular Health in People with Type 2 Diabetes: A Randomised Controlled Trial." *European Journal ofApplied Physiology* 124 (9): 2819-2833. https://doi.org/10.1007/s00421-024-05473-8.

Croymans, D. M., S. L. Krell, C. S. Oh, et al. 2014. "Effects of Resistance Training on Central Blood Pressure in Obese Young Men." *Journal ofHuman Hypertension* 28 (3): 157-164. https://doi.org/10.1038/jhh.2013.81.

Deiseroth, Arne, Lukas Streese, Sabrina Köchli, et al. 2019. "Exercise and Arterial Stiffness in the Elderly: A Combined Cross-Sectional and Randomized Controlled Trial (EXAMIN AGE)." *Frontiers in Physiology* Volume 10-2019. https://doi.org/10.3389/fphys.2019.01119.

DeVallance, E., S. Fournier, K. Lemaster, et al. 2016. "The Effects of Resistance Exercise Training on Arterial Stiffness in Metabolic Syndrome." *European Journal ofApplied Physiology* 116 (5): 899-910. https://doi.org/10.1007/s00421-016-3348-4.

Dobrosielski, D. A., B. B. Gibbs, P. Ouyang, et al. 2012. "Effect of Exercise on Blood Pressure in Type 2 Diabetes: A Randomized Controlled Trial." *Journal of General Internal Medicine* 27 (11): 1453-1459. https://doi.org/10.1007/s11606-012-2103-8.

Donegan, Sarah, Paula Williamson, Umberto D'Alessandro, and Catrin Tudur Smith. 2013. "Assessing Key Assumptions of Network Meta-Analysis: A Review of Methods." *Research Synthesis Methods* (England) 4 (4): 291–323. https://doi.org/10.1002/jrsm.1085.

Donley, D. A., S. B. Fournier, B. L. Reger, et al. 2014. "Aerobic Exercise Training Reduces Arterial Stiffness in Metabolic Syndrome." *Journal ofApplied Physiology (Bethesda, Md.: 1985)* 116 (11): 1396-1404. https://doi.org/10.1152/japplphysiol.00151.2014.

Dose-Response Functions. n.d. Accessed August 21, 2024. https://cran.r-project.org/web/packages/MBNMAdose/vignettes/runmbnmadose-2.html.

Farah, B. Q., S. L. C. Rodrigues, G. O. Silva, et al. 2018. "Supervised, but Not Home-Based, Isometric Training Improves Brachial and Central Blood Pressure in Medicated Hypertensive Patients: A Randomized Controlled Trial." *Frontiers in Physiology* 9 (JUL): 961. https://doi.org/10.3389/fphys.2018.00961.

Fernandez-del-Valle, Maria, Joaquin U. Gonzales, Shelby Kloiber, Sunanda Mitra, Jon Klingensmith, and Eneko Larumbe-Zabala. 2018. "Effects of Resistance Training on MRI-Derived Epicardial Fat Volume and Arterial Stiffness in Women with Obesity: A Randomized Pilot Study." *European Journal ofApplied Physiology* 118 (6): 1231–40. https://doi.org/10.1007/s00421-018-3852-9.

Figueroa, A., R. Kalfon, T. A. Madzima, and A. Wong. 2014. "Whole-Body Vibration Exercise Training Reduces Arterial Stiffness in Postmenopausal Women with Prehypertension and Hypertension." *Menopause (New York, N.Y.)* 21 (2): 131-136. https://doi.org/10.1097/GME.0b013e318294528c.

Figueroa, A., R. Kalfon, and A. Wong. 2015. "Whole-Body Vibration Training Decreases Ankle Systolic Blood Pressure and Leg Arterial Stiffness in Obese Postmenopausal Women

with High Blood Pressure.” *Menopause (New York, N.Y.)* 22 (4): 423-427. https://doi.org/10.1097/GME.0000000000000332.

Fujie, S., K. Sanada, T. Hamaoka, and M. Iemitsu. 2022. “Time-Dependent Relationships between Exercise Training-Induced Changes in Nitric Oxide Production and Hormone Regulation.” *Experimental Gerontology* 166: 111888. https://doi.org/10.1016/j.exger.2022.111888.

Ghardashi-Afousi, Alireza, Linda S. Pescatello, Maryam Asvadi Fard, and Aida Sabouri. 2025. “Salusin-Beta, Arterial Stiffness, and Heart Rate Variability Influence the Blood Pressure Response to High-Intensity Interval Training Among Older Adults With Hypertension: A Randomized Control Trial.” *Health Science Reports* 8 (11): e71329–e71329. https://doi.org/10.1002/hsr2.71329.

Goldberg, M. J., S. H. Boutcher, and Y. N. Boutcher. 2012. “The Effect of 4 Weeks of Aerobic Exercise on Vascular and Baroreflex Function of Young Men with a Family History of Hypertension.” *Journal ofHuman Hypertension* 26 (11): 644–49. https://doi.org/10.1038/jhh.2011.95.

Gómez-Sánchez, L., M. Gómez-Sánchez, C. Lugones-Sánchez, et al. 2022. “Long-Term Effectiveness of a Smartphone App and a Smart Band on Arterial Stiffness and Central Hemodynamic Parameters in a Population with Overweight and Obesity (Evident 3 Study): Randomised Controlled Trial.” *Nutrients* 14 (22). https://doi.org/10.3390/nu14224758.

Greenwood, S. A., P. Koufaki, T. H. Mercer, R. Rush, et al. 2015. “Aerobic or Resistance Training and Pulse Wave Velocity in Kidney Transplant Recipients: A 12-Week Pilot Randomized Controlled Trial (the Exercise in Renal Transplant [ExeRT] Trial).” *American Journal of Kidney Diseases* 66 (4): 689-698. https://doi.org/10.1053/j.ajkd.2015.06.016.

Greenwood, S. A., P. Koufaki, T. H. Mercer, H. L. MacLaughlin, et al. 2015. “Effect of Exercise Training on Estimated GFR, Vascular Health, and Cardiorespiratory Fitness in Patients with CKD: A Pilot Randomized Controlled Trial.” *American Journal ofKidney Diseases* 65 (3): 425-434. https://doi.org/10.1053/j.ajkd.2014.07.015.

Guimarães, G. V., E. G. Ciolac, V. O. Carvalho, V. M. D’Avila, L. A. Bortolotto, and E. A. Bocchi. 2010. “Effects of Continuous vs. Interval Exercise Training on Blood Pressure and Arterial Stiffness in Treated Hypertension.” *Hypertension Research* 33 (6): 627-632. https://doi.org/10.1038/hr.2010.42.

Hasegawa, N., S. Fujie, N. Horii, et al. 2018. “Effects of Different Exercise Modes on Arterial Stiffness and Nitric Oxide Synthesis.” *Medicine and Science in Sports and Exercise* 50 (6): 1177-1185. https://doi.org/10.1249/MSS.0000000000001567.

Heydari, M., Y. N. Boutcher, and S. H. Boutcher. 2013. “High-Intensity Intermittent Exercise and Cardiovascular and Autonomic Function.” *Clin Auton Res* 23 (1): 57–65. https://doi.org/10.1007/s10286-012-0179-1.

Holloway, K., D. Roche, and P. Angell. 2018. “Evaluating the Progressive Cardiovascular Health Benefits of Short-Term High-Intensity Interval Training.” *European Journal of Applied Physiology* 118 (10): 2259-2268. https://doi.org/10.1007/s00421-018-3952-6.

Jamka, Małgorzata, Paweł Bogdański, Patrycja Krzyżanowska-Jankowska, et al. 2021. “Endurance Training Depletes Antioxidant System but Does Not Affect Endothelial Functions in Women with Abdominal Obesity: A Randomized Trial with a Comparison to Endurance-Strength Training.” *Journal of Clinical Medicine* 10 (8): 1639. https://doi.org/10.3390/jcm10081639.

Jeong, J., J. D. Sprick, D. R. DaCosta, K. Mammino, J. R. Nocera, and J. Park. 2023. “Exercise Modulates Sympathetic and Vascular Function in Chronic Kidney Disease.” *JCI Insight* 8 (4). https://doi.org/10.1172/jci.insight.164221.

Kim, M., R. Lee, N. Kang, and M. H. Hwang. 2021. “Effect of Limb-Specific Resistance Training on Central and Peripheral Artery Stiffness in Young Adults: A Pilot Study.” *Applied Sciences (Switzerland)* 11 (6). https://doi.org/10.3390/app11062737.

Kobayashi, Ryota, Kenji Asaki, Takeo Hashiguchi, and Hideyuki Negoro. 2022. “Effect of Aerobic Exercise Training Frequency on Arterial Stiffness in Middle-Aged and Elderly Females.” *Journal ofPhysical Therapy Science* 34 (5): 347–52. https://doi.org/10.1589/jpts.34.347.

Koh, K. P., R. G. Fassett, J. E. Sharman, J. S. Coombes, and A. D. Williams. 2010. “Effect of Intradialytic versus Home-Based Aerobic Exercise Training on Physical Function and Vascular Parameters in Hemodialysis Patients: A Randomized Pilot Study.” *American Journal ofKidney Diseases* 55 (1): 88-99. https://doi.org/10.1053/j.ajkd.2009.09.025.

Lai, Y. H., C. H. Wang, H. J. Lin, et al. 2025. “Intradialytic Exercise: Effects on Arterial Stiffness and Gait Speed in Patients Undergoing Hemodialysis.” *Medical Science Monitor* 31: e947604. https://doi.org/10.12659/MSM.947604.

Lee, E., I. Kolunsarka, J. Kostensalo, et al. 2022. “Effects of Regular Sauna Bathing in Conjunction with Exercise on Cardiovascular Function: A Multi-Arm, Randomized Controlled Trial.” *American Journal ofPhysiology. Regulatory, Integrative and Comparative Physiology* 323 (3): R289-R299. https://doi.org/10.1152/ajpregu.00076.2022.

Lee, Y. H., S. H. Park, E. S. Yoon, et al. 2015. “Effects of Combined Aerobic and Resistance Exercise on Central Arterial Stiffness and Gait Velocity in Patients with Chronic Poststroke Hemiparesis.” *American Journal ofPhysical Medicine & Rehabilitation* 94 (9): 687-695. https://doi.org/10.1097/PHM.0000000000000233.

Lin, H. F., S. C. Wang, H. M. Cheng, and J. Sugawara. 2021. “Homebased Standing Core Exercise Training Improves Femoral Blood Flow but Not Arterial Stiffness in Middle-Aged to Older Adults.” *Artery Research* 27 (2): 75-81. https://doi.org/10.2991/ARTRES.K.201222.001.

Liu, G., and X. Zhu. 2025. “The Effect of Table Tennis Combined with Resistance Training on the Rehabilitation of Patients with Coronary Heart Disease.” *Annals of Thoracic Medicine* 20 (3): 160-168. https://doi.org/10.4103/atm.atm_6_24.

Madden, K. M., C. Lockhart, D. Cuff, T. F. Potter, and G. S. Meneilly. 2013. “Aerobic Training-Induced Improvements in Arterial Stiffness Are Not Sustained in Older Adults with Multiple Cardiovascular Risk Factors.” *Journal ofHuman Hypertension* 27 (5): 335-339. https://doi.org/10.1038/jhh.2012.38.

Magalhães, J. P., X. Melo, I. R. Correia, et al. 2019. “Effects of Combined Training with Different Intensities on Vascular Health in Patients with Type 2 Diabetes: A 1-Year Randomized Controlled Trial.” *Cardiovascular Diabetology* 18 (1). https://doi.org/10.1186/s12933-019-0840-2.

Mawdsley, D., M. Bennetts, S. Dias, M. Boucher, and N. J. Welton. 2016. “Model-Based Network Meta-Analysis: A Framework for Evidence Synthesis of Clinical Trial Data.” *CPT: Pharmacometrics & Systems Pharmacology* 5 (8): 393–401. https://doi.org/10.1002/psp4.12091.

Moncion, K., L. Rodrigues, B. De Las Heras, et al. 2024. "Cardiorespiratory Fitness Benefits of High-Intensity Interval Training After Stroke: A Randomized Controlled Trial." *Stroke* 55 (9): 2202-2211. https://doi.org/10.1161/STROKEAHA.124.046564.

Morgan, B., A. M. Mirza, C. J. Gimblet, et al. 2023. "Effect of an 11-Week Resistance Training Program on Arterial Stiffness in Young Women." *Journal ofStrength and Conditioning Research* 37 (2): 315-321. https://doi.org/10.1519/JSC.0000000000004280.

O'Gorman, S. A., C. T. Miller, J. C. Rawstorn, et al. 2023. "Sex Differences in the Feasibility of Aerobic Exercise Training for Improving Cardiometabolic Health Outcomes in Adults with Type 2 Diabetes." *Journal of Clinical Medicine* 12 (4). https://doi.org/10.3390/jcm12041255.

Okamoto, T., Y. Hashimoto, and R. Kobayashi. 2018. "Effects of Interval Walking Training Compared to Normal Walking Training on Cognitive Function and Arterial Function in Older Adults: A Randomized Controlled Trial." *Aging Clinical and Experimental Research*.

Okamoto, T., M. Masuhara, and K. Ikuta. 2013. "Low-Intensity Resistance Training after High-Intensity Resistance Training Can Prevent the Increase of Central Arterial Stiffness." *International Journal ofSports Medicine* 34 (5): 385-390. https://doi.org/10.1055/s-0032-1312604.

Oliveira, E. Silva V. R., F. Stringuetta Belik, J. C. Hueb, et al. 2019. "Aerobic Exercise Training and Nontraditional Cardiovascular Risk Factors in Hemodialysis Patients: Results from a Prospective Randomized Trial." *Cardiorenal Medicine* 9 (6): 391-399. https://doi.org/10.1159/000501589.

Oliveira, G. H. de, P. Boutouyrie, C. F. Simões, et al. 2020. "The Impact of High-Intensity Interval Training (HIIT) and Moderate-Intensity Continuous Training (MICT) on Arterial Stiffness and Blood Pressure in Young Obese Women: A Randomized Controlled Trial." *Hypertension Research : Oficial Journal ofthe Japanese Society ofHypertension* 43 (11): 1315–18. https://doi.org/10.1038/s41440-020-0477-2.

Omar, N., B. S. Yeoh, K. Chellappan, S. Z. Chui, N. Salamt, and A. Aminuddin. 2023. "The Effects of Pedometer-Based Exercise on Central and Peripheral Vascular Functions among Young Sedentary Men with CVD Risk Factors." *Frontiers in Physiology* 14. https://doi.org/10.3389/fphys.2023.1062751.

Pascoalino, L. N., E. G. Ciolac, A. C. Tavares, et al. 2015. "Exercise Training Improves Ambulatory Blood Pressure but Not Arterial Stiffness in Heart Transplant Recipients." *Journal ofHeart and Lung Transplantation* 34 (5): 693-700. https://doi.org/10.1016/j.healun.2014.11.013.

Petrick, H. L., T. J. King, C. Pignanelli, et al. 2021. "Endurance and Sprint Training Improve Glycemia and V˙O2peak but Only Frequent Endurance Benefits Blood Pressure and Lipidemia." *Medicine and Science in Sports and Exercise* 53 (6): 1194-1205.

Reed, K. S., A. M. Frescoln, Q. Keleher, A. G. Brellenthin, M. L. Kohut, and W. K. Lefferts. 2024. "Effects of Aerobic Exercise Training on Cerebral Pulsatile Hemodynamics in Middle-Aged Adults with Elevated Blood Pressure/Stage 1 Hypertension." *Journal of Applied Physiology (Bethesda, Md.: 1985)* 136 (6): 1376-1387. https://doi.org/10.1152/japplphysiol.00689.2023.

Scott, S. N., M. Cocks, R. C. Andrews, et al. 2019. "High-Intensity Interval Training Improves Aerobic Capacity Without a Detrimental Decline in Blood Glucose in People With Type 1 Diabetes." *Journal of Clinical Endocrinology and Metabolism* 104 (2): 604-612. https://doi.org/10.1210/jc.2018-01309.

Shiotsu, Y., Y. Watanabe, S. Tujii, and M. Yanagita. 2018. "Effect of Exercise Order of Combined Aerobic and Resistance Training on Arterial Stiffness in Older Men." *Experimental Gerontology* 111: 27-34. https://doi.org/10.1016/j.exger.2018.06.020.

Slivovskaja, I., L. Ryliskyte, P. Serpytis, et al. 2018. "Aerobic Training Effect on Arterial Stiffness in Metabolic Syndrome." *American Journal of Medicine* 131 (2): 148-155. https://doi.org/10.1016/j.amjmed.2017.07.038.

Stewart, K. J., A. C. Bacher, K. L. Turner, et al. 2005. "Effect of Exercise on Blood Pressure in Older Persons: A Randomized Controlled Trial." *Archives ofInternal Medicine* 165 (7): 756-762. https://doi.org/10.1001/archinte.165.7.756.

Taylor, J. L., S. E. Keating, D. J. Holland, D. J. Green, J. S. Coombes, and T. G. Bailey. 2022. "Comparison of High Intensity Interval Training with Standard Cardiac Rehabilitation on Vascular Function." *Scandinavian Journal ofMedicine & Science in Sports* 32 (3): 512-520. https://doi.org/10.1111/sms.14106.

Teixeira Do Amaral, V., A. A. Viana, A. D. Heubel, et al. 2022. "Cardiovascular, Respiratory, and Functional Effects of Home-Based Exercise Training after COVID-19 Hospitalization." *Medicine and Science in Sports and Exercise* 54 (11): 1795-1803. https://doi.org/10.1249/MSS.0000000000002977.

Ter Veer, Emil, Martijn G. H. van Oijen, and Hanneke W. M. van Laarhoven. 2019. "The Use of (Network) Meta-Analysis in Clinical Oncology." *Frontiers in Oncology* (Switzerland) 9: 822. https://doi.org/10.3389/fonc.2019.00822.

Thompson, S., N. Wiebe, M. K. Stickland, et al. 2022. "Physical Activity in Renal Disease and the Effect on Hypertension: A Randomized Controlled Trial." *Kidney & Blood Pressure Research* 47 (7): 475-485. https://doi.org/10.1159/000524518.

Tomoto, T., J. Liu, B. Y. Tseng, et al. 2021. "One-Year Aerobic Exercise Reduced Carotid Arterial Stiffness and Increased Cerebral Blood Flow in Amnestic Mild Cognitive Impairment." *Journal ofAlzheimer's Disease* 80 (2): 841-853. https://doi.org/10.3233/JAD-201456.

Tomoto, T., A. Verma, K. Kostroske, et al. 2023. "One-Year Aerobic Exercise Increases Cerebral Blood Flow in Cognitively Normal Older Adults." *Journal of Cerebral Blood Flow and Metabolism* 43 (3): 404-418. https://doi.org/10.1177/0271678X221133861.

Valkenhoef, Gert van, Sofia Dias, A. E. Ades, and Nicky J. Welton. 2016. "Automated Generation of Node-Splitting Models for Assessment of Inconsistency in Network Meta-Analysis." *Research Synthesis Methods* (England) 7 (1): 80–93. https://doi.org/10.1002/jrsm.1167.

Van Craenenbroeck, A. H., E. M. Van Craenenbroeck, K. Van Ackeren, et al. 2015. "Effect of Moderate Aerobic Exercise Training on Endothelial Function and Arterial Stiffness in CKD Stages 3-4: A Randomized Controlled Trial." *American Journal ofKidney Diseases* 66 (2): 285-296. https://doi.org/10.1053/j.ajkd.2015.03.015.

Watt, Jennifer, Andrea C. Tricco, Sharon Straus, Areti Angeliki Veroniki, Gary Naglie, and Aaron M. Drucker. 2019. "Research Techniques Made Simple: Network Meta-Analysis." *The Journal ofInvestigative Dermatology* (United States) 139 (1): 4-12.e1. https://doi.org/10.1016/j.jid.2018.10.028.

Way, K. L., A. Sabag, R. N. Sultana, et al. 2020. "The Effect of Low-Volume High-Intensity Interval Training on Cardiovascular Health Outcomes in Type 2 Diabetes: A Randomised

Controlled Trial.” *International Journal of Cardiology* 320: 148-154. https://doi.org/10.1016/j.ijcard.2020.06.019.

Wheeler, David C., DeMarc A. Hickson, and Lance A. Waller. 2010. “Assessing Local Model Adequacy in Bayesian Hierarchical Models Using the Partitioned Deviance Information Criterion.” *Computational Statistics & Data Analysis* 54 (6): 1657–71. https://doi.org/10.1016/j.csda.2010.01.025.

Williams, A. M., J. K. Ma, K. A. Martin Ginis, and C. R. West. 2021. “Effects of a Tailored Physical Activity Intervention on Cardiovascular Structure and Function in Individuals With Spinal Cord Injury.” *Neurorehabil Neural Repair* 35 (8): 692–703. https://doi.org/10.1177/15459683211017504.

Yoshizawa, M., S. Maeda, A. Miyaki, et al. 2009. “Effect of 12 Weeks of Moderate-Intensity Resistance Training on Arterial Stiffness: A Randomised Controlled Trial in Women Aged 32-59 Years.” *British Journal of Sports Medicine* 43 (8): 615-618. https://doi.org/10.1136/bjsm.2008.052126.

Zhang, Y., M. Ou, M. Cheng, X. Ying, H. Hu, and M. Korivi. 2025. “Effects of Moderate-Intensity Resistance Training on Vascular Endothelial Function and Arterial Stiffness in Young Healthy Men.” *Journal of Strength and Conditioning Research* 39 (8): e960-e966. https://doi.org/10.1519/JSC.0000000000005124.
